# Posters

**DOI:** 10.1155/1990/29589

**Published:** 1990


P0001 TRANSPORTAL EMBOLISATION OF ESOPHAGEAL VARICOSITY IN PROFUSE BLEEDINGS IN PATIENTS WITH PORTAL HYPERTENTION

Sh.Karimov, Medical Institute,
Tashkent, USSR

In profuse esophageal bleedings a conservative therapy has little efficacy and a surgical intervention is attended by high risk for the patient's life.

A promising trend in treatment of this portal hypertension complication is roentgenendovascular surgery which attracts surgeons with its lesser traumatism and organ preservation capacities.

The results of management in 61 patients with such a complication who underwent percutaneous transhepatic roentgenendovascular interventions are presented. Portal hypertension development was due to intrahepatic block in 54 patients, extrahepatic one in 3, and a mixed block in 4 cases.

Extremely severe blood loss (above 50% of circulating blood volume) was noted in 36 cases and a medium degree (25%CBV) was observed in 9 patients. All in all 154 therapeutic catheter endovascular interventions were performed. The technique and stages of these interventions will be presented in the report.

**P0002** INHIBITION OF THE ACTIVITY OF PROTEO-LYTIC ENZYMES OF HUMAN PANCREATIC JUICE BY ETHANOL AND ACETALDEHYDE

Z.Puchalski,E.Skrzydlewska,J.Prokopo-wicz,K.Worowski,I.Zakrzewska,
Z.Piotrowski

Department of General Surgery,Medical Academy, Białystok, Poland

The purpose of this work to determine the in vitro effect of ethanol and acetaldehyde on the activity of proteolytic enzymes of human pancreatic juice. Pancreatic juice was collected from 10 patients during surgery performed because of chronic pancreatitis or pancreatic cyst. The effect of ethanol and acetaldehyde on total proteolytic activity of pancreatic juice was estimated by use of casein. The effect of this substances on trypsin activity was estimated on benzyl-arginyl-p-nitroanilide and chymotrypsin activity on N-/3-carboxy-propionyl/-phenyl-alanyl-p--nitroanilide. Ethanol and acetaldehyde decrease the activity of trypsin and chymotrypsin of pancreatic juice. The decrease of the activity of both enzymes is proportional to their concentrations. Inhibitory effect of acetaldehyde is markedly higher than that of ethanol.Possible mechanism of inhibition of the activity of trypsin and chymotrypsin by studied substances as weel as clinical implications concerning alcohol and pancreatic disease are discussed.

**P0003** SURGICAL TREATMENT OF CHRONIC PANCREATITIS BY PYLORUS-PRESERVING PANCREATODUODENECTOMY

Z.Puchalski,Z.Piotrowski,J.Okulczyk,

Z.Sokołowski

Department of General Surgery Medical Academy, Białystok, Poland.

95 patients with chronic pancreatitis of different intensity wery treated operatively in the years 1980-1989. Nearly complete resection of the pancreas with duodenum according to Traverso and Longmire in our own modification was carried out in 12 patients (12.6%) with most intensive inflammatory changes covering the head and corpus of the pancreas. About 5 cm of the pancreatic tail was usually left and it was tightly closed by sutures or the duct was filled with ethiblock (Ethicon). The common biliary duct was implanted into the jujunum about 15-20 cm from the intestinal anastomosis. The pancreas was completaly resected in one patient (1.05%) with the biggest pathologic changes and diabetes. The patients were observed clinically during 5-14 days for the symptoms of impaired gastric emptying and the were followed radiologically for 2-6 weeks.

On the basis of our observations we suggest that above method seems to be better concerning the late results in patients with advanced chronic pancreatitis specially located in the head and corpus of the pancreas.

### P004 CURATIVE TREATMENT IN LOCOREGIONAL RECURRENCE AFTER PANCREATIC- AND PERIAMPULLARY CANCER

J.H.G. Klinkenbijl, M.B.E. Menke-Pluymers,
M. Tjioe, P.I.M. Schmitz, J. Jeekel.
Erasmus University Rotterdam, Rotterdam,
The Netherlands.

The prognosis for patients with carcinoma of the pancreas is poor, even after curative resection. If locoregional recurrence develops after resection, no further treatment is considered in general. The question arises if any form of treatment can improve survival in case of locoregional recurrence. For this purpose, a retrospective study was done.
During the period 1978-1988, 106 patients underwent an in purpose curative resection in the University Hospital Rotterdam-Dijkzigt, 58 for carcinoma of the pancreas and 48 for periampullary carcinoma. In 34 patients (20 with carcinoma of the pancreas and 14 with periampullary carcinoma) locoregional recurrence was found (incidence 32%), after a disease-free interval of 15 months average (range 5-37 months). 68% Of the patients presented with upper abdominal pain and 62% with weight loss. Only in 50% of the patients with locoregional recurrence distant metastases were found. Survival after diagnosis of locoregional recurrence was considerably better for the group of 17 patients without distant metastases (0,4-76 months, mean 11 months), in comparison with the group of 17 patients with locoregional recurrence and distant metastases (0,3-11 months, mean 3 months), p=0,06. 4 Patients with locoregional recurrence without proven metastases were treated. In 1 patient the tumor-recurrence could be resected radically, in 1 patient irradical, after which irradiation and chemotherapy were given, 2 patients were treated by chemotherapy alone.
The mean survival in the treated group was 33 months (range 8-76 months), significant longer than patients with locoregional recurrence without distant metastases who were not treated (0,4-7 months, mean 4 months), p=0,009.
The longest survival was found after radical resection of locoregional tumor-recurrence in this retrospective study (after 76 months still alive). For patients with locoregional recurrence without distant metastases after in purpose curative resection of carcinoma of the pancreas or periampullary region, treatment should be considered.

**P0005** SUPGICAL TREATMENT OF HEPATIC HYDATID CYSTS WITH INTRABILIARY RUPTURE

G.Bonatsos, E.Leandros, B.Zombolas,

G.Panetsos, P.Peveretos, B.Golematis

First Dept. of Propaedeutic Surgery,Athens University, Greece.

During the years 1981-89, 26 patients with intrabiliary rupture of hepatic hydatid cysts underwent surgical treatment in our Institution. Almost all the patients presented with jaundice, abdominal pain and fever. Differentiating this complication from gallstones is sometimes difficult clinically and biochemically. Nevertheless, ultrasound and CT scanning are most helpful in establishing the correct diagnosis.

At laparotomy, the two main goals were to evacuate the mother cyst and to remove all hydatid material from the biliary tree. The patency of the biliary system was always ascertained by intra-operative cholangiography. The mother cyst was treated with partial cystectomy, omentoplasty (whenever omentum was available) and tube drainage.

For the permanent decompression of the biliary tract a wide choledochoduodenostomy was performed in 18 patients, whilst sphincteroplasty was performed in three cases. Finally, in five cases the common bile duct was closed over a T-Tube; this should be done only in emergency operations in high risk patients where shortening the time of the operation is crucial. Nevertheless, persistent postop bile drain resulted in three patients of the last group, prolonging their overall time of hospital stay. In a follow-up period of 1-10 years five patients were presented with attacks of cholangitis and one more was reoperated on for this picture.

In conclusion, our experience confirms that the principles of operative management in hepatic hydatid cysts with intrabiliary rupture are: to treat the mother cysts as in uncomplicated cases, to clear the biliary tree of any parasitic material and to secure a permanent free biliary drainage with a proper procedure, preferably a choledochoduodenostomy.

**P0006** TUMORS OF THE BILIARY TRACT: OBSERVATIONS FROM 93 CASES.

Marrano D., Greco V.M., Casadei R., Viti G.
Istituto di 1^ Clinica Chirurgica della
Università degli Studi di Bologna (Italy)
(Dir.: Prof. Domenico Marrano)

At the Institute of the 1st Surgical Clinic and Surgical Therapy in the period from 1975 to 1989,93 patients affected with malignant tumors of the extrahepatic biliary tract were treated surgically.
The localization of the neoplasm was, in 23 cases the hepatic hilum, in 9 cases the gallbladder, in 17 cases the gallbladder with infiltration of the common bile duct, in 12 cases the choledochus, and in 30 cases the papilla. Two tumors were spread to the entire extrahepatic biliary tree. A radical therapy was possible in 52.7% of the cases. In particular, 5 hepatobiliary resections were performed for the tumors of the hilum (index of resectability = 21.7%), 8 cholecistectomies (88.9%) (2 associated with wedge hepatic resection) for the gallbladder tumors. For the gallbladder tumors infiltrating the common bile duct, 5 hepatic resections were performed (29.4%).
The tumors localized in the choledochus were excised by biliary resection or duodenocephalopancreatectomy in 4 cases (33.3%).
The index of resectability for the tumors of the papilla was 90% (14 D.C.P. and 13 duodenoampullectomies). The postoperative mortality was 12.9%: 7 patients after palliative or exploratory operations, 5 after excisional operations. The average survival was 3 months after explorative operation, whereas after palliative operation it was 4.1 months for the hilar tumors, 6 months for the gallbladder tumors, 28.2 months for the tumors of the choledochus, 21 months for the papillary tumors. Long-term survival was good for the gallbladder tumors (only 1 death after 2 years) and bad for those infiltrating the common bile duct (7 months average survival). For the hilar tumors excised, 2 patients died, 1 after 7 months and 1 after 3 years. The other 3 are living after 3 years with follow-up from 6 months to 2 years. For the tumors of the choledochus excised, 2 patients died, 1 at 10 and 1 at 24 months. 2 others are living after 1 year. Of the patients operated for D.C.P., 8 died after an average of 22.1 months and 4 are living with follow-up from 1 to 5 years. The average survival of the 5 patients who died after ampullectomy was 24.6 months while 6 patients are living after 6 months to 6 years.

**P0007** 150 HEPATIC RESECTIONS WITH THE HELP OF ULTRASONIC DISSECTOR

E. Gadžijev, V. Pegan

University dep. of gastroenterologic surgery, Ljubljana, Yugoslavia

During the last 3 years we are using ultrasonic dissector for liver operations, and in these period we performed 150 liver resections for the pathology as follows:

50 for hydatide cysts, 26 for colorectal liver metastases, 22 for hemangiomas, 16 for primary liver malignances, 9 for hilar holangiocarcinomas and carcinomas of gallblader, 9 for metstases other than colorectal, 5 for benign liver tumors, 5 for patologic changes after liver trauma, 4 for liver abscesses and 4 for other conditions.

In tabulary form the data of patients, operative procedures, duration of operations, "ultrasonic time", blood loss, morbidity and mortality are presented. The 30 days mortality was 0, and the complications occured in 20 % of cases.

Ultrasonic dissector was very usefull in pericystectomy so we performed in 80 % total pericystectomies - without opening the cyst. The utility of the ultrasonic device was proved also in excisions of hemangiomas which were performed with minimal blood loss.

We conlude that the ultrasonic dissector opened the possibilities of performing liver resections safely and with good results also for non-prominent liver surgeons.

### P0008 EXPERIENCE IN THE DIAGNOSIS OF HYDATID DISEASE OF THE LIVER

Xu Ming Qian
Research Unit of Hydatidology of
The People's Hospital of Xinjiang, Urumqi
People's Republic of China

Hydatid disease is a common endemic parasitic malady which prevails in pastoral areas of the world, and affects both animals and man. A total of 1390 patients were treated by operations between 1953-1988. Of these, 1002 cases had hydatid cyst in the liver.

Diagnosis: 1. A history of contact with dogs or their contaminated environment. 2. A sense of occult pain in the liver region. 3. An enlarged liver and/or large cyst projecting under the liver may be palpable 'Hydatid thrill'. 4. Complicated infection of hydatid cyst leading to liver abscess. Complicated rupture into hepatic duct causing cholecystalgia and/or obstructive jaundice and AOSC. Rupture into the abdominal cavity producing acute diffuse peritoniits, anaphylactic shock and disseminated implantation of protoscolice and daughter cysts. Rupture into the lung causing hydatid cyst-pulmonary abscess-bronchial fistula. 5. Immunodiagnosis: Casoni's test, IHA, ELISA, LA, McAb has a high specifity and sensitivity. 6. Ultrasonic exploration, radioisotopic scanning, Roentgenography, CT and PTC are useful for diagnosis.

P0009 IMMEDIATE NON-SURGICAL DECOMPRESSION OF THE BILIARY TRACT IN SEVERE ACUTE CHOLANGITIS.

R. Masetti, R.Coppola, M.E.Riccioni,
P.Magistrelli, A.Picciocchi
Catholic University of Rome - Italy

Aim of this study was to evaluate the efficacy of immediate non-surgical decompression of the biliary tract in reducing mortality and morbidity in patients with acute cholangitis.
The study was conducted over the two-year period from November 1987 to October 1989. Fourty-eight consecutive patients with clinical evidence of severe acute cholangitis entered the study (25 men and 23 women, age range 20-87 years, mean age: 67 years).
Patients were first evaluated by ultrasonography and direct cholangiography (ERCP in 46 cases and PTC in 2) that showed the etiology of cholangitis to be due to choledocholithiasis in 33 cases, malignant biliary obstruction in 6, benign biliary stricture in 6 and hydatid liver disease in 3.
Non-surgical decompression of the biliary tract was then attempted in all cases and successfully completed in 46 patients (96%). In the two failures emergency surgery was performed.
Complete resolution of the sepsis was achieved in 43 of the 46. In 23 of these patients delayed elective biliary surgery was successively performed while in the other 20 no further treatment was deemed necessary. In 3 patients sepsis persisted in spite of non-surgical biliary decompression; 2 underwent emergency surgery (1 death) and 1 recovered following medical treatment.
Overall mortality and morbidity rates were respectively 2.1% (1/48) and 19% (9/48).
These results confirm the efficacy of immediate non-surgical decompression of the biliary tract in patients with severe acute cholangitis. It allows resolution of the sepsis in a high percentage of cases (89% in this study), avoids the need for emergency surgery, and may represent a definitive treatment in cases due to choledocholithiasis or in high risk patients.

### P010 HETEROTOPIC AUTOTRANSPLANTATION OF CANINE LIVER SEGMENTS

J. Ringers, T.M. Karsten, L.T. de Wit,
T.M. van Gulik, M.N. van der Heyde
Academic Medical Centre, Amsterdam NL

Auxiliary transplantation of a segmental liver graft, a modification of the heterotopic autotransplantation model according to Van der Heyde (1) was studied. The left liver lobes were isolated, maintaining their arterial supply and bile outflow, and transposed to the right subhepatic space. The portal branch was anastomosed with the vena mesenterica superior and venous outflow was provided by anastomoses with the inferior vena cava just above its bifurcation. The remaining right lobes had a functional handicap compared with the transplanted left lobes, due to ligation of artery and bile duct. Six dogs were operated upon. Two dogs died on day 22 and 107 of bile leakage from the remaining right liver lobes and a perforated duodenal ulcer. One dog had to be sacrificed on day 12 due to complete obliteration of the left hepatic duct. Serum transaminase levels were elevated during the first two weeks and normalized thereafter. All dogs showed a wedged venous hepatic pressure gradient of the graft of less than 5 mm Hg, therefore showing no evidence of portal hypertension at the sinosoidal or postsinosoidal level. 99mTc HIDA scintigraphy evidenced graft functioning with significantly shorter T 1/2 values in comparison with the right liver lobes. Bile duct proliferation was found in biopsies from the right liver lobes, as a result of extrahepatic obstruction. Biopsies of the transplanted left lobes showed hyperplasia of the liver cell plates as evidence of regeneration.
In conclusion the presented segmental autotransplantation model is shown to be valuable for the study of the physiologic and hemodynamic changes associated with auxiliary liver transplantation, independent of the rejection phenomena. Auxiliary transplantation on the inferior vena cava does not result in a relative venous outflow block and therefore does not result in portal hypertension.

Reference :
(1) Van der Heyde, M.N. and Schalm, L. Brit J Surg 1968; 55: 114

### P0011 EFFICIENCY OF TWO DIFFERENT SURGICAL APPROACHES TO BILIARY LITHIASIS

A.M.Almeida,F.Aldeia,C.Gracias,N.M.Santos
University Hospit.Sta.Maria,Lisbon,Portugal

Surgery may still be the simplest way of dealing with biliary lithiasis(1,2).Hrein we compare the long term results of two approaches,based on different concepts of the natural history of biliary calculi,to surgery of biliary lithiasis:one assuming most CBD stones form primarily in the GB(THEORY I),another believing the majority of duct calculi originate in the duct itself,consequent to a malfunctioning SO(3)(THEORY II).**METHODS:**680 cholecystect.were performed by followers of THEORY I(Group A)and 438 by followers of THEORY II(Group B)during 1980-89.Age,sex distribution and preop. evaluation were similar.In Group A,assuming that once the GB is removed and the duct cleared the problem is solved,OPC was a routine?criteria for CBDE were as classically defined(3)and T-tube decompressionof explored ducts was widely utilized:162 CBDE's(24%)-T-tube 79(49%),C-D 57(35%),C-J 21(13%),SPT 5(3%).In Group B OPC was selective and every duct > 10mm was explored and definitively decompressed,except three( < 10mm):80 CBDE's(18%)- T tube 3(4%),C-D 66(83%),C-J 6(7%),SPT 5(6%).Long term results were classified as previously described(4,5),POOR result meaning the necessity of either resurgery or EST for residual/recurrent stones and/or associated pathology.Statistical analysis by Student's T test. **RESULTS:**As outlined :

| | Group A | Group B | Signif. |
|---|---|---|---|
| Operative Mortality | 0.9% | 0.7% | NS |
| Operative Morbidity | 15% | 7% | NS |
| % Positive CBDE's | 65% | 70% | NS |
| % POOR(non-expl.ducts) | 1.5% | 0% | NS |
| **% POOR(explored ducts)** | *** 8%** | **1.25%** | **P=0.001** |

* All occurring among T-tube decompressed ducts.

**CONCLUSIONS:** Routine OPC does not enhance the positivity of CBDE's,decreasing its efficiency.It is hardly justifiable on that basis.The policy of exploring every duct > 10mm does not lead to an increased rate of "unnecessary" explorations.By definitively decompressing(abolishing or bypassing the malfunctioning SO)all dilated biliary trees,the problem of residual/recurrent stones,a complication still plaguing T-tube decompressed ducts(6),is attenuated without,necessarily,increasing the mortality.The most efficient approach seems to be based on THEORY II.**REFERENCES:1)Br.J.Surg,1987;74:555,2)Ann.Surg,** 1988;207:135,3)Surg.Clin.N.America,1973;53:1095,4)Am.J.Gastroent, 1982;77:941,5)Am.J.Surg,1984;147:253,6)Br.J.Surg,1987;74:1095

**P0012** KLATSKIN'S TUMORS: IS AN ONCOLOGICALLY RADICAL PROCEDURE POSSIBLE?

A.Principe, M.Spangaro, G.Gozzetti
2° Dept. of Surgery, University of Bologna, Italy

The prognosis of biliary tract tumors, especially of those at hepatic hilum level, depends on a curative resection which is conditioned by the site, nature and extension of the lesion. The patient selection and the indication for a correct management stems from an accurate preoperative work-up, such as ultrasonography, PTC, ERCP, CT scan and angiography. The intraoperative ultrasonography, aids the decisions making and engineers radical surgery by giving further useful information on the diffusion of the disease and the relationship with the main intra-hepatic vessels. During the last 8 yrs 37 pts. with Klatskin's tumors have been admitted to our Unit: 26 males, 11 females, average age 65 yrs. (range 35/91). Jaundice was the first symptom in 87% of the cases. The average time lapse betwen onset of symptoms and diagnosis was 3 months. A radical procedure was carried out in 14 patients (resectability 37,8%): the tumor with the bile duct bifurcation was resected in 7 pts (in 2 along with a Wedge-Resection) and an hepatectomy was performed in the others seven. In 19 cases the treatment was only palliative and 4 patients were beyond all surgery. There was no operative mortality in the radical surgery series. Six patients (46%) are still alive and free of disease between 3 and 96 months from surgery (3 hepatectomies and 3 tumor resections). The average survival after resection of the confluence with Wedge-Resection was 8 months while in the hepatectomy series (4 cases) it was 28 months. The average survival after palliative derivations was 7,8 months.

CONCLUSIONS:

1 - in Klatskin's tumors the prognosis doesn't depend only on surgical radicality, which is really difficult to obtein, but it is also related to the histology grading and staging of the lesion.
2 - patients submitted to liver resection have the best long term prognosis with a good quality of life.

**P0013** PREVENTION OF GASTROINTESTINAL BLEEDING AFTER PANCREATICODUODENECTOMY WITH SOMATOSTATIN

M. MOROSINI
I° DEPARTMENT OF SURGERY, MARTINI HOSPITAL
TURIN (ITALY)

Haemorrhage is a life-threatening complication that can occur,despite several improvements in pre- and post-operative management,after pancreatic resection for malignant diseases.It contributes to raise significantly patient morbility and mortality. The reported incidence varies from 5 to I9 per cent with a mortality of 6-58 per cent. Between I970 and I988 we treated 375 cases of ampullary and pancreatic head cancers. 100 patients (37,5 %) underwent radical treatment (pancreaticoduodenectomy with Child reconstruction).

To reduce the risk of gastrointestinal bleeding from 1984 we usually treat the patients,after the surgical procedure,with continuous intravenous infusion of somatostatin (250 mcg/hour) for 72 hours, beginning at the same time of the operation.

Before starting with this medical prophylactic treatment morbility and mortality rate (63 cases) related to mayor digestive bleeding were 10 per cent and 26 per cent respectively. From 1984 up to december 1988 37 cancers were resected with only 2 post-surgical haemorrhages (5,4 %) and no mortality,performing the same surgical procedure, without vagotomy.

According to our experience we believe that somatostatin could be a successful prophylactic drug in the prevention of gastrointestinal bleeding after pancreatic cancer resection.

REFERENCES:

Trede M., Schwall G. Ann.Surg. I988,207:39
Shankar S.,Russell RCG. Br. J. Surg. I989,76:863
Meinke WB.et al. AM.J.Surg. I983,146:57
Herter FP. et al. Ann.Surg. 1982,I95:274
Dixon JM. et al. Ann.Surg.I984,I99:271
Scott HW. et al. Ann.Surg.I980,191:688

### P0014 THE PLACE OF COMPUTED TOMOGRAPHIC SCAN IN MANAGEMENT OF BILIARY ACUTE PANCREATITIS

F Sebai
70 Avenue Habib Bourguiba, Rades, Tunis
Tunisia

From 1985 to 1989 80 patients with necrotic acute pancreatitis have been treated in the general surgery service of La Rabta Hospital (Tunis). 40 patients have been followed by computed tomographic scan.

- 50% of patients had more than 2 Ranson's early prognostic criteria
- 50% had scans during the first week of their admission to hospital
- 30% had stage C by scanning
- 30% had stage D by scanning
- 15% had stage E by scanning

Correlations between Ranson's prognostic criteria and computed tomography scanning criteria can predict evolution to necrotic abscesses.

**P0015**

### THE INCREASED INCIDENCE OF CHOLELITHIASIS IN THE ALLUVIAL SOIL PEOPLE IN TRIKALA (DEMOGRAPHIC STUDY)

Th.Galeas*,A.Papadopoulos*,E.Galea,I.Vardoulis†* I.Siafas†* G.Tsoutsos†** G. Sagarakis†** A.Valakis,*** A.Vasilopoulos*†* K.Sideris,**** G.Tsianos*** B' Internal Medicine Clinic* Surgical Dept*** X-ray Dept** Trikala General Hospital, Athens Univ.Mineralogy Dept*.***Greece

In this work it is examined whether the soil has any effect on the incidence of cholelithiasis (Len Mervyn 1980,Galeas et al 1989) and in case such an influence is observed whether it is direct or indirect, through population density. The importance of the different kinds of soil on health has been observed since ancient times (Hippokrates works). The county of Trikala is suitable for this study because it is separated into two kinds of soil, alluvial and non alluvial (Katakouzinos S !958) and there has been no emigration of the population in recent years.
Material of the study are the 546 patients who have been hospitalized with cholelithiasis in Trikala General Hosp. from 1-1-83 to 31-12-87. Their place of birth or residence for at least 20 years was noted. It was defined,from suitable maps, if it was on alluvial soils (soils which were formed during the last geological period and are still being formed) or non alluvial soils.Ultrasonography and cholecystography were used for the diagnosis of the disease. Patients were submitted to operation. The X2 method showed that the incidence of cholelithiasis was statistically significantly inreased on alluvial compared to non alluvial soils.

| SOIL | CHOLELITHIASIS | WITHOUT CHOLELITHIASIS | TOTAL POPULATION |
|---|---|---|---|
| ALLUVIAL | 473 | 94467 | 94940 |
| NON ALLUVIAL | 73 | 29033 | 29106 |
| TOTAL | 546 | 123500 | 124046 |
| | X2=62,258 | | |
| | P<0,0005 | | |

In conclusion the alluvial soils in the Trikala area have a relation with the significantly increased incidence of cholelithiasis.This relation seems to be independent of the population density in the two types of soil.

References

Len Mervyn. Minerals and your Health,1980. Katakouzinos S, 1958

P0016 TIME DEPENDENT CONTRAST ENHANCEMENT AS A QUANTITATIVE CT PARAMETER OF EXPERIMENTAL PANCREATIC NECROSIS

W. Maier, M. Buechler, B. Heymer
Depts of Radiology, Surgery and
Pathology of the University of Ulm, FRG

The aim of the study was to correlate the extent of experimental pancreatic necrosis on microscopy with pancreatic enhancement curves on single level dynamic Computed Tomography (CT).

In 17 dogs acute pancreatitis was induced by injecting a watery solution of Na-Taurocholate (10%)-Trypsine into the duct of WIRSUNG. Then, microscopic and CT cuts were performed in exactly corresponding levels of the pancreas.

Correlation between regression analysis of CT time-density curves and the extent of parenchymal pancreatic necrosis on microscopy was extremely close (Correlation coefficient of SPEARMAN -0.90). Moreover, it was possible to study the contrast kinetics of singular pancreatic lobules on CT.

The study shows that the extent of pancreatic parenchymal necrosis can be predicted with great accuracy by quantitatively evaluating single level CT time-density curves.

### P0017 MORPHOLOGICAL CHARACTERISTICS OF PRIMARY LIVER CANCER AND THEIR APPLICATION IN SURGERY

M.Sh.Israelashvili, Tbilisi
State Medical Institute, Tbilisi,
USSR

To investigate macro- and microscopic structure of primary liver cancer as well as its local and distant proliferation, we analysed I5I cases of this disease. To elucidate the objective laws of intrahepatic distribution of primary tumor 54 isolated liver preparations taken from patients who died due to primary liver cancer were thoroughly examined. They were dissected in layers and hepatic tissue was taken for histological examination from every segmental and subsegmental basin of the portal triad (I575 microscopic preparations in total). The results of our investigations may be summarized as follows: I. Among macroscopic forms of liver cancer nodular one was most common (52.3%) followed by massive (35.8%) and diffuse (II.9%) forms of tumor. Liver cell carcinoma was the most common primary liver tumor (78.I%) followed by cholangiocellular cancer. 2. Massive form of tumor was most favourable for surgical intervention. Liver resection in nodular carcinoma, as a rule, is impossible and, when performed, is not radical. In duffise carcinoma operative intervention is not indicated. 3. Oncological considerations require differential approach to cases of liver cancer with intraorganic metastases. Cancer with intrahepatic metastases located beyond the half of the organ affected by primary tumor should be regarded as inoperable one whereas cancer with metastases located within the same anatomical half as primary carcinoma is operable. 4. In primary liver cancer total involvement of the liver was observed in 52.9% of cases. The right portal lobe of the liver was affected in 20.5%, left portal lobe in 9.3%, left caval lobe in 4.6%, isolated sectors in 5.3%, isolated segments in 3.9% and portal fissure area in 3.I% of cases. 5. The most radical operation in liver cancer is hemihepatectomy with the removal of fatty tissue and lymph nodes of the portal fissure and hepatoduodenal ligament.

P0018

### PALLIATION FOR HILAR CHOLANGIOCARCINOMA: A MULTIDISCIPLINARY APPROACH

RH Gompertz, IS Benjamin, A Yip, R O'Sullivan†, A. Adam†, LH Blumgart and RCN Williamson.

Departments of Surgery and †Radiology, Hammersmith Hospital, London.

During the decade 1979 to 1989 195 cases of hilar cholangiocarcinoma were managed at a single specialist unit. The average age at presentation was 58 (21-85) years with a male: female ratio of 10:7. Resectability was assessed on the basis of detailed biliary imaging, with or without laparotomy. Of these 195 patients 79% were deemed irresectable because of local tumour extent or metastasis. Palliative treatment of the 151 irresectable patients is reported. Biliary drainage was achieved in over 90% throughout the course of the series. Of those palliated during the period 1979 to 1983, 78% underwent surgical decompression and 22% stent drainage, whereas from 1984 to 1989, 29% underwent surgical drainage and 71% stent drainage. During the initial period stents were mostly narrow-bore plastic tubes but, for the majority, larger bore stents have been placed and, over the last 18 months self-expending metal stents have been employed for long term drainage. These innovations have meant that stents are now entirely internal in 91%. Radiotherapy with external beam with or without iridium wire implants was used in 24 (16%) in an attempt to inhibit tumour growth and to delay stent blockage with its attendant jaundice and cholangitis. Recurrent jaundice and cholangitis may be less frequent after surgical bypass but stenting offers excellent medium-term palliation for irresectable bile duct cancer in frail patients. Improvements in stent technology have prolonged trouble free function and allowed changing of occluded stents. Patients should be managed at a specialist centre not only to assess resectability but also to allow selection of the optimum technique for each patient, as well as to develop new palliative techniques and to define further the role of adjuvant therapy.

P0019 INDICATION FOR PRIMARY CLOSURE AFTER CHOLEDOCHOTOMY

Y.Nakano,T.Nagakawa,N.Kadoya,T.Nakamura,
H.Kobayashi,K.Maeda,N.Ueda,M.Kayahara,
T.Ohta,K.Ueno,I.Konishi,T.Matsumoto,
I.Miyazaki,The Second Department of
Surgery,School of Medicine,Kanazawa
University,Kanazawa,Japan.

Primary closure after choledochotomy is safe and valuable method when all of the calculi in the common bile duct is removed and the function of papilla Vater is normal.

Since 1973 the function of papilla Vater has been evaluated by the variable loading cholangiomanometry after choledocholithotomy. The indication for primary closure after choledochotomy was set up that less than 7 units($mmH_2O\cdot min/ml$)of resistance and $150mmH_2O$ of intraductal pressure at the operative cholangiomanometry and less than number of three choledochal stones at the preoperative direct cholangiography. The results of primary closure(n=12)were compared with those of T tube drainage (n=54) between the years 1982 and 1988. At the time spent in hospital after the operation,the median time for patients having primary closure of the common bile duct was 14 days. And those patients having a T tube inserted remained with a median of 33 days. there is a significant difference in the length of stay between the two groups,p=0.05. Biliary leak after the removal of T tube occurred in one patient. Transient biliary leak after the operation was seen in one patient having primary closure.

### P0020 GIANT HYDATID CYSTS OF THE LIVER

G.Kyratzis,L.Papastamatiou.V.Skoutzos
Ch.Zografos,Th.Philea.
Dept.of Surgery.Apostle Paul Hosp.-KAT
ATHENS-HELLAS.

According to the data of our National Statistic Organization more than 1000 patients with hydatidosis are hospitalized every year.The liver is the target organ in more than 75% of the cases.Although Hellenic surgery is familiar to echinococcosis,mortality rates remain high (2-3%) depending on various factors.One of the most important factors is the big size of the cyst,causing operative difficulties,intrahepatic rupture of the cyst and intraabdominal reccurence of the parasitosis.

Twelve patients,7 men and 5 women aged 22-77,with giant (>20 cm) hydatid cysts of the liver were treated during the last ten years.Pain was the main symptom,butjaundice, cholangiitis,septic fever and deteriorating clinical picture in three cases of intrahepatic rupture of the cyst were the causes of emergency hospitalization.In seven cases a mass was easily visible at the right hypochondrium. Immunologic investigations were positive in all patients and radiology,ultrasonography and CT-scans confirmed the diagnosis and determined the dimensions of the cysts.The greater diameter ranged from 32 to 21 cm.The operative technique included wide exposure of the liver through abdominal or thoracoabdominal incision,aspiration,incision and careful evacuation of the cyst,partial capsectomy or total pericystectomy sacrificing liver parenchyma up to atypical segmentectomy.Cholecystectomy,exploration of the common bile duct,removal of daughter cysts and debriments and choledochoduodenostomy in the cases of intrahepatic rupture of the cysts was added.Suture of bile communications drainage and omentoplasty of the liver cavity completed the operation in each case.One elderly patient died because of cardiovascular complications and another two were peoperated 4 and 6 years later because of reccurence of the disease.

It is concluded that total pericystectomy is the best operative procedure to prevent morbidity,mortality in elderly patients remains high and reccurence is depended on contamination during aspiration and evacuation of the cyst.

### P0021 EARLY OPERATION FOR ACUTE CHOLECYSTITIS

A. Agorogiannis, I. Agorogianni
Surgical Department, General Hospital of
Larisa, Larisa, Greece

Over a 12 year period, 2220 patients presenting with symptomatic gall stone disease were operated on in our surgical department. In 407 patients (18.33%) urgent and early operation was performed for acute cholecystits (AC). In the treatment of AC both early surgery within a few days of onset and delayed operation after the subsidence of acute symptoms have supporters. However, the majority of general surgeons still treat AC conservatively and prefer delayed operation during a second admission. Our object is to clarify from our own experience this controversial topic of early versus delayed operation.

The mean age of the 407 patients was 58 years. The surgical procedures were 407 cholecystectomies and 53 (13%) choledochotomies. Thirty four patients also had gangrene and perforation of the gallbladder (8%) and 12 patients (2.9%) had biliary peritonitis. In the AC group the proportion of patients over 70 years of age was 42% and in the elective group 11%. All patients with perforation and biliary peritonitis were over 70 years of age. We advocate the 'cystic duct first' technique of cholecystectomy and under difficult circumstances (10%) the 'fundus first' removal of the gallbladder. There were no technical difficulties in the early operations measured by duration of time and operative complications. More blood was lost in the AC group. Patients with gangrene, perforation and biliary peritonitis were all over 60 years and 72% over 70 years. The mortality for elective surgery for gallstone disease in our Institution was 0.22%. The mortality rate for patients with AC operated on early was 2.4%. All patients who died were over the age of 70 years.

The data suggest early operation in patients with AC when performed by an experienced surgeon in a well equipped hospital. It offers lower costs, fewer days in hospital, the advantage of avoiding recurrent attacks during the waiting period (30%) and it protects the patient by more deterioration of his condition in case he is operated on after 72 hours of being admitted. The mortality in our cases was related to the age of the patient. All the patients who died were over 70 years of age and had a complication of their AC, ie gangrene, perforation and bile peritonitis. We conclude that early operation in patients with AC is a safe procedure.

**P0022** A RANDOMISED TRIAL USING BRANCHED CHAIN AMINO ACIDS IN SEVERE LIVER DISEASE

D.C. Hunter, D. Driscoll, S. Randall,
G.L. Blackburn, B.R. Bistrian
Cancer Research Institute, New England
Deaconess Hospital, Harvard Medical School,
194 Pilgrim Road, Boston, Massachusetts.

16 patients with severe liver disease, underwent a prospective randomised trial, to determine the kinetic effects of a diet enriched with branched chain amino acids. All patients received both a branched chain amino acid enriched formula, containing 50% branched chain amino acids (BCA), and a conventional, isocaloric, isonitrogenous formula, containing 19% BCA, in random order, and given parenterally. $^{14}C$ tyrosine was used as an amino acid tracer, to determine the protein kinetics. Tyrosine flux was statistically unchanged at (mean $\pm$ s.d.) 36.89 $\pm$ 13.70 µmol/kg/h on 19% BCA, and 37.96 $\pm$ 14.92 µmol/kg/h on 50% BCA (p = 0.791). Tyrosine oxidation was reduced from 3.93 $\pm$ 1.66 µmol/kg/h on 19% BCA, to 3.01 $\pm$ 2.31 µmol/kg/h on 50% BCA, but this failed to reach statistical significance (p = 0.064). Whole body protein synthesis and the fractional rate of albumin synthesis were also statistically unchanged. Protein synthesis was 32.96 $\pm$ 13.15 µmol/kg/h on 19% BCA, and 34.95 $\pm$ 15.08 µmol/kg/h on 50% BCA, and the albumin synthetic rate was 9.8 $\pm$ 6.13 %/day on 19% BCA and 10.77 $\pm$ 7.51 %/day on 50% BCA (p = 0.625 and p = 0.541 respectively). This study failed to show a significant improvement in protein kinetics, due to the supplementation of BCA, in this group of patients with severe liver disease.

**P0023** *THE RESULTS OF LIVER TRANSPLANTATION (LTx); A COMPARISON BETWEEN THE CHOLEDOCHO-CHOLEDOCHOSTOMY (CCS) AND THE HEPATICO-JEJUNOSTOMY (HJS) FOR BILIARY RECONSTRUCTION*

*MG van Andringa de Kempenaer, J Pruim,*
*IJ Klompmaker, GJ Bonsel, MJH Slooff;*
*Liver Transplant Group Groningen and*
*Institute for Medical Technology*
*Assessment Rotterdam, the Netherlands.*

*The choledocho-choledochostomy (CCS) is the preferred method of biliary reconstruction during LTx. However, in some patients a hepatico-jejunostomy (HJS) is inevitable because of absent or diseased extra-hepatic bile-ducts. This study was performed to investigate if an obligatory construction of a HJS has implications for the patient in terms of complications, liver function and survival. Therefore the results of 110 LTx's surviving for more than one week were analysed. In 26 LTx's a HJS was constructed and in the remaining 84 patients a CCS. Statistical analyses were performed with Student's t-tests, $X^2$-tests and Mann-Whitney U-tests. Naturally in the CCS group PBC and CAC were more prevalent compared to a higher prevalence of biliairy atresia, sclerosing cholangitis and SBC in the HJS group. Also the ages differed significantly ($p<0.05$) between both groups. CCS group median 42 years (0-64); HJS group median 4,5 years (0-46). Both groups did not differ in peroperative blood-loss, operating time, patients needing reinterventions for bleeding and immuno-suppressive regimen. In the CCS group more patients were treated (bolus methylprednisolon) for acute rejection ($p<0.05$).One and two year graft survival was 63 % and 60 % in the CCS group compared to 70 % in the HJS group (n.s). The number of patients with intrahepatic or wound abscesses, cholangitis, sepsis, peptic ulcers, vascular complications, stenosis or leakage from the biliary anastomosis or graft failure did not differ significantly in both groups. However, in the HJS group the incidence of intra-abdominal abscesses (4/26) was significantly higher ($p<0.05$) compared to the CCS group (3/84). The median values of gGT and SGPT did differ significantly ($p<0.05$) at certain time points, however these differences disappeared after the first year.*
*<u>Conclusion</u>: When used in its specific indication fields the HJS offered the same results as the CCS in terms of graft survival and function after liver transplantation. However, a higher incidence of intra-abdominal abscesses was observed when a HJS was used for biliary reconstruction.*

**P0024** LIVER RESECTION OF NON-COLORECTAL SECONDARIES

A. Saarela, R. Andersson, K.-G. Tranberg, S. Bengmark, Department of Surgery, Lund University, Lund, Sweden.

Non-colorectal malignancy appears to account for some 15-25 % of hepatic resections for metastatic disease. The aim of this study was to review our experience of liver resection of non-colorectal secondaries.

All 31 resections of non-colorectal secondaries during 1970-1987 were analyzed retrospectively. The primary tumour was situated in the small bowel (7) (6 carcinoid tumours), gallbladder (6), stomach (5), pancreas (3), skin (3) and at miscellaneous sites (7). 8 tumours grew directly into the liver. In the patients with true metastatic disease (n=23), liver tumours were synchronous in 13, solitary in 11, $\leq$ 4 in 17 and unilateral in 16. The margin of resection was $\leq$ 10 mm in 11 patients. 1 patient had known extra-hepatic disease at the time of liver resection. Survival was analyzed with the Kaplan-Meier technique and differences in actuarial survival were estimated with the Mantel-Haenszel method.

1-, 3- and 5-year survival rates were 71, 39 and 19 % (median survival 37 months), respectively, after inclusion of the operative mortality rate (10 %). The resection margin was the only variable that varied with the outcome: patients with a resection margin $\geq$ 10 mm had a longer median survival (176 months) than patients with a smaller margin (17 months) ($p < 0.001$). Variables that could not be demonstrated to influence survival included site or type of the primary tumour, number of liver metastases, uni- or bilateral disease, tumour size, synchronous or metachronous disease and pattern of spread. The disease recurred in the liver in 15 (48 %) patients and at extrahepatic sites in 11 (35 %) patients.

It is concluded that liver resection of non-colorectal secondaries may give meaningful palliation and occasional cure, if a resection margin of $\geq$ 10 mm is obtained. Further studies are needed for improved definition of resection criteria.

### P0025 IS IT WORTHWHILE TO DRAIN AFTER ELECTIVE CHOLECYSTECTOMY? A PROSPECTIVE STUDY

N C M Mehta, M M Begani, P K Jhawer

Bombay Hospital, Bombay, India

It has been a tradition to drain the subhepatic space after elective cholecystectomy to take away the anticipated discharges of blood, serum and bile. Almost all Indian surgeons strongly recommend routine drainage for this purpose.

To evaluate the efficacy of drains, we made a prospective study of drainage (Group 1) and non-drainage (Group 11) patients after elective cholecystectomy.

Ultrasonography was used to assess the amount of fluid collection in the subhepatic space on the 3rd post-operative day in randomised patients from both groups.

In all, 93 patients underwent cholecystectomy for cholelithiasis between 1986-88 in our unit. 55 patients (Group 1) were drained and in 38 patients (Group 11) drains were not used. Both groups received pre-operative 2 gms Cefazolin and no post-operative antibiotics.

These two groups were compared with respect to post-operative complications, sonographic collections, fever and the duration of hospitalisation.

Post-operative complications occurred in 16/55 patients in Group 1 and 2/38 in Group 11. Wound infection developed in 8 patients (14.5%) in the drainage group as compared to 1 patient (2.63%) in the non-drainage group. Mean hospital stay was 9.2 days in Group 1 and 4.98 days in Group 11. Amongst 26 patients studied, ultrasonography showed 40-50 ml of fluid collection on the 3rd post-operative day in 4 of 14 (28.5%) patients in the drainage group and in 1 of 12 (8.33%) patients in the non-drainage group.

It is concluded that routine drainage after elective cholecystectomy is unnecessary as it increases wound infection, post-operative morbidity and stimulates formation of fluid. Prophylactic use of drains is to be discouraged.

### P0026 EXPERIMENTAL STUDIES ON THE HIGHLY DENERVATED SPHINCTER OF ODDI MOTILITY AND GALL BLADDER CONTRACTION IN DOGS

N.Kadoya, T.Nagakawa, H.Kobayashi, Y.Nakano, T.Nakamura, N.Ueda, K.Maeda, M.Kayahara, T.Ohta, K.Ueno, I.Konishi, T.Matsumoto and I.Miyazaki
Department Surgery II, School of Medicine, Kanazawa University, Kanazawa, Japan

There is an increased incidence of gall stone after gastrectomy or truncal vagotomy. In Japan there is more increased incidence of gall stone after gastrectomy with lymph node dissection of the hepato-duodenal ligament, but the mechanism is unknown. Our aim was to study the early effect of highly denervated sphincter of Oddi(SO) motility and gall bladder(GB) contraction. Dogs were anesthetized with GO and pancronium bromide(n=5). Microtransducers (Nippon Koden Inc.) were inserted into the GB and SO to record simultaneously the SO motility and GB contraction in 60 minutes before and after administration of caerulein(10ng/kg iv., 0.2μg/kg iv.)(controls). After the denervation SO motility and GB contraction were recorded samely, the extent of denervation was around the common bile duct, portal vein, hepatic artery, gastro-duodenal artery and lesser omentum (denervated dogs).

In controls, caerulein administered 10ng/kg induced GB contraction but did not changed SO motility. Caerulein 0.2μg/kg induced GB contraction and increased SO motility. In denervated dogs, gall bladder contraction time delayed and SO motility decreased (systolic pressure 46.5±3.1→ 32.4±7.9mmHg, diastolic pressure 26.3±1.8→17.1±3.3mmHg, frequency 11.3±3.1→5.6±1.0/min.). Furthermore, in denervated dogs caerulein administered 0.2μg/kg decreased the excitation of SO motility.

In conclusion, highly denervation of sphincter of Oddi and gall bladder decreased SO motility and delayed gall bladder contraction. It is suspected that these results may induce bile stasis, biliary infection and finally gall stone formation.

P0027 THE REFLECTABILITY OF HISTOLOGICAL TUMOR SPREAD BY PREOPERATIVE ANGIOGRAPHY IN GALLBLADDER CARCINOMA

T. Kaiho, M. Miyazaki, H. Itoh, H. Suzuki,

T. Shimoda, K. Okui, T. Suwa*

1st Dept. of Surgery, Chiba University, Japan
*Ohmiya Red Cross Hospital, Japan

In the choice of operative procedures for gallbladder carcinoma, hepatic parenchymal invasion (h-inf) and bile duct invasion (b-inf) could be important factors, which would also reflect the post-operative prognosis.
This study was aimed to evaluate the reflectability of pre-operative angiographic findings to the histological tumor spread, especially h-inf and b-inf in resected cases of gallbladder carcinomas.
Twenty-three resected cases of gallbladder carcinomas were studied and the following results were obtained.

1) Dilatation of the cystic artery was seen in 75% of h-inf(-) and 67% of h-inf(+) cases. On the contrast, encasement of the cystic artery was found in 80% of h-inf(+) cases more frequently than in 13% of h-inf(-) cases ($p<0.01$).
2) Encasement of intrahepatic arteries and portal venous branches were demonstrated only in h-inf(+) cases, but not so frequent as 60% in artery and 40% in portal vein.
3) Tumor staining could be seen in both h-inf(-) and (+) cases, as 50% and 67%.
4) Encasement of cystic artery trunk were especially seen in 88% of b-inf(+) cases, but none of b-inf(-) cases ($p<0.01$).

In conclusion, preoperative angiographic findings is demonstrated to reflect preciously the histologic tumor invasion to the liver and biliary tract.

### P0028 ADOPTIVE IMMUNOTHERAPY IN HEPATOMA

S.H.Choi, T.S.Lim, B.R.Kim, K.I.Park,

K.S.Lee, K.S.Hahm, D.S.Na, M.H.Han

Yonsei University & KIST, Seoul, Korea

Application of adoptive immunotherapy has been the inability to generate specifically sensitized lymphoid cells with antitumor reactivity. Interleukin-2(IL-2) is now available in large quantities and has made possible expansion of cells with a diverse spectrum of immune reactivities. In this study, we evaluated the immunologic effects and the response rate of lymphokine activated killer(LAK) cell and IL-2 administered intravenously in humans with hepatoma unresponsive to other treatments. There were three primary hepatomas and four metastatic hepatomas. Two patients received LAK cell and IL-2 twice. $5.94 \times 10^9 \pm 2.74 \times 10^9$cells were obtained in each leukapheresis. Cellular Cytotoxicity was measured using 51-Chromium release assay.

| | NK function | P | LAK function | P |
|---|---|---|---|---|
| Pretretment | 37.15 ± 20.83* | NS | 4.46 ± 3.65 | |
| 1st week | 36.70 ± 20.68 | NS | 31.88 ± 25.22 | <0.01 |
| 2nd week | 43.82 ± 21.61 | NS | 41.80 ± 28.79 | <0.01 |
| 3rd week | 45.33 ± 30.82 | NS | 50.87 ± 26.54 | <0.01 |

* % cytotoxicity at E/T ratio = 100:1

These data clearly show that LAK and IL-2 treatment augmented LAK cell activity, but there were no statistically significant differences between pretreatment and posttreatment in NK function. There were one complete response and one partial response. It could be speculated that LAK cell and IL-2 infusion can be used as an adjuvant treatment for the hepatoma.

**P0029** BILIARY TRACT COMPLICATIONS IN LIVER TRANSPLANTATION

E. MORENO; I. GARCIA; R. GOMEZ; I. GONZALEZ; H. BONET; C. LOINAZ; J. BERCEDO; J. IBAÑEZ; P. RICO.

Hospital 12 de Octubre. Madrid. Spain.

During 1986-1989, the Servicio de Cirugía Aparato Digestivo II of "12 de Octubre" Hospital (Madrid, Spain), carried out 108 Liver Transplants (LT). In 91 patients, just one LT was performed and in 17 Two or more LT. 75 Patients were adults and 16 children.
MATHERIAL AND METHODS: Biliary reconstruction (BR) was as follows:

| GROUPS | ADULTS | CHILDREN |
|---|---|---|
| A(Choledocho-choledocho) | 72(66,6%) | 7(6,48%) |
| B(Choledocho-jejunostomy) | 13(12,03%) | 14(12,96%) |
| C(Colecist-Hepatic-jejunostomy) | 1(0,92%) | 1(0,92%) |
| TOTAL | 86 | 22 |

In all cases the BR was made in monoplane with vicryl(R) suture 5-0 During post-operative period were performed the following periodical controls: Lab. Test, Bacteriology, Liver biopsy, and other complementary methods were used (Doppler ultrasound, HIDA TM99, Trans-T-Tube Cholangiography, ERCP, and arterigram.
RESULTS: B.R. COMPLICATIONS:

| GROUPS | FISTULA | OBSTRUCTION | STRICTURE |
|---|---|---|---|
| A | 3 | - | 1 |
| B | 2 | 1 | - |
| C | - | - | - |

BILIARY COMPLICATIONS AFTER HEPATIC ARTERY THROMBOSIS (HAT)

| GROUP | BILIARY NECROSIS | BILOMA | FISTULA | STRICTURE |
|---|---|---|---|---|
| A | 3 | 1 | 1 | 2 |
| B | - | 2 | 1 | 1 |
| C | - | - | - | - |

Evident necrosis of intra and extra hepatic bile ducts was always proved in the cases with (HAT). There was no mortality due to complications in BR.
COMMENTS: Direct duct to duct (Choledocho-choledochostomy) is the preferred methods of BR. The Choledocho-jejunostomy anstomosis is the BR choice when the bile duct is unsuitable for direct reconstruction.

P0030

### BILE SALTS ALTER ISOLATED KUPFFER CELL MORPHOLOGY WITHOUT LOSS OF VIABILITY

S. Singh, M.E. Bailey

University of Surrey and The Royal Surrey
County Hospital Guildford, Surrey, UK.

There is an increased incidence of post operative renal failure in obstructive jaundice; this is due to systemic endotoxaemia. Clearance of endotoxin is a function of kupffer cells and this has been shown to be impaired in obstructive jaundice. (Bradfield 1974, Bailey 1976, Pain *et al* 1987).

Rat kupffer cells were isolated using in-situ perfusion techniques with enzymatic dispersion (Page and Garvey 1979). Cell Yield was 2-8x$10^6$ cells per gram of liver. Viability at plating as assessed by trypan blue exclusion was 60-90%, purity of cultures was 90-95% as shown by latex phagocytosis and peroxidase staining. After a 24hr recovery period, these cells were exposed to Cholic and Chenodeoxycholic acid at concentrations of 50-200µg/ml. The cells were examined after 48hrs. Viability was then assessed by the 3-(4,5-dimethyl-thiazol-2-yl)-2, 5-diphenyl tetrazolium bromide (MTT) assay and morphology by Electron Microscopy.

Although there were no changes in viability the morphological changes were pronounced. There was formation of plasma membrane blebs together with a reduction in the number of microvilli, dilatation and vacuolisation of the rough endoplasmic reticulum, degeneration of mitochondria and the appearance of a lucent zone around the nucleus. These changes appeared at the highest bile salt concentrations. This finding is in direct contrast to those previously reported (Van Bossuyt *et al* 1989).

These changes suggest that bile salts have a profound effect on kupffer cells and may be responsible for the change in function in obstructive jaundice.

### References

Pain JA, Collier DStJ, Ritson A Eur. Surg. Res 1987; 19: 16-22.

Bradfield JWB Lancet 1974; ii: 883-886.

Page DT, Garvey JS J of Immunological methods 1979; 27: 159-173.

Van-Bossuyt H, Desmaretz C, Gaeta GB, Wisse E Virchows Archiv B Cell Pathology 1989; 57: 141-147.

### P0031 POST-CHOLECYSTECTOMY DUCT DILATATION - MYTH OR REALITY ?

P. Sungler, M. Heinerman, O. Boeckl

Ist Surg. Dept. and Ludwig-Boltzmann-Inst.
Landeskrankenanstalten Salzburg, Austria

Duct dilatation to a maximum of 10 mm in ultrasound was regarded as a so-called physiological reaction of the biliary system due to functional changes of the bile flow.

In a retrospective study we evaluated the sonographical results of 208 patients with a post-cholecystectomy syndrome. All these patients had ultrasound before ERCP or operative procedures. The findings were validated by ERCP in 206 patients, by operation and intraoperative cholangiography in 2 patients.

In group A (n = 134) we regarded a diameter of common bile duct less than 10 mm after cholecystectomy as normal. Compared with the findings in ERCP sonography only had a sensitivity of 36.1% and a specificity of 82.7%. Because of the unsatisfying results of ultrasound we changed the criteria. In group B (n = 74) duct dilatation over 6 mm was considered to be indicative for pathology of the CBD. In group B sensitivity was 80%, specificity was 78.9%.

Conclusion:
In contrary to the ultrasound literature we only found a physiological dilatation of the common bile duct after cholecystectomy in 1.9%, in all the other cases dilatation was caused by pathology such as retained stones, papillary stenoses, atypical surgical stomata after transduodenal sphincteroplasty and cholecystectomy, iatrogenic bile duct strictures or - undetectable by sonography - a long cystic stump.

### P0032 ENDOSCOPIC EMBOLIZATION OF BILIARY FISTULAE

JEJ Krige, PC Bornman,
SJ Beningfield, J Terblanche

Surgical Gastroenterology and
Departments of Surgery and Radiology,
University of Cape Town, Groote Schuur
Hospital and MRC Liver Research Centre,
Cape Town, South Africa

A new technique using endoscopic embolization to occlude persistent complex external biliary fistulae arising from peripheral intrahepatic bile ducts was used in 3 patients (hydatid cyst: 2; amoebic liver abscess:1) in whom other endoscopic manoeuvres had failed.

METHODOLOGY: An Olympus JF 1T10 side-viewing duodenoscope and high resolution fluoroscopic monitoring was used. The fistula was identified by retrograde cholangiography using a proximally inflated balloon catheter to enhance distal duct and fistula opacification. The JF 1T10 duodenoscope was replaced by a larger 4.2 mm channel TJF 10 operating duodenoscope to facilitate embolization. An endoscopically placed guide-wire and coaxial catheter combination were selectively steered as far peripherally as possible to allow positioning of a larger 10F polyethylene catheter over the coaxial catheter near the fistula. The coaxial catheter and guide-wire were removed and a 2x2x20 mm Ivalon pledget inserted and pushed along the larger 10F catheter by the guide-wire and coaxial catheter and delivered to block the duct. Complete occlusion of the duct was confirmed by a selective intrahepatic cholangiogram. Antibiotics were given for 48 hours.

Despite embolization of relatively large peripheral ducts in 3 patients, no further alteration in measured liver function occurred. No cholangitis or liver abscess developed and no fistula has recurred. Accurate placement of a catheter as close as possible to the fistula before embolization was facilitated by using high resolution imaging equipment. Selective endoscopic biliary embolization may offer a definitive alternative to major surgical intervention in complex biliary fistulae arising from the liver when other endoscopic alternatives have failed.

### P0033 PROTAGONISM OF THE ARTERIAL CIRCULATION IN THE PRESERVATION OF THE LIVER

L.LORENTE, J.ARIAS, M.A.ALLER,

J.C.RODRIGUEZ, J.I.TROBO, H.DURAN

Hospital San Carlos, Madrid, SPAIN.

The preservation of the liver wich is to be used in a transplant is generally done, when dealing with human beings, using the method of simple perfusion and hypothermic preservation. This method is widely used as it is simple and economic, but it effectiveness is limited. In the different technical varieties that have been described, one can see the existence of a protagonism of the perfusions by via portal, either because they have been done first as they sometimes obviate the arterial perfusion or because the latter was done in an indirect way throught the aorta and thus its effectiveness was not controlled.

A modified technique to perform the succesive perfusions of the liver that are necessary for its preservation by the simple perfusion method and hypothermic storage is presented.

This technical variety has been tested on Large-White pig's livers and consists in doing succesive perfusions of cooling, preservation and washout, first through the hepatic artery and after through the portal vein.

The macroscopic and biochemical characteristics of the effluents obtained through the infrahepatic inferior vena cava during the perfusion-washout of the livers at the end of the period of cold ischemia shows its effectiveness. Likewise, the hepatic perfusion-washout begun via the arterial and finished via the portal vein one avoids the post-revascularization hyperpotassemia in the recptor animals.

A hypothesis is proposed that the arterial protagonism of the perfusion when the simple perfusion and hypothermic storage method of hepatic preservation is used could be a prophylaxis against the complications of a post-transplant biliary origin.

P0034 VASCULAR PROBLEMS IN THE RESECTION OF PANCREATIC AND PERIAMPULLARY TUMORS

Serio G., Iacono C., Zamboni G.*, Bergamo Andreis I.A.°,
Trivisone M., Zicari M.°, Modena S., Girardi C., Dagradi A.
Departments of Surgery, Pathology* and Radiology°
University of Verona - Verona - Italy

Surgical resection of the pancreas due to its tumoral involvement can be hampered or made impossible by the presence of vascular problems, namely anomalies of a vessel (Rong,1987) or its direct involvement by the neoplasm itself (Freeny, 1988) .

Between 1970 and 1988, 21 vascular anomalies were detected out of 208 surgical resections of the pancreas in case of pancreatic or periampullary tumors (161 pancreatoduodenectomies; 46 distal pancreatectomies and one total pancreatectomy); they involved the origin or course of a vessel (2 of the hepatic artery; 18 of the right and accessory hepatic arteries; one of the portal trunk). In 17 cases (8 pancreatic carcinomas -PC- 4 periampullary carcinomas -PAC- 2 non functioning islet cell tumors -NFIT- 2 cystadenocarcinomas -CAK- 1 papillary cystic tumor -PCT- ) there was direct vascular involvement (11 of the portal-mesenteric axis; 2 of the superior mesenteric artery; 2 of the right hepatic artery with origin from the superior mesenteric artery; one of the common hepatic artery; one of simultaneous involvement of both superior mesenteric artery and vein. In all 21 patients presenting a vascular anomaly radical surgery was possible, being in only two cases necessary to resect the right hepatic artery. There was no intraoperative mortality, being the complications limited to one case of gastrointestinal hemorrhage due to bleeding of the right accessory hepatic artery : this case was treated with embolization. Among the 17 patients with direct vascular involvement, the resection of the portal-mesenteric axis - performed in 11 cases- was followed by end-to-end anastomosis in 7 cases; mesenterico-caval end-to-side anastomosis in one case; prosthetic replacement in two cases and ligature of the vessel in one case. The resection of the superior mesenteric artery, involved in two cases, was followed by reconstruction with end-to-end anastomosis in both eveniences. The common hepatic artery, resected once, was rebuilt with a prosthesis; the same happened with the resected superior mesenteric artery and vein- simultaneously involved and rebuilt with two prostheses. There were in this group 2 (12%) intraoperative deaths due to hypovolemic shock; the only complication was a massive ascites. Mean survival was been 8 months (range 3-12) for PC and PAC and 88,7 months (range 43-216 ) for NFIT, CAK and PCT.

The Authors are of the opinion that there are no real contraindications to pancreatic resection in case of vascular anomalies; the obstructions can be overcome by thorough dissection. On the contrary, resective surgery in case of direct vascular involvement is not supposedly able to greatly affect the poor prognosis of pancreatic cancer. Nevertheless, vascular resections can be justified in case of slowly growing tumors (PCT, CAK, NFIT).

References :

Rong G.H., Sindelar W.F. : Amer. Surgeon 1987,53 (12), 726.

Freeny P.C., Marks W.M., Ryan J.A., Traverso L.W. : Radiology 1988, 166 (1), 125.

**P0035** ARE PATIENTS WITH OBSTRUCTIVE JAUNDICE DUE TO CARCINOMA AT RISK FOR SURGERY ? A PROSPECTIVE MULTICENTER TRIAL.

M. Kracht, G. Fourtanier and the French Association for Surgical Research (A.R.C.), Colombes, France

The role of jaundice in postoperative survival and morbidity in patients with malignant biliary tract obstruction is controversial.The aim of this prospective study was to determine possible prognostic factors influencing the immediate outcome after surgical treatment of this condition. 290 patients with jaundice due to a neoplastic obstruction (180 pancreatic carcinoma, 88 biliary tract carcinomas, 22 ampullar tumours) underwent either an excision (51) or a bypass (223) of the tumour. 16 patients had explorative laparotomy only. Were recorded for all patients: age,weight loss, ASA score, duration of jaundice,temperature, bilirubin, BUN, creatinin, albumin, transaminases, prothrombin time, alkaline phosphatases, hematocrit and leukocyte count. Results were assessed according to immediate mortality (56) and morbidity (169 patients ). Prognostic factors were analyzed according to univariate and multivariate discriminative analysis and were correlated to the postoperative outcome. Factors related to operative mortality were: ASA score,bilirubin, BUN, creatinin, prothrombin time, and preoperative leukocytosis. Discriminative analysis allowed to classify correctly 77 % of patients at risk for postoperative death when the following formula was positive: 0.05 x age + 0.92 x ASA score +0.004 x bilirubin - 0.02 x prothrombin time + 0.0001 x leukocyte count -5.4. Prognostic factors for postoperative complications were: ASA score, bilirubin, leukocyte count and prothrombin time.The following formula, when positive, alowed to classify correctly 61 % of patients at risk for postoperative complications: 1.5 - o.49 x ASA score - 0.0026 x bilirubin.
This study suggests that patients with high ASA scores, high bilirubin and long prothrombin times are at risk for surgery and might benefit from a non-surgical treatment.

**P0036** MARKED ELEVATION OF SERUM TRANSAMINASE IN PATIENTS WITH ACUTE GALLSTONE DISEASE

M. Isogai, K. Hachisuka, A. Yamaguchi
Department of Surgery, Ogaki Municipal Hospital, Ogaki, Japan

Marked elevation of serum transaminase has been reported in patients with acute cholecystitis,[1] choledocholithiasis[2)-5)] and gallstone pancreatitis, but its mechanism is not well understood yet. We have tried to clarify its etiology and pathogensis by studying the relationship between the presence of bile duct stones, the time course of serum glutamate-oxaloacetate transaminase (SGOT) levels and the histological findings of liver biopsy specimens in gallstone patients with SGOT or serum glutamate-pyruvate transaminase (SGPT) over 300 Karmen units. Our study has suggested that marked elevation of serum transaminase in patients with acute gallstone disease is derived from acute inflammatory liver cell damages caused by impacted bile duct stones.

We have proposed a new clinicopathological entity of gallstone hepatic injury or gallstone hepatitis to clinically describe high transaminase elevation in patients with acute gallstione disease.

Gallstone hepatic injury seems to be of clinical value: (1) Acute cholangitis is a potentially life-threatening complication of gallstones that results from concurrent biliary infection and obstruction. Thus, a diagnosis of cholangitis should be considered whenver there are signs of infection and gallstone hepatic injury. (2) Gallstone pancreatitis is thought to be caused by the migration of a stone into or through the ampulla of Vater. Accordingly, gallstone hepatic injury may be nescessary as a strict diagnostic criteria for assessing gallstones as the cause of gallstone pancreatitis. (3) Acute cholecystitis should be considered as the inflammation of the gallbladder secondary to obstruction of the bile duct when there is gallstone hepatic injury.

References
1) Adames J.T. et al. Surgery. 1970, 68: 492.
2) Mossberg S.M., Ross G. Gastroenterolgy. 1963, 45: 345.
3) Shora W., Dnovitch S.H. Gastroenterol. 1969, 61: 575.
4) Ginsberg A.L. Digestive Diseases. 1970, 15: 803.
5) Anciaux M.L. et al. Digestive Disease and Sciences. 1986, 31: 449.
6) Mayer A.D., McMahon M.J. Ann. Surg. 1985, 201: 68.

### P0037 PREOPERATIVE DETECTION OF A CAPSULE IN HEPATOCELLULAR CARCINOMAS

JL MEAKINS, R LEJEUNE, F KEMENY, C SMADJA, D FRANCO. Groupe de Recherche sur la Chirurgie du Foie et de l'Hypertension Portale, Hôpitaux Louise Michel, Evry, Bicêtre, Le Kremlin Bicêtre, Paul Brousse, Villejuif, France.

It is generally admitted that encapsulation by a thick capsule is a favorable prognostic factor after resection of a hepatocellular carcinoma (HCC). The purpose of this work was to determine if this could be detected preoperatively by usual liver imaging techniques. Twenty-two consecutive patients with a HCC were studied. An ultrasonography (US) and an angiscan were performed in all patients by the same radiologist. The presence of a complete uninterrupted, or uncomplete halo around the cancerous nodule was looked for separately by two independent investigators. This halo was hypoechogenic on US. It was sponteneously visible and/or visible after a bolus injection of contrast medium in the CT scan. The presence of a thin ( $< 1$ mm) or thick ( $\geqslant 1$ mm) capsule on the resected specimen was noted by a pathologist unaware of radiological results. Results were as follows :

| | US halo | | | Angiscan halo | | |
|---|---|---|---|---|---|---|
| | complete (9) | uncomplete (7) | none (6) | complete (9) | uncomplete (7) | none (6) |
| Thick capsule (10) | 6 | 2 | 2 | 9 | 1 | 0 |
| Thin capsule ( 5) | 0 | 3 | 2 | 0 | 3 | 2 |
| No capsule ( 7) | 3 | 2 | 2 | 0 | 3 | 4 |

Preoperative ultrasonography was not a good procedure to detect an encapsulation of HCC. The sensitivity and specificity of CT-scan with bolus injection in predicting the presence of a thick capsule around the tumor were respectively 90 % and 100 %.
These results suggest that liver scan with bolus injection is valuable in detecting a thick capsule around HCCs. Liver scan with bolus injection is of great importance when resection of a HCC is contemplated.

P0038 **ASSOCIATION BETWEEN GRANULOCYTE ELASTASE, ENDOTOXIN AND POSTOPERATIVE COMPLICATION AFTER HEPATECTOMY**

K. Hamazaki, H. Mimura, K. Orita
Okayama University Medical School, Japan

Within recent years, granulocyte elastase (PMN-E), endotoxin (Et) and tumor necrosis factor (TNF) have received considerable attension for their possible roles in multiple organ failure.
We studied the association of these parameters with postoperative complications after hepatectomy. The blood concentrations of PMN-E, Et, TNF, fibronectin (Fn), fibrinogen and C-reactive protein (CRP) were examined to clarify the participation of postoperative complication in twenty patients who underwent hepatectomy.
PMN-E increased significantly ($p<0.05$) on the second postoperative day compared to preoperative levels, and decreased on the seventh postoperative day.
PMN-E was positively correlated ($p<0.05$) with CRP and white blood cell count.
Et changed similarly as PMN-E, but no positive correlation was found between Et and PMN-E. Et did not show significant positive correlation with the volume of hepatic resection, the amount of blood loss during operation or CRP.
The elevation of TNF considered to be produced by Kupffer cells by the stimulation of Et, could not be found after hepatectomy.
In summary, PMN-E and Et increased significantly after hepatectomy. The elevation of PMN-E and Et do not necessarily mean the occurrence of complications. However, in cases with adult respiratory distress syndrome developed after infection or anastomotic leak PMN-E and Et increased significantly. Thus possibly, PMN-E and Et may be largely responsible for this condition.

References

David G.H. S.G.O 1988: 166: 147
Michael A.W. Surgery 1986: 100: 416

**P0039** THE TROPHIC EFFECTS OF OBSTRUCTIVE JAUNDICE AND BILE DUCT DIVERSION IN RATS --WITH SPECIAL REFERENCE TO GASTROINTESTINAL HORMONES--

S.HIGASHIDE, K.INOUE, M.AMI,
P.L.RAYFORD*, T.TOBE
First Department of Surgery,
Faculty of Medicine,
Kyoto University, Kyoto, Japan
*University of Arkansas for
Medical Sciences, Litle Rock, AR, USA

This study was conducted to explain the mechanism of the trophic effects of obstructive jandice (OJ) and bile duct diversion (BD) on gut organs of rats. Method: A.Control: laparatomy only (n=9); B.OJ: bile duct transected and end separated by 1cm (n=10); C.BD: bile diverted to urinary bladder (n=11). Seven days after surgery, rats were fasted for 24 hrs, sacrificed, the pancreas, stomach and duodenum removed. DNA and RNA in the pancreas were measured and immunoreactive gastrin, CCK and secretin were measured in extracts of the stomach and duodenum.

Results: *:P<0.05

| | mg/100gBW | | | |
|---|---|---|---|---|
| | Stomach | Duodenum | Pancreas | RNA/DNA |
| A. | 659±68 | 334±33 | 428±30 | 5.1±0.6 |
| B. | 676±70 | 441±28* | 608±28* | 7.0±1.3* |
| C. | 616±73 | 419±35* | 554±52* | 6.0±0.5* |

| content (ng) | Gastrin Stomach | CCK Duodenum | Secretin Duodenum |
|---|---|---|---|
| A. | 803±119 | 45.3±5.7 | 21.9±1.2 |
| B. | 787±94 | 51.3±3.4* | 22.4±1.5 |
| C. | 531±226* | 43.2±11.0 | 21.0±3.5 |

Conclusion: 1) In OJ, the trophic effectrs may be associated with elevated levels of duodenal CCK. 2) In BD, the trophic effects may be associated with suppression of G-cell activity in the stomach. 3) Neither OJ nor BD affected duodenal stores of secretin. 4) Results suggest that the trophic effects of OJ and BD are not due to disturbance of kidney and liver functions.

**P0040** FOLLOW UP STUDY ON CHANGES OF GALL-BLADDER FUNCTION AND ENDOGENOUS CHOLECYSTOKININ FOLLOWING GASTRECTOMY

K.Inoue, S.Sumi, S.Higashide, A.Fuchigami, R.Doi, H.Kaji, M.Yun, H.Minote, K.Takaori, R.Hosotani, K.Uchida, T.Tobe

First Department of Surgery, Faculty of Medicine, Kyoto University, Japan

It is widely known that gallbladder dysfunction or gallstone formation is frequently observed in gastrectomized patients. This study was conducted to investigate on the mechanism for this phenomenon with special reference to the release of cholecystokinin (CCK).

Methods: In gastrectomized patients, presense of gallstones were examined serially before and 1, 6 and 12 months after operation. Pre-operative patients with gastric cancer (n=9), 1 month postgastrectomized patients (n=6) and 12 months postgastrectomized patients (n=9) were ingested with a 200ml of fatty meal. Sonograms of the gallbladder were obtained by use of a realtime ultrasound unit at 10 min intervals for 120 min. Blood samples were drawn at the same intervals for specific radioimmunoassay of CCK.

Results: Gallstones developed in 7(14.6%) of 48 patients. Fasting gallbladder area, measured postoperatively, were increased significantly compared to preoperative values. Significant correlation was observed between gallbladder contraction and plasma levels of CCK in both before and after gastrectomy. The integrated CCK response was significantly increased in both 1 month (630±151 pmol-min/L) and 12 months (521±73 pmol-min/L) after gastrectomy compared to pre-gastrectomy (219±68 pmol-min/L). Gallbladder kinetics was completely changed after gastrectomy, showing no contraction phase but early beginning of refilling.

Conclusions: This study demonstrates that CCK plays an important role in changes of gallbladder kinetics after gastrectomy, which might possibly lead to the dysfunction of gallbladder or gallstone formation.

### P0041 THE MANAGEMENT OF ACUTE CHOLECYSTITIS IN THE ELDERLY

M. Ninomiya, S. Kagawa, H. Mimura
*1; Kohki Hospital, Yamaguchi, Japan
*2; Okayama University Hospital,
Okayama, Japan

Acute cholecystitis in the elderly is a serious and often a critical disease, which is in contrast to the mild subjective and objective findings. We studied 27 cases of acute cholecystitis in patients over 70 years old who had been treated at our hospital from January 1985 to May 1989.

RESULTS; (1) Sixty-three percent of the patients showed abasia by themselves. Anticipated infection, mainly in urinary tract, was recognized in 41% of the patients, and 22% of the cased were complicated by diabetes. These are considered to be risk factors for onset of this disease. (2) Subjective complaints at the first onset were nonspecific and included low-grade fever in 82%, loss of appetite in 74% and abdominal symptoms in only 37%. (3) Ultrasonography demonstrated a gallbladder dilatation in 91%, sludge in 78%, a thickning of the gallbladder wall in only 30% and cholelithiasis in only 39%. (4) The survival rate in the treated groups was 0% in the cases with conservative treatment (0/3), 88% with emergency surgery (7/8), 73% treated by urtrasonography guided percutaneous transhepatic gallbladder drainage (PTGBD)alone (8/11) and 100% in the cases with elective cholecystectomy after treatment by PTGBD (5/5). However the death cases treated by emergency surgery or by PTGBD alone were diagnosed tardily so that they were septic at the time of treatment.

CONCLUSION; (1) When aged patients show right hypochondrial tenderness and resistence as well as low-grade fever and loss of appetite established diagnosis should be tried using ultrasonography.
(3) Emergency surgery or PTGBD followed by elective surgery should be performed with minimal delay following diagnosis.

P0042 BLUNT LIVER TRAUMA: AN UPDATE

S. Hanna, F. Brenneman,
G. Pagliarello, B. Ghazarian
Sunnybrook Medical Centre, Toronto, Canada

At the 2nd World Congress on HPB Surgery we presented our experience with Blunt Liver Trauma from June 1, 1976 - June 30, 1987 (Hanna & Scarth 1988). We decided to update this experience to see if there were any changes in our morbidity or mortality and report upon our total group of patients. Between June 1, 1976 and June 30, 1989 the Regional Trauma Unit at Sunnybrook Medical Centre in Toronto, Canada received 3730 patients. Over that 13 year period 335 patients (9%) sustained a liver injury. 318/335 were due to blunt trauma (95%). Open peritoneal lavage was performed on 80% of our patients (267/335), 99% being true positive.

A laparotomy was performed on 97% of our patients (324/335). Major surgical treatment was required in 33% of patients (108/324) and minor surgical treatment in 67% of patients (216/324). 3 patients were treated non-operatively and 8 patients died during resuscitation. Morbidity directly related to the liver trauma was seen in 29 of the 249 surviving patients (11%). Reoperation was required in 14/249 mainly for abscess or hematoma (11/14).

Overall mortality was 26% (86/335). Overall 83% of patients (n=276) had a grade I, II or III liver trauma according to Moore's classification (Moore 1985) with a mortality of 12% (n=32). The remaining 17% of patients (n=59) had a grade IV or V liver trauma with a mortality of 44% (n=26). Of the 86 deaths, head injury accounted for 48 (56% of deaths), liver hemorrhage for 17 (20%), liver sepsis for 1 (1%) and other causes for 20 deaths (23%). Death due to the liver injury itself (hemorrhage and sepsis) occurred in 18 patients (6% overall). The remaining 68 patients (20% overall) died due to other causes but mainly head injuries. There were no major changes in our morbidity or mortality over the past 2 years.

References:

Hanna S, Scarth H: Netherlands J of Surgery - Abstracts 1988 FP226
Moore FA et al: Am J Surg 150: 725, 1985

### P0043 THE VALUE OF SALINE CHOLEDOCHOMANOMETRY IN DETECTING UNSUSPECTED CHOLEDOCHOLITHIASIS:

T K Malik, M Diwakar, D Ashok Singh, R Malik
M A M C, New Delhi, India

This study aims to reduce the incidence of missed CBD stones and unnecessary choledochotomies during cholecystectomy. 119 patients entering the study underwent cholecystectomy along with n. saline choledochomanometry. Average opening pressure of 10 cm saline (range 9.5-17 cm); resting pressure of 13.2 cm saline (range 8-22 cm) and flow rate of 12.5 ml/min (8-16 ml/min) were observed. 41 patients were observed to have abnormal pressures and flow rates. Abnormal opening pressure of 29.2 cm (range 15-47 cm); resting pressure 26-46 cm (range 14-40 cm) and flow rates 4-7 ml/min (range 0-12 ml/min) were observed in these 41 patients. The differences in readings between the normal and abnormal group were statistically significant (p 0.001).

Compared with clinical indications, manometry helped avoid 27 (22.6%) unnecessary choledochotomies. In 15 patients, there was no clinical indication for choledochotomy (12.6%). There were 8 (6.72%) false positives and one false negative (0.84%). The overall accuracy of the technique was 94.11%. The average time taken for the procedure was 10 minutes. Choledochomanometry is simple, quick and easy to perform, and remarkably reduces the incidence of unnecessary choledochotomy and unsuspected CBD stones.

But, in order to reduce the incidence of these problems to near zero choledochomanometry needs to be combined with peroperative cholangiography and/or choledochoscopy.

References:

1. Corlette M B, et al: Operative cholangiography & overlooked stone, Arch Surg 1978, pp 133-729

2. Colcock B P, Liddle H V: Common bile duct stones, N Eng J Med 1958, pp 258-264

### P0044 CHOLECYSTECTOMY WITHOUT DRAINAGE:

A Prospective randomized study.

Z.Yılmaz, E.Sözüer, Y.Yeşilkaya, Y.Arıtaş, N.Bengisu

Department of Surgery,Erciyes University, Medical School,Kayseri,TURKEY

A randomized prospective study was planned in our clinic to investigate if the routine usage of drain after elective cholecystectomy was necessary or not. The effectiveness of drainage and non-drainage was investigated by ultrasonography of the gallbladder area on the third postoperative day.

Our group consisted of 172 women (86 %) and 28 men (14 %). The mean age of the group was 47.4 with a range between 22 and 78. In 100 of the patients no drain was used while a penrose drain was used in 100 of them. The two groups were compared with respect to postoperative complications and duration of hospitalization. In the patients without drains the hospitalization duration at the ranged from 3-8 days with a mean of 4.8 days. On the other hand, in the drained group the hospitalization length was 4-14 days with a mean of 6.4 days. The difference was statistically significant. There were 2 and 6 wound infection in the undrained and drained group respectively (2.5 %) and (6 %). No collection in the gallbladder bed were detected on the third postoperative day by ultrasonography in all the patients.

Finally, we believe that the usage of drain following uncomplicated cholecystectomies will increase the hospitalization duration and postoperative complication rate.

### P0045 EARLY DIAGNOSIS AND MANAGEMENT OF PORTAL VEIN THROMBOSIS FOLLOWING SPLENECTOMY FOR MYELOPROLIFERATIVE DISORDERS

A. Czerniak, A. Judich, A. Kessler
E. Shemesh, F. Graif, A. Ayalon.
The Sheba Medical Center, Tel-Aviv, Israel

Patients undergoing splenectomy for myeloproliferative disorders have an increased risk of thromboembolism. The occurrence of portal vein thrombosis (PVT) in these patients is a rare, often fatal complication.

During the years 1988-1989, 12 patients with myeloproliferative disorders underwent splenectomy as a part of their management. Patency of the portal system was evaluated by ultrasonic doppler duplex system (US) preoperatively and routinely after the operation. PVT developed in 3 patients (myelofibrosis-2; hairy-cell leukemia-1) at 7,8 and 17 days after splenectomy. One patient was asymptomatic and the diagnosis of PVT was made on the basis of the US study only while the other two patients presented with rapid onset of ascites which was associated with abdominal pain in one. All patients had mildly raised serum alkaline phosphatase. Patients were treated with full dosage heparin followed by oral anticoagulants. Repeated US studies demonstrated in all patients the development of portal collateral circulation within a few days. Recanalization of the portal thrombus occurred in one patient. Patients were followed-up for 2, 4, and 18 months, at which time they were asymptomatic but with mildly raised serum alkaline phosphatase.

The routine and repeated use of US study after splenectomy in patients at risk of developing PVT, enables early detection and successful management of this often fatal complication.

### P0046 POST-TRAUMATIC BILE FISTULAE

M. Hollands, T. Wilson, J. M. Little.
Westmead Hospital, Sydney, Australia.

Bile leakage after liver injury has been reported to be a complication associated with significant morbidity and mortality yet little has been written about its incidence, natural history or prognosis. The aim of this study was to address these questions.

Data on 306 patients with liver injuries presenting over a 10 year period to January, 1989 were collected prospectively.

13 patients (4%) developed bile leaks. There were 2 groups of patients - those with major bile duct injuries (n=3) and those with peripheral duct injuries (n=10). There was no significant difference in volume of bile leak or duration of leakage between the 2 groups. Bile leaks presented as peritonitis (n=6) or as a bile leak through a drain site (n=7). All but one closed spontaneously over a median of 33 days (range 3-110). There was no mortality but patients incurred a median of 4 complications (range 1-5). Both respiratory complications (8/13) and intra-abdominal sepsis (7/13) were more common in this group of patients than in patients with liver injuries not complicated by bile leakage (84/293 respiratory complications and 20/293 intra-abdominal sepsis; Fisher exact test, p=0.0164 and 0.0001 respectively).

In conclusion 2 patterns of injury were identified. Bile leaks were usually self limiting and whilst associated with significant morbidity were not associated with mortality.

P0047 CYSTIC LIVER LESIONS

J. Ham, M. Hollands, P. Douglas, K.P. Wong, R. Phillips, T. Wilson, J.M. Little.
Westmead Hospital, Sydney, Australia.

With widespread use of organ imaging an increasing number of patients with cystic liver disease are being identified. The aim of this study was to highlight the problems associated with making a diagnosis in these patients.

42 patients presenting with cystic lesions of the liver to either of 2 surgical hepatobiliary units over an 8 year period were reviewed retrospectively. Patients with "clear cut" hydatid disease (n=63) were excluded. In 26/42 the initial radiological findings were re-reported "blind" by a radiologist from another hospital and a presumptive diagnosis reached. The presence or absence of certain radiological characteristics was also noted and the results subjected to multiple regression analysis. A final diagnosis was reached at operation.

A final diagnosis of simple cystic disease was made in only 13/42 patients. Of the remaining 29, 7 had unsuspected tumours, 3 had unsuspected hydatid disease and the remainder a multitude of other conditions. The initial radiological diagnosis was correct in 22/42 cases. 12 patients were incorrectly labelled as hydatid disease. "Blind" radiological review led to an accurate diagnosis in 15/26 patients. The radiological features suggestive of malignancy were a solid component to the cyst wall and rim enhancement on contrast CT.

This series demonstrates the difficulties encountered when trying to diagnose cystic lesions within the liver and there is room for a healthy scepticism when the diagnosis is suggested by a radiologist. Even apparently simple cysts may be tumours or hydatids. A prospective study now underway has confirmed the difficulties identified in this review.

### P0048 ACUTE PANCREATITIS. OUR EXPERIENCE IN THE LAST TEN YEARS.

F Santolaria, CE González-Reimers,
P Machado, N Batista, H Essardas,
A Alarcó, J Pérez Palma.
Hospital Universitario de Canarias.
La Laguna, Tenerife, Canary Islands.
SPAIN.

Acute pancreatitis is a potentially fatal disease. Some clinical and biological features at admission may have prognostic value. In the present study we have analyzed the clinical and biological features of 274 patients (146 males and 128 females) with acute pancreatitis admitted to our medical unit during the last ten years. Nineteen patients died (mean age 55.1±3.8 years), whereas 255 survived (mean age 50±1.1 years). Mortality was highest among the idiopathic pancreatitis (28.5%) and lowest among the biliary ones (4.8%). Highly significant associations were found between alcoholic etiology and male sex, and biliary etiology and female sex ($p<0.0001$). Biliary pancreatitis showed significantly higher values of ASAT, ALAT, GGT, alkaline phosphatase, bilirubin, cholesterol and serum proteins than alcoholic pancreatitis. Patients who died showed higher BUN, glycemia, LDH and creatinine, and lower hematocrit, serum proteins, serum albumin, serum calcium, serum cholesterol, prothrombin activity, and $PaO_2$. Fifty four patients showed at least one of the following criteria of severity: shock, sepsis, acute respiratory distress syndrome (ARDS), abscess, pseudocyst or acute renal failure. Among the severe cases mortality reached 35%; no one of the patients with ARDS survived.

By multivariate analysis, the following seven parameters at admission independently correlated with mortality: hematocrit < 35%, serum proteins < 60 g/l, serum creatinine > 2 mg/dl, LDH > 450 U/l, body temperature > 38°C, BUN > 30 mg/dl and prothrombin activity < 80%. Mortality rate was only 1.2% when 1 parameter was present, 6.6% when 2 or 3, and 61% when four or more parameters were present.

**P0049** TREATMENT OF ADVANCED PANCREATIC CANCER WITH LH-RH ANALOGUE

C. Sperti, C. Pasquali, S. Catalini, C. Militello, A. Piccoli*, S. Pedrazzoli., Clinica Chirurgica 1 & Medicina Interna*,University of Padua, Italy.

Recenly estrogen and androgen receptors have been demonstrated in normal and malignant pancreatic tissue. It was therefore proposed that endocrine manipulation may be valuable in the treatment of this malignancy. Case reports of objective responses to LH-RH analogues have also been published (Lancet, 1986).In this study we report the results of the treatment of unresectable pancreatic cancer with a LH-RH analogue, goserelin. 33 patients with histologically proven pancreatic adenocarcinoma entered in the study and located at random in 2 groups: group A (15 patients) treated with goserelin (zoladex, ICI Pharma, Milan, Italy) 3.6 mg s.c. every 4 weeks and group B of 18 untreated patients. Patients were reviewed monthly for clinical examination, routine laboratory tests, Ca19-9 serum levels, and every 3 months chest x-ray, abdominal U.S. and /or CT were performed. LH secretion was successfully suppresed by goserelin, and serum testosteron fell dramatically in males. Survival time from diagnosis was evaluted statistically in both groups by Mantel- Cox, Tarone and generalized Wilcoxon tests. No objective response to treatment was demonstrated, either complet or partial. No sustained improvement in performance status and decrease of Ca 19-9 serum levels were observed despite theraphy. 3 patients in group A and 3 in group B had stable disease; 2 patients in both groups survived > 1 year. Survival was not statistically different in 2 groups ($p = 0.21$). Median survival time was 7 months for group A and 4 months for group B. No toxicity due to LH -RH agonist administration was seen. This study suggest that LH-RH analogues are unlikely to have a major influence in survival of patients with pancreatic cancer.

Study supported by CNR grant. Project "Oncology" # 88-00804.44

**P0050** **EXPERIENCES WITH TWO DIFFERENT LITHOTRIPTORS FOR THE TREATMENT OF IMPACTED COMMON BILE DUCT STONES.**

Toom R den[1], Nijs HGT[1], Blankenstein van M[2], Schröder FH[3], Terpstra OT[1]. Depts. of [1]Gen Surgery, [2]Gastroenterol and [3]Urology, Univ Hosp Rotterdam (The Netherlands).

Extracorporeal shock wave lithotripsy (ESWL) is a new treatment modality for impacted common bile duct (CBD) stones. From April '86 - December '89, 49 patients with 1 or more CBD stones - for which endoscopic treatment failed - were treated with the Dornier HM-3 and the Siemens Lithostar. Visualisation was achieved by fluoroscopy. The stones were comparable in size in both groups (mean diameter of the largest stone 24 mm). Results:

| | Dornier | Siemens |
|---|---|---|
| Nr of patients (M/F) | 13 (1/12) | 36 (17/19) |
| Age (yr) (mean, range) | 78 (67-92) | 73 (27-95) |
| Shockwaves/patient | 2535 (1000-7000) | 7660 (2500-24850) |
| Sessions/patient | 1.3 | 1.8 |
| General anaesthesia | 13 (100%) | 1 (3%) |
| IV analgo-sedation | 0 (0%) | 31 (86%) |
| Fragmentation | 13 (100%) | 30 (83%) |
| Total clearance of CBD | 12 (92%) | 20 (56%) |
| Transient haematuria | 10 (77%) | 0 (0%) |
| Subcaps hemat R-kidney | 1 (8%) | 1 (3%) |

In the 6 patients where ESWL failed (Siemens), surgical choledochotomy was performed without complications. At follow-up (mean Dornier 31 months, Siemens 13 months), none of the patients had biliary complaints. In conclusion: ESWL for impacted CBD-stones is effective and safe.

**P0051** REGIONAL CHEMOTHERAPY FOR COLORECTAL LIVER METASTASES: AN EVALUATION OF TARGETED REGIONAL 5-FLUOROURACIL (5FU).

J.A. Goldberg, D.J. Kerr, N. Willmott,
C.S. McArdle.
Royal Infirmary & Strathclyde University,
Glasgow, Scotland, U.K.

The results of systemic chemotherapy in patients with liver metastases from colorectal cancer remain dismal.

Regional chemotherapy has been advocated as a method of improving the delivery of cytotoxic drugs to tumour, while minimising systemic toxicity. The use of vasoactive agents to redistribute arterial blood towards tumour, and biodegradable microspheres to slow tumour blood flow and enhance tumour drug uptake, have also been suggested as methods of further improving tumour exposure to drug.

We present 22 patients who received intra-hepatic arterial chemotherapy for colorectal liver metastases. Combined treatment (angiotensin II, albumin microspheres and 5FU) was administered 4 - 6 weekly, and bolus 5FU given in the intervening weeks.

Toxicity was minimal. Responses were seen in seven patients. Fewer than half the deaths were from liver metastases; a quarter of the patients died from non cancer-related causes. Survival was prolonged in the treated group, compared with historical controls.

These results suggest that this regime has activity in colorectal liver metastases and further studies of regional chemotherapy are warranted.

### P0052 MANAGEMENT OF LIVER ABSCESSES BY ULTRA-SOUND GUIDED ASPIRATION AND DRAINAGE

O. Shafey, F. Mekky, W. Shehab
Faculty of Medicine, Alexandria University
Egypt

Forty-five patients with intra-hepatic abscesses were diagnosed sonographically. The mean age was 33.5 years. Fourteen were females and thirty one were males. The main clinical presentations were right abdominal pain, fever, and tender hepatomegaly. Only five abscesses were in the left lobe, one in the caudate lobe and the rest in the right lobe. Diagnostic ultrasound guided aspiration was initially done and the aspirated fluids were subjected to bacteriological and cytological examinations.

It was proved by cytological examination to have amoebic abscesses with sterile culture. In the other thirty-five patients positive culture included eleven post-operative and two cholangioctatic abscesses. Staphylococcus aureus was the infective organism in eighteen patients and gram-negative in the rest. Patients with amoebic abscesses were managed successfully by guided aspiration combined with systemic anti-amoebic drugs. Among the pyogenic group, twenty-five patients were managed by multiple guided aspiration (up to three times) combined with systemic use of a specific antibiotic, eight with big or resistant abscesses were managed by ultrasound guided percutaneous catheter drain insertion with systemic and local specific administration. A complete cure was achieved in these patients with mean cure time of 23.3 days. One patient died from fulminating cholangitis. Another patient needed surgical drainage due to superficial left lobe abscess.

P0053 VASCULAR EXCLUSION IN HEPATIC RESECTION

A. Itoh, H. Taniguchi, A. Oguro, T. Daidoh, N. Tukuda, Y. Shioaki, T. Yamane, T. Yamaguchi, K. Sawai, O. Kojima, T. Takahashi
First Department of Surgery, Kyoto Prefectural University of Medicine, Kyoto, Japan

Among 40 patients who had undergone liver resections in the first department of surgery, Kyoto prefectural university of medicine hospital during recent one and a half years. Fourteen patients underwent liver resection without vascular exclusion (no exclusion group); 6 underwent clamping of either right or left hepatic artery and portal vein at hepatic resection (unilateral exclusion group); and 20 underwent hepatectomy with clamping of hepatic artery and portal vein at the hepatoduodenal ligament (total exclusion group). In these groups, some patients with liver cirrhosis were included but no significant differences among them. And there were no significant differences in disease, sex, age, preoperative liver function, and operative proceudre among them.

The operation time and the volume of blood loss had no significant differences. Serum lactic acid dehydrogenase (LDH), glutamic oxaloacetic transaminase (GOT), glutamic pyruvic transaminase (GPT), total bilirubin (T-B), and creatine phosphokinase (CPK) were measured on the first postoperative day. All postoperative laboratory data except for GOT and GPT were not significantly different among the three groups. GPT in the unilateral exclusion group was significantly lower than that in no exclusion group and total exclusion group by unpaired Student's t-testing ($P<0.025$, $P<0.01$). GOT in unilateral exclusion group was lower than that of total exclusion group ($p<0.005$).

These findings indicate that the liver is more damaged by complete portal and arterial exclusion than by unilateral exclusion at hepatic resection. Thus, unilateral portal and arterial clamping may be a better technique than complete clamping. When hepatectomy of bilateral liver lobes is performed, the exclusions of left and right sides should be done alternately.

### P0054 INCORPORATION OF BROMODEOXYURIDINE INTO VX2 METASTATIC LIVER TUMOR AFTER INTRAARTERIAL, INTRAPORTAL AND INTRAVENOUS ADMINISTRATION

Y. Shioaki, H. Taniguchi, A. Oguro,
A. Itoh, N. Tsukuda, T. Daidoh,
T. Yamaguchi, K. Sawai, T. Takahashi
First Department of Surgery, Kyoto
Prefectural University of Medicine,
Kyoto, Japan

To compare the pharmacotherapeutic effects of intra-arterial, intraportal, and intravenous administration of an anticancer drug for the metastatic liver tumor, bromodeoxyuridine (BrdU) was infused into rabbits with VX2 metastasizing to the liver from various routes.

Both bolus and continuous (30 min) injection of BrdU (10mg/kg) was performed from the celiac artery with or without ligation of the portal vein, from the portal vein with or without the hepatic artery, and from the vein of the ear. After the infusion, the liver was removed. The specimens were stained by the immuno-histochemical procedure by the avidin-biotin-peroxydase complex method using anti-BrdU monoclonal antibody.

The drug was incorporated into small metastatic liver tumors after both arterial and portal bolus injection. The higher uptake of BrdU in large tumors was seen after intraarterial infusion than after intraportal administration. However, even after continuous intra-arterial injection, BrdU was taken up only by peripheral cells of the large tumors. The intraportal and intravenous administration of BrdU without ligation of the hepatic artery had the same effect as intra-arterial injection.

In conclusion, it is speculated that infusion of anti-cancer agents acting on DNA systhesis is effective only for small liver metastases.

### P0055 EXPERIMENTAL STUDY OF THE DELIVERY OF BRDU-SALINE SOLUTION AND BRDU-LIPIODOL SUSPENSION TO METASTATIC LIVER TUMORS

T. Daidoh, H. Taniguchi, A. Oguro, N. Tsukuda, A. Itoh, Y. Shioaki, T. Yamaguchi, K. Sawai, T. Takahashi
First Department of Surgery, Kyoto Prefectural University of Medicine, Kyoto, Japan

As a model of an anticancer agent acting on DNA synthesizing phase, bromodeoxyuridine (BrdU) was injected into the hepatic artery of rabbits with liver metastases induced by VX2 carcinoma. We compared the pharmacotherapeutic effects of BrdU-saline solution and BrdU suspended in Lipiodol, a lipid contrast medium, on the metastatic liver tumor.

The liver was removed 24 or 72 hours after the injection, and specimens were stained using avidin-biotin-peroxidase complex method with anti-BrdU monoclonal antibody. Metastatic lesions were classified to four groups by their diameter ( large being more than 5 mm ) and extent of BrdU labeling ( strongly labeled indicating more than 50 % of the lesion being labeled ).

Twenty-four hours after the injection, 16.5 % and 1.2 % of the lesions were small with weak labeling when the solution and suspension, respectively, were used. Seventy-two hours after the infusion, they accounted for 3.6 % and 32.3 %, respectively. Twenty-four hours after the infusion, no large and strongly labeled lesions were observed when the solution was used, compared with 5.8 % when the suspension was used. Seventy-two hours after the infusion, the values 0 %, and 31.3 %, respectively.

It was concluded that Lipiodol enhanced drug delivery to the metastatic liver.

### P0056 OUR EXPERIENCE IN THE TREATMENT OF ACUTE BILIARY PANCREATITIS

I. Viyachki, P. Tokov, N. Yaramov,
T. Pozharliev, D. Viyachki
Medical Academy, Sofia, Bulgaria

The authors have studied 207 cases of acute biliary pancreatitis, among them 80 men and 127 women. In all cases in the complex medical therapy the following was included: high dosage of Trasylol, Ftorafur by scheme, forced diuresis, aqua-physiological and protein solutions, depending on the ionogramme, proteinogramme, the central venous pressure, diuresis, as well as the cessation of the pancreatic pain.

In 103 of all cases endoscopic papillosphincterotomy was done. From all the patients only 65 were operated on and in 5 of them splanchnicectomy was performed.

The authors propose practical conclusions on the basis of the analysis of the cases.

### P0057 EFFECT OF ADMINISTRATION OF PLASMA FIBRONECTIN ON LIVER REGENERATION FOLLOWING HEPATECTOMY IN RATS

A. Kwon, S. Uetsuji, M. Yamamura, Y. Kise, K. Hioki, M. Yamamoto
Department of Surgery, Kansai Medical university, Osaka, Japan.

Following 70% hepatectomy, male Sprague-Dawley strain rats were given purified human plasma fibronectin (FN) and/or aprotinin intraperitoneally after hepatectomy and were divided into 4 groups: Group FA (40 mg/kg of FN and 25,000 KIU/kg of aprotinin), Group F (40 mg/kg of fibronectin), Group A (25,000 KIU/kg of aprotinin), and Group C which served as a control. The liver regeneration rate 72 hours after hepatectomy in Groups FA and F, and the plasma FN levels at 24 and 72 hours following hepatectomy in groups with FN, were significantly higher than those of the control group. The incorporation of $^3$H-thymidine into DNA and the phagocytic index of the reticuloendothelial system at 24 hours in Groups FA and F, and the mitotic index at 72 hours after hepatectomy in Group FA were significantly increased as compared to the control. But the supplementation of 20 mg/kg of indomethacin to the 4 groups resulted in significant suppression of $^3$H-thymidine uptake 24 hours after hepatectomy. Moreover, plasma fibronectin levels in rats correlated well with the phagocytic index and DNA synthesis 24 hours following partial hepatectomy. Prostaglandin $E_2$ level at 3 hours after hepatectomy in Group FA was higher than the control. These results suggest that fibronectin may act as an activator of the reticuloendothelial system, related to prostaglandins, especially $PGE_2$, in the process of liver regeneration. And, then, aprotinin may enhance the effect of FN by inhibiting the proteolytic degradation of FN.

### P0058 IMPROVING TECHNIQUES IN SURGERY OF HEPATIC HYDATIDOSIS

C. Vagianos, D.Karavias, N. Bouboulis, M. Stavropoulos, J. Androulakis.

Department of Surgery, University of Patras, Patras, Greece

Despite hygienic measures, hydatidosis retains its high prevalence in endemic areas. Liver is involved in 75% of cases, and even if asymptomatic, surgical treatment is required, as complications will inevitably ensue. Postoperative morbidity relates mainly to the residual cavity and involves abscesses and biliary fistulae.

Sixty four patients were operated on for hepatic hydatidosis, over a period of seven years (1982-1988). Main clinical manifestations were epigastric pain (84%), hepatomegaly (31%), and history of fever (30%) or jaudice (25%). Diagnosis was established by ultrasonography and computerized tomography (sensitivities 95% and 93%).

In five patients (8%) total cystectomy was performed without postoperative morbidity. Nineteen (30%) were treated by limited unroofing and evacuation of the cyst, omentoplastry and suturing of bile ducts found in the residual cavity. Morbidity reached 30%. Fourty patients (62%) were subjected to wide unroofing and evacuation of the cyst, over and over suturing of the edges, with or without omentoplastry. No bile ducts were sutured in the residual cavity that was drained by a high vacuum drain. Postoperative morbidity diminished to 2.5%.

There was no mortality.

Mebendazole was administered selectively. Hypertonic saline was used as scolicocidal.

We consider the last surgical technique the treatment of choice in hepatic hypatidosis. It results in a shallow cavity, avoiding pooling of secretions, while the high vacuum drain removes them, and possibly, by expanding the cavitary wall, it obliterates biliary ducts. Omentoplastry can as well be avoided as it does not seem to further improve the results.

**P0059** **THE pH VALUES OF BILE SAMPLES IN BILIARY TRACT DISEASES**

*Rasih YILMAZ, Mehmet TUĞCU, Candeğer YILMAZ*

*Ülkü BAYINDIR, Orhan ÖZBAL*

*Ege U.Medical School General Surgery and Internal Medicine Dept's Izmir/TURKEY*

*In this clinical study,the pH changes in samples of gallbladder and common duct biles were determined in various biliary tract diseases.Bile samples were provided from gallbladder and common duct by puncture during laparotomy.The pH,pO2,pCO2,$HCO3^-$ levels were determined.*

*Results,*

*Gallbladder bile samples*

| No. | Disease | pH | p02 | pCO2 | $HCO3^-$ | p |
|---|---|---|---|---|---|---|
| 7 | 1 | 7.37±0.44 | 96.2±8.1 | 25.2±4.6 | 17.3±4.1 | |
| 59 | 2 | 7.10±0.86 | 85.2±7.7 | 46.5±5,3 | 14.3±3.6 | 0,05 |
| 7 | 3 | 6.99±0.26 | 73.0±8.8 | 59.2±4.9 | 18.9±4.1 | 0.o1 |
| 16 | 4 | 7.22±0.90 | 88.5±3.7 | 51.0±2.6 | 18.8±1.9 | 0.05 |
| 9 | 5 | 7.30±0.78 | 90.5±9.0 | 35.3±6.8 | 16.2±3.2 | 0.05 |

*Common duct bile samples*

| No. | Disease | pH | p02 | pCO2 | $HCO3^-$ | p |
|---|---|---|---|---|---|---|
| 7 | 1 | 8.53±0.33 | 115.9±10.9 | 14.1±3.2 | 216.7±31.1 | |
| 13 | 2 | 7.98±0.87 | 110.9±16.0 | 19.7±3.2 | 226.2±39.0 | |
| 16 | 4 | 7.85±0.68 | 93.2±11.1 | 22.9±4.2 | 128.2±23.1 | |
| 9 | 5 | 8.12±0.26 | 110.2±20.1 | 15.9±3.5 | - | |

*The pH of bile varies in a constant range(6.7-7.4) in healty persons.The pH is decreased significantly in biliary tract infections in tnis series.E.coli (6/7) is main microorganism and acid production of this microorganism is responsible from decline of pH.The significant decline of p02 in acute cholecystitis group may be explained with excessive consumption of 02 by aerob microorganism.*

*Similar findings are present mildly in chronic cholecysititisThe significant differences between values of gallbladder and common duct samples are explained with concentration gradients between bile samples.There is need to need to more research for explanation of change in common duct bile samples.*

*(*) 1.No biliary disease 2.Chronic calculous cholecystitis 3.Acute cholecystitis 4.Choledocholithiasis 4.Malignancy*

**P0060** HEPATIC RESECTION FOR SOLID LESIONS - IS PREOPERATIVE BIOPSY NECESSARY ?

J.Pain, E.Howard
King's College Hospital, London, UK

The decision to resect an hepatic lesion is commonly based on symptoms, anatomy and biopsy histology. Having experienced three needle tract tumour recurrences after biopsy we reviewed the results of preoperative investigations in a consecutive series of 69 patients (including 25 children) who underwent hepatic resection for solid lesions.

Thirty three had primary tumours, seven secondary tumours and 29 benign lesions. Preoperative ultrasound was performed in all patients, 64 had hepatic angiography and 56 had CT scans. Seven patients had undergone an open biopsy at a previous laparotomy and 12 had undergone a percutaneous 'Trucut' biopsy.

The preoperative diagnosis based on clinical, biochemical (including serum alpha fetoprotein) and radiological findings had been correct in 64 of the 69 patients (accuracy 93%). The benign or malignant nature of the lesion was incorrect in only two cases (accuracy 97%). The reported histology of three of the seven open biopsies and two out of the 12 'Trucut' biopsies differed from that of the resected specimen (accuracy 74%, accuracy of benign or malignant nature 89%). Most of the biopsies had been performed and reported in hospitals that did not have a specialist interest in hepatology. The object of the study was not to assess its accuracy, but rather to assess its need in patient management.

Preoperative clinical, biochemical and radiological findings provide an accurate prediction of the histology. The decision for surgery is rarely governed by histology, but rather symptoms, extra- or intrahepatic spread and size of lesion. Biopsy should only be considered if there is doubt over the interpretation of other investigations and its result is likely to alter the decision whether to operate.

### P0061 GASTRIC ACID AND PEPSIN SECRETION IN PATIENTS WITH PANCREATIC CARCINOMA

Z.Puchalski,J.Snarska,H.Dzienis

Department of General Surgery Medical Academy Białystok,Poland

Many reports show considerable connections between the stomach and pancreas functions which also occur at disease conditions of the organs.The aim of present work was to evaluate a peptic action of the stomach in 35 patients operated on because of carcinoma of the pancreas.The patients were divided into 2 groups dependent on an advancement of the neoplasm process.First group consisted of 10 cases undergoing a complete resection of the tumour and the second group was 25 patients with inoperable pancreatic cancer.Peptic activity of gastric juice and quantitative output of pepsin were determined as well as basal and pentagastrin stimulated acid secretion.In the I group of patients,gastric acid secretion and peptic activities at basal and stimulation conditions were in normal range,while acid and pepsin values of the II group were significantly decreased as compared with results of healthy subjects.The results obtained in cases of inoperable pancreatic carcinoma showed a diminished secretory function of the gastric mucosa related to chronic gastritis that was found in histological examinations and mighat suggest a decreased response of the glandular cells to stimulation in advanced neoplasms of the pancreas.

### P0062 SURGICAL MANAGEMENT OF THE RARE BILIOBILIARY FISTULA

P.Peveretos, B.Patzis, M.Glinatsis, C.Demopoulos, G.Bonatsos, B.Golematis

First Dept. of Propaedeutic Surgery, Athens University, Greece.

Biliobiliary fistula is a rare complication of longstanding cholelithiasis with recurrent bouts of acute cholecystitis. This fistula usually occurs as a result of pressure necrosis from stones lying within an inflammed gallbladder on the adjacent common bile duct.

Although it is important to recognize this condition pre-operatively, so the proper operative strategy can be planned in advance, in many cases it is an intraoperative finding. Biliobiliary fistula poses difficult technical problems due to altered anatomy and dense inflammatory fibrosis. In such a case, any attempt for a classical cholecystectomy poses grave risk to the integrity of the upper extrahepatic biliary tract. Persistent dissection around Calot's triangle may lead to inadvertent bile duct injury. During the last ten years we have experienced with 14 patients with biliobiliary fistula. The clinical presentation was mostly unhelpful in the correct diagnosis, while ultrasound findings were inconclusive. Direct intraoperative cholangiography through the gallbladder was the mainstray of diagnosis. Main steps in our operative management of this condition included:

1. Cholecystotomy, stone removal and exploration of the common duct through the defect.
2. Retrograde partial cholecystectomy leaving a cuff of gallbladder for closure of the defect around a T-tube.

One patient died postoperatively from cardiovascular and infectious complications. In the follow up period, one patient presented with bile duct stenosis, while in the remaining twelve patients an excellent long-term result was obtained.

**P0063** REOPERATIONS ON BILIARY TREE.

Viti G., Casadei R., Greco V.M., Piva P.,
Marrano D.
Istituto di 1^ Clinica Chirurgica della
Università degli Studi di Bologna (Italy)

In our Institute, 2150 operations for primitive benign lesions of biliary tree have been performed in the period between 1975 to 1989. Reoperations on biliary tract have been performed in 117 cases: particularly the remaining cystic stump, containing stones, has been removed in 13 cases; choledocotomy for residual or relapsing stones in the biliary tract, has been performed in 53 cases. 41 operations have been performed for stenosis of the common bile duct following the prior surgical operations with or without associated lithiasis. An hepatocholedochus plastic has been performed in 11 cases; a bilio digestive anastomosis in 27 cases (an hepaticojejunostomy in y-en--Roux loop has been preferred in 14 cases). Moreover 3 hepatic re-sections have been performed: an infected intrahepatic lithiasis located before a stenosis was the indication in 2 cases, and an echinococcosis draining in the left hepatic duct, in a patient already operated for cholecystectomy and choledochotomy was the cause of the remaining liver resection.
26 patients had been operated upon previously only once on biliary tract, 13 twice, 2 thrice and in one case the patient had been operated upon four times.
We have recorded only one post-operative death (2,4%): he was an over 80 years old man whose operation was a left hepatectomy for miltiple abscesses of the left part of the liver.
The long-term results have been: good inthe 85% of the cases, moderate (dyspeptic manifestations, recurrent cholangitis) in the 12,1% of the cases, bad (need of a reoperation ) in one case.

### P0064 SURGICAL APPROACH TO SEGMENT I - LESIONS

J. Scheele, R. Stangl

Department of Surgery, University Hospital, Erlangen, FRG

Surgical access to the caudate lobe and process is difficult because of the concealed anatomical position between the hilum and the vena cava, and the somewhat obscure boundaries anteriorly and to the right (Mizimoto and Suzuki 1988).

The isolated monosegmentectomy I is suitable for small lesions in the left half of the caudate lobe, in particular if they are benign (Bismuth et al. 1982). Tumours located in front of the vena cava in the caudate process require an individualized approach, ranging from central hepatic resection to extended left or right hepatectomies and lobectomies, respectively (Scheele 1989). Occasionally parts of vena cava or hilar structures need to be removed as well.

These technical aspects, and the results obtained, are illustrated in a series of 18 patients in whom segment I was involved either isolated (n=11) or as part of a multifocal disease (n=7). Three patients underwent a monosegmentectomy I, four had various poli-segmentectomies, whereas in the remaining 11 standard resections were performed (extended left hepatectomy 3, left hepatectomy 2, right hepatectomy 3, and right lobectomy 3). A patch of the vena cava was removed in one patient, and the entire retrohepatic segment in two, followed by interposition of a Gore-tex prosthesis. In two additional cases, tumour extension necessitated to excise the terminal portion the remaining left and right hepatic vein, respectively, with subsequent reinsertion to the vena cava.

There was one operative death. At January 1, 1990, the five patients with benign conditions are alive and well. Following removal of malignant tumours, six patients died from cancer relapse, whereas two are alive with, and five without disease at 13 to 79 months.

References:
Mizimoto R, Suzuki H: World J Surg 1988; 12: 2-10
Bismuth H, Moussin D, Castaing D: Worl J Surg 1982; 6: 10-24
Scheele J: In Lygidakis NJ, Tytgat GNJ, Thieme 1989: 219-247

### P0065 ULTRASONOGRAPHIC INTRAOPERATIVE IMAGING OF THE PAPILLA OF VATER AND COMMON BILE DUCT

D Flati, G Flati, B Porowska, P Negro

A Trecca, C Zompetta, F Gaj, F Gossetti

M Carboni

Surgical Unit, Rome, Italy

In the present paper the AA. report their experience on routine intraoperative ultrasonography (I.O.U.) and cholangiography (I.O.C.) in pzs undergoing surgery for biliary tract lithiasis. Fifthy-eight (58) pzs were examined intraoperatively either with ultrasonography or with cholangriography. The former procedure was performed by the surgeon in a standardized fashion in order to scan cystic duct, confluence, "carrefour", left and right hepatic duct. CBD (walls and lumen), retroduodenal portion of CBD and papillary structures (ampulla, Santorini's valves, septum, confluence with Wirsung duct). Telecholangiography was then performed by radiologist who was not informed about the ultrasonographic findings. Two parameters were considered for statistical analisis: 1)size of CBD (not dilated < 8mm, dilated > 8mm), 2)size of stones (<1 mm, 1-3 mm, >3 mm). In ours series I.O.U. was shown to be significantly more accurate than I.O.C. in the presence of dilated CBD and stones < 1 mm ($p<0.01$). The latter procedure was found more advantageous for direct assessment of biliary flow, while I.O.U. allowed in most cases a good visualization of Wirsung duct,biliopancreatic confluence, Santorini's valves, CBD wall, obviously without any risk of radiation.

Results Tab. I: Imaging of papillary structures and biliary flow

| | I.O.U. | I.O.C. |
|---|---|---|
| *W. Duct | 55 | 12 (reflux) |
| *Bilioduodenal flow assessment | not possible | good |
| *Anatomical papillary structures | | |
| determination of type of confluence | 47 | 12 |
| septum | 34 | - |
| Santorini's valves | 41 | 23 |
| papillary stenosis | 14(1 false pos.) | 13 |
| *Post-sphincterotomy control | possible | - |

Results Tab. II: Overall diagnostic accuracy for lithiasis

| *Test | I.O.U. | I.O.C. |
|---|---|---|
| * Sensitivity | 92.3 | 76.9 |
| * Specificity | 97.6 | 95.2 |
| * Neg. predictive value | 97.6 | 93.0 |
| * Pos. predictive value | 92.3 | 83.3 |

The AA. conclude that presently I.O.U. should be highly considered as a routine procedure in biliary surgery provided that surgeon is familiary with ultrasound imaging.

P0066 COMBINATION OF ULTRASONOGRAPHY AND DIRECT CHOLANGIOGRAPHY IN SUSPECTED OBSTRUCTIVE JAUNDICE

P.Magistrelli, R.Coppola, R.Masetti,
A.Messia, A.Antinori, A.Picciocchi
Catholic University of Rome - Italy

Ultrasonography (US) is the first diagnostic step in cholestasis, but its sensitivity is low if used as a single method. Thus several combinations of exams have been proposed in the effort to achieve a precise preoperative diagnosis in patients with cholestatic jaundice.
The aim of this prospective study was to evaluate the advantages that direct cholangiography can add to US in the assessment of these patients.
In the 18-month period from November 1987 to April 1989, 144 consecutive patients (80 males, 64 females, mean age 52 years, range 15-99 years) with clinical and biochemical findings suggesting obstructive jaundice entered the study.
All patients were first evaluated by US; direct visualization of the bile ducts was then achieved either by ERCP (138 cases) or by PTC (6 cases).
On the basis of US findings alone, 130 patients were diagnosed to have an extrahepatic obstruction and 14 to have intrahepatic cholestasis.
Direct cholangiography confirmed the diagnosis in 92 cases (sensitivity of US: 63.9%) and modified it in 52.
In 12 of 14 cases suspected on US to have an intrahepatic cholestasis, cholangiography showed an obstructive lesion. In 6 of 130 cases suspected on US to have an obstructing lesion, cholangiography documented the hepatocellular nature of jaundice, avoiding an unnecessary laparotomy.
In the remaining 34 cases, cholangiography modified the previous diagnosis with regard to the site and etiology of the obstructing lesion, allowing to plan proper treatment. In conclusion, the increase in diagnostic accuracy achieved combining US with cholangiography seems to overstep the increased risks, costs and discomfort for the patient that cholangiographic procedures imply.

P0067 THE HYDATID DISEASES OF THE LIVER - SURGICAL TREATMENT

Alfaras P., Dedeilias P., Maganas D., Giannopoulos P.
1st Surgical Department, Evangelismos Hospital, Athens - Greece

Hydatid disease caused by Echinococcus Granulosus remains a major problem, especially in rural adult population.

In this paper, we present our experience from the surgical management of 38 cases of hydatid disease of the liver within a period of 4 years (1986-1989) and we discuss our results.

Our patients cohort is composed by 21 male and 17 female with an age range of 15-18 years, mean age 52,8 years. In 18 patients the hydatic cyst was solitary. In 10 patients there were multiple hydatic cysts while in the other 10 patients there were multiple cysts in liver and other abdominal organs.

Thirteen of these cases were uncomplicated and the rest were complicated (rupture into the biliary tree or infection). In the group of the 13 uncomplicated cases, 4 cysts were possible to be excised totally, atypical hepatectomy was performed to another 4 cases and in the rest 5 cases the cavity which had been left after evacuation of the cyst was treated by deroofing of the cyst and obliteration inserting stitches or omentoplasty. A drain was inserted in the subhepatic pouch. In the group of the 25 complicated cysts, 12 of them were ruptured into the biliary tree. These were treated by evacuation under running with stitches the bile, leaking radicles, cholocustectomy and exploration of common bile duct. The cavity was treated as in the uncomplicated group.

The 13 infected cysts were treated with evacuation of the cyst and drainage by 3 way Foley or penrose. In some cases omentoplasty was done.

We discuss these cases. A short review of the literature is made as well.

P0068 SUBPARIETAL ACCESS LOOP FOR HEPATICOJEJUNOSTOMY

K Ravindranath, M Roddie, A Adam, IS Benjamin
HPB Surgery & Diagnostic Radiology, RPMS,
Hammersmith Hospital, London, UK.

Following hepaticojejunostomy for benign or malignant strictures further biliary access may be required post-operatively for radiotherapy, stenting or intrahepatic stricture dilatation, or stone extraction, or later for treatment of recurrent strictures or tumour. Such access may be provided by fixation of the Roux loop to the peritoneum of the abdominal wall.

We have fashioned such access loops in 43 patients, 38 at hepaticojejunostomy, and five as a secondary procedure. Twenty-five patients had benign strictures, mostly iatrogenic, two sclerosing cholangitis, three choledochal cysts, eleven hilar cholangiocarcinoma or gallbladder carcinoma, and two distal bile duct tumours. A trans-anastomotic tube was left for postoperative cholangiography, and then removed unless immediate procedures were to be peformed. There were no serious complications.

Twenty-six biliary manipulations were performed in 13 patients, with failure to achieve the principal objective on six occasions. Procedures were performed immediately after operation in eight cases (iridium wire insertion in three, dilatation and stone extraction in five). There were nine delayed interventions (five dilatation and stone extraction, three cholangiography for suspected recurrent strictures, two with dilatation, one stent placement for tumour recurrence).

This procedure allows a wide range of biliary manipulations, and may avoid repeated transhepatic procedures in patients with chronic biliary disease. As a secondary procedure for chronic biliary access it is a safe alternative to reconstructive biliary surgery. We feel that this procedure is a useful routine addition to Roux-en-Y hepaticojejunostomy both in cases where further intervention is anticipated and as a safeguard to avoid future PTC if later cholangiography is indicated.

### PO069 PREDICTION OF POSTHEPATECTOMY LIVER DYSFUNCTION USING LIVER FUNCTION TESTS

Lamesch P., Ringe B., Oellerich M., Rabe C., Burdelski M., Weimann A., Gubernatis G., Pichlmayr R.

Careful preoperative evaluation of liver function is mandatory for prediction of postoperative outcome after liver resection, particularly in cirrhotic patients. Postoperative complications occur in 15% of the cases. In a retrospective study results of ICG and MEGX test (Monoethylglycinexylidid - a lidocaine metabolite) in patients undergoing hepatic resections were compared to routine biochemical parameters.

**Material & Methods:** 32 patients underwent 23 hemihepatectomies and 9 atypical resections. Preoperative routine biochemical parameters (transaminases,cholinesterase, bilirubine and prothrombime time) were compared to MEGX formation rate and indocyanine-green clearance. (ICG: 0,5 mg/kgBW, serum samples drawn 0,5,10,15,20 min. >i.v.inj.; MEGX: 1 mg/kgBW lidocaine, MEGX serum concentration measured 15 min. after i.v. inj.). The results were analysed with regard to the postoperative course (poc). A complicated poc was defined by persistent hyperbilirubinemia on the 7th postoperative day (pod) (>50µmol/l) and prolonged requirements of plasma substitutes (>1 fresh frozen plasma/day during 7 days). Results are indicated as median/range; for stat.analysis U-test was used.

**Results**: 28 patients experiencing an uneventful poc, all of them were discharged after 10-20 days, 4 patients had a complicated poc according to the criteria defined above (2 patients have died on the 7th and 20th pod).

| | MEGX [µg/l] | ICG [ml/min/kg] |
|---|---|---|
| uneventful poc (n=28) | 73(31-186) | 7,4 (2,3-15,8) |
| complicated poc (n=4) | 16 (7-49) | 6,3 (3,3-10,0) |
| | p<0,05 | n.s. |

Preoperative routine biochemical parameters showed no significant differences.

**Conclusion**: A complication rate of 12% matches the figures reported in the literature.The results give evidence that ICG and MEGX test may be valuable additional parameters for preoperative evaluation of liver function in patients undergoing hepatic resections.

P0070 TECHNIQUE AND INDICATIONS FOR USE OF THE PORTO-RENAL SHUNT IN THE TREATMENT OF VARICEAL HEMORRHAGE

W. David Lewis, M.D., Herminio Sanchez, M.D., Roger L. Jenkins, M.D., New England Deaconess and Lahey Clinic Medical Center, Boston, MA, USA

Although hepatic replacement has emerged as the most definitive treatment for chronic liver disease with variceal hemorrhage, a significant number of patients remain better served by portosystemic shunting. Historically, for those patients with coexisting ascites requiring side-to-side shunting, synthetic or autologous graft material has been interposed between the portal vein and inferior vena cava (IVC) when the two veins were not able to be brought into direct apposition. The need for two anastomoses and the failure rate from conduit thrombosis negatively influenced the desirability of using graft material.

The porto-renal shunt, described in 1964 but rarely used, requires a single anastomosis and employs only autogenous tissue. Indications for its use are based on anatomic difficulties encountered during portal decompressive procedures: when the portal vein and IVC cannot be brought into apposition because of fibrosis within the porta-hepatis, an enlarged caudate lobe, or a short portal vein segment.

The transected left renal vein, in continuity with the inferior vena cava, is anastomosed as a large caliber autologous conduit end-to-side to the portal vein. Using the renal vein in this manner allows the functional creation of a side-to-side portocaval shunt, without the use of synthetic or inferior caliber autologous vein patches.

Four patients who recently underwent porto-renal shunting are described to clarify indications for use of this technique and to describe the technical aspects of its construction. The use of magnetic resonance imaging in assessing shunt patency is also described.

P0071 EFFECTS OF CHLORPROMAZINE PRETREATMENT IN ISCHEMIC LIVER CELL INJURY IN THE RAT MODEL.

M. Locher, D. Henne-Bruns, J. Knop*, B. Kremer; Dep. of Surgery, * Radiology, Univ. of Hamburg, FRG

To evaluate, if chlorpromazine pretreatment could reduce warm ischemic liver cell injury (e.g. occlusion of the hepato-duodenal ligament during extended liver resec-tions), an experimental study was designed in the rat model.
The 2 ventral liver lobes of the animals (n = 50) were crossclamped for 3 h followed by a 1 or 24 h reperfusion interval. In half of the animals chlorpromazine (20 mg/kg body weight) was injected i.p. 30 minutes prior to crossclamping. In all animals a biliary catheter was introduced selectivly into the bile ducts of the crossclamped lobes. Biliary secretion, 99-Tc-HIDA secretion, wet and dry weight as well as intrahepatic distribution of 99m-Tc-labelled microspheres were determined and compared to untreated control animals as well as to values obtained from none ischemic liver lobes of the same animals.
Results: **Microcirculation**: After 3 h ischemia and 1 h reflow 1,9% of micropheres were retained in the ischemic liver lobes (untreated controls: 100%). After pretreatment with chlorpromazine this value increased up to 77 %. **Edema**: After 3 h ischemia and 1 h / 24 h reflow the wet weight increased up to 120 % / 115 % of untreated control animals. Pretreatment with chlorpromazine resulted after 1 / 24 h reflow in an increase up to 106 % / 110 %. **99-Tc-HIDA secretion**: After 3 h ischemia and 1 h reflow only 35 % were excreted. Pretreatment with chlorpromazine reduced the HIDA excretion to 2,3 %. After 3 h ischemia and 24 h reflow 38,3 % of HIDA was excreted by untreated control animals and 69 % in animals after chlorpromazine pretreatment.
These results show, that pretreatment with chlorpromazine reduces the postischemic edema as well as alterations of microcirculation although the substance itself induces a temporary inhibition of biliary excretion.

Keefe E et al. Gastroenterology 79: 222-231, 1980

### P0072 A NEW METHOD FOR EXPERIMENTAL HYPERTENSION TREATMENT.

S.P.Chikoteev,A.Y.Kim,O.A.Golberg.
Institute Surgery.Irkutsk.USSR.

Encephalopatia as one of the portacaval anastomosis complications aggravates postoperation results in I6,8% of pations.

A new operetive method in portal hypertension treatment is being suggested. This method is believed to prevent encephalopatia in the postoperetive period.

The first experimental results experience are reported in this paper. 7 mongrel dogs with the weight from 3 to I4 kg were taken. The method is create vascular communication between portal and caval systems. A distal end of the spleen artery was joined with the spleen vein by end-to-end anastomosis and another end-to-side anastomosis joined the spleen part of the spleen vein with vein of caval systems. The vascular stage was carried out by microsurgery help.
Thus, the portal blood circulation throufh the spleen to get into the lower caval vein system. Permeabilities of these anastomosis within 4 months were supported by angiography data.

Histological reseaches with the hematoxylin-eosin staining showed structural changes of the spleen vascular bed.

Works by A.Y.Potozkaya et al.(I973) and our first experimental results let us hope for making good progress.

<u>References:</u>

A.Y.Potozkaya et. al. Archives of pathology,35,p27-33.

### P0073 COMPARISON OF PRE- AND INTRAOPERATIVE DIAGNOSES IN FOCAL LIVER DISEASES

S.Karácsonyi, A.Petri, K.Kalmár Nagy

Department of Surgery, Albert Szent-Györgyi Medical University, Szeged, Hungary

During the last seven years (1982-1989) at the Albert Szent-Györgyi Medical University in Szeged we have treated 259 patients suffering from focal liver diseases.

The diagnosis on the basis of surgical intervention and histological results for the 259 patients are as follows: 9 abscesses, 79 liver cysts, 57 benign and 54 malignant liver tumors and 54 metastatic tumors. In six cases we found liver pathological changes arising from other illnesses (e.g. generalized endocrinopathy, gastro-duodenal ulcer etc.).

We compared the preoperative and intraoperative diagnostic results and than we tried to analyse the efficiency of different diagnostic methods.

The diagnostic value of the various iconographical methods is as follows: ultrasound 87%, isotopic scintigraphy 91%, computer tomography 80%, and angiography 83%.

### P0074 CHOLECYSTECTOMY IN ELDERLY PATIENTS. COMPARATIVE STUDY BETWEEN PATIENTS OVER AND UNDER 70 YEARS OLD.

J.M. Jover; J.M. Fradejas; J.C. Ruiz de Adana; D. Martinez; L de Benito; M.Moreno

Hospital Central Cruz Roja. Madrid. Spain.

The proportion of elderly persons in our population has been steadily increasing. Biliary tract disease is the most common problem that requires surgical treatment in this age group. The aim of this study has been to study mortality and morbidity following elective cholecystectomy surgery in four groups of patients, depending of the age and sex.

PATIENTS. From January 1986 to January 1989, 231 patients with cholelithiasishave been operated on in our Department divided in four groups:

- 168 patients under 70 years old: 115 females
  53 males
- 63 patients over 70 years old: 48 females.
  15 males

Most of the patients were on treatment for coexisting diseases: 60 females < 70 y. (52.1%); 37 males < 70 y. (69.8%); 39 females > 70 y. (81.2%), this parameter have reached statistical signification; and 10 males > 70 y. (66.6%).

RESULTS. Cholecystectomy without common duct exploration was performed in 95 females < 70 y. (82.6%); 46 males < y. (86.7%); 33 females > y. (68.7); and 12 males > 70 y. (80%).

Operative complications appeared in 11 females < 70 y. (9.5%); 10 males < 70 y. (18.8%); 8 females > 70 y. (16.6%); and 3 males >70 y. (20%).

The average hospital stay was 5,5 days in females < 70 y.; 6.8 in males < 70 y.; 8,8 in females > 70 y. ; and 12.0 in males > 70 y.

The mortality was 0% in all groups, except in over 70 years old females group that a patient died (2%). The global mortality was 0,4%.

CONCLUSION.

Cholecystectomy is often performed in elderly patients. The mortality in dective cholecystectomy in elderly is similar to the young patients. Early elective cholecystectomy in the geriatric patient before the development of acute complications should be considered unless significant medical contraindications to operation are present.

**P0075** RIGHT HEPATIC LOBECTOMY IN AN OLD PATIENT (HEPATOCARCINOMA + POLICYSTIC DISEASE + LIPOMA)

M. Moreno Azcoita, J. M. Jover, J. M. Fradejas, J. L. Ramos, J.C. Ruiz de Adana, L. De Benito. Hospital Central Cruz Roja, Madrid, Spain

The case of an 80 year-old male patient (Hbsg+) in good general state and with active life is presented. The patient was studied because of discomfort in right hypocondrium and several hepatic tumours were discovered in ultrasonography.

In the study of these tumours, policystic liver disease and hepatocarcinoma in a noncirrhotic liver by means of fine needle guided puncture-biopsy was diagnosed.

After the study had been completed and the risk evaluated, it was decided to submit the patient to surgery. Right hepatic lobectomy was performed.

The complete study of the specimen showed the existence of policystic liver with an hepatocarcinoma in the liver segments V-VI, and a big sized lipoma in liver segment VII.

The postoperative course was uneventful and at present, 8 months after surgery, the patient remains asymptomatic.

The reason to communicate this case-report is to present this rare association of hepatic tumorations in an elderly patient. At present we have not found any other similar case in the literature.

### P0076 BILIARY SURGERY IN LIVER CIRRHOTIC PATIENTS

T.J. Liu, C.C. Wu, T.C. Wu
Taichung VGH, Taiwan, R.O.C.

Biliary operation onliver cirrhotic patients has a very high incidence of morbidity and mortality. During the past 6 years, a total of 87 patients with varied degrees of liver cirrhosis received definitive biliary surgery in Taichung VGH. They were 64 males and 23 females with age ranging from 41 to 83 years. Thirty-nine patients fell in to the category of Child A liver function. 14 in Child B and 34 in Child C. Sixty cases underwent regular biliary surgery and the remainders received emergent operations for either empyema of gallbladder or obstructive cholangitis. Four patients with Child C cirrhotic liver received emergent operation but died of liver failure eventually. The operative efficacy was evaluated by the amount of blood loss during operation, hospitalization day, morbidity and mortality. Emergent biliary operation did not aggravated the liver cirrhosis in the patients with Child A and B liver cirrhosis. We concluded that emergent biliary surgery is recommendable and safe on Child A and B liver cirrhosis patients but not on the Child C cases.

PO077 PARENCHYMAL DAMAGE AND METABOLIC FUNCTION OF LIVER GRAFTS PRESERVED WITH EUROCOLLINS AND UW-SOLUTION.

J Pruim, IJ Klompmaker, R Verwer, MJH Slooff;
Liver Transplant Group Groningen, the Netherlands.

To study the effect of EuroCollins (EC) and University of Wisconsin -solution (UW) on liver grafts in the early recirculation phase of liver transplantation we compared a group of 11 grafts flushed with EC and a group of 12 grafts flushed with UW.
*Methods:* Blood samples were drawn at 10 min. before (basal values), and 5, 15, 30, 60, and 120 min. after declamping of the portal vein. Parenchymal damage was assessed by the AP, LDH, AST, ALT, and GGT; metabolic function by the serum bile acids and the plasma amino acids [(valine+leucine+isoleucine)/(phenylalanine+tyrosine)]-ratio (Morgan MY. Gut 1978;19:1068). Recipient age, sex, and diagnosis did not differ between both groups. The cold ischemia time (CIT) of both groups differed significantly (EC 6.9$\pm$1.2hrs., UW 15.1$\pm$ 4.4hrs., $p=0.001$) as did the blood loss after recirculation (EC 10.4$\pm$5.7 liters, UW 5.0$\pm$3.2 liters, $p=0.04$). The statistical calculations on the samples were performed with a multiple analysis of variance (MANOVA). A value of $p<0.05$ was considered significant.
*Results:* In both groups LDH, AST, and ALT increased significantly from basal values. However, levels in the UW-group were significantly lower compared to the EC-group. The AP and GGT declined significantly from basal values with no difference between groups. The bile acids also declined significantly from basal values with no difference between groups. The calculated amino-acid ratio showed an immediate and significant increase after declamping with no difference between both groups, indicating a proportional increase of amino acid catabolism in the liver.
*Conclusion:* Liver grafts in both groups regained equal metabolic function immediately after recirculation, despite longer CIT in the UW-group. In addition, UW causes considerable less parenchymal damage compared to EC.

P0078 **RESULTS OF RESECTIONAL SURGERY IN 104 CONSECUTIVE PATIENTS WITH CARCINOMA OF THE PANCREATIC HEAD.**

J.H. Allema, D.J. van Leeuwen, O.M. van Delden, L.T. de Wit, P.C.M. Verbeek, N.J. Lygidakis, M.N. van der Heyde. Hepatopancreaticobiliary Unit, Academic Medical Center, Meibergdreef 9, 1105 AZ Amsterdam-NL.

**Aim:** What are the results of surgery for pancreatic head malignancy ?
**Patients:** We studied operative procedures, complications and survival in 104 consecutive patients scheduled for subtotal pancreatectomy for pancreatic head malignancy (1983-1986 retrospectively, 1987-1989 prospectively)
**Results:** In 79% of the patients subtotal pancreatectomy was performed, but 21% required total pancreatectomy. Portal vein resection was performed in 13%.Operative (30 d.) was 2%, but hospital mortality was 8%, mainly after total pancreatectomy. Hospital morbidity: 15% of the patients required relaparotomy, 39% had intra-abdominal infections. Average hospital stay was 40 days, median stay 30 ıays.
Histopathological findings and results:

| Pathology: | amp carcinoma | distal CBD ca | pancr ca |
|---|---|---|---|
| N | 34 pat | 34 pat | 36 pat |
| % radical (tumor free margins) | 79% | 59% | 19% |
| 2-years survival | 70% | 25% | 11% |
| 3-years survival | 50% | 11% | 9% |

Radicality of resection was associated with longer survival. Only 4% of the patients developed a (new) diabetes after subtotal pancreatectomy. Half of the subtotal pancreatectomy patients required enzyme suppletion (steathorrhoea, weight loss).
**Conclusion:** Feasibility of resectional surgery has definitely been established in the eighties. Major challenges for the nineties are reduction of tumour recurrence by adjuvant therapy and reduction of postoperative septic complications.

### P0079 IATROGENIC INJURY TO ABERRANT HEPATIC DUCTS

R. W. Strong
Princess Alexandra Hospital
Brisbane. Australia.

The confluence of the right and left hepatic ducts is always extrahepatic. The right hepatic duct has two major branches - anterior and posterior draining the appropriate segmental areas of the right lobe. In the majority of individuals these branches join to form the right hepatic duct or join the left hepatic duct separately. In a small percentage of individuals the right segmental ducts drain directly into the gallbladder, cystic duct or common duct. Rarely, hepatic ducts drain directly into the gallbladder. These "abnormalities" are referred to as anomalous or aberrant hepatic ducts.

Aberrant ducts arise by a modification in the developmental process. Normally, the proximal group of multiple hepatic ducts arising from the hepatic diverticulum coalesce to form the common hepatic duct while the remainder absorb. Failure of absorption of these primordial ducts leads to the development of aberrant hepatic ducts.

Ten cases of iatrogenic injury to aberrant hepatic ducts have been referred to the author for surgical repair. The series of transected aberrant ducts comprised - two cases of the common hepatic duct draining directly into the gallbladder; four cases of aberrant segmental, sectoral or right hepatic ducts; four cases of segmental or sectoral ducts together with the common duct.

The serious consequences of surgical trauma to the bile ducts is such that all surgeons performing cholecystectomy need to be aware of and to identify these variations.

### P0080 DOSE OF 5-FU WITH SEVERITY OF THE HEMORRHAGIC NECROTIZING PANCREATITIS

Z Tao
Surgical Department, Chongqing First Aid
Center, Chongqing, P R China

This is a report of 13 patients with acute hemorrhagic necrotizing pancreatitis. All patients fulfilled our criteria for diagnosis and gradation of the severity of the disease and excluded diagnosis of oedematous pancreatitis. This is a practical and reliable method which may play an important role in immediate diagnosis of the definitive site and character of pancreatitis.

Seven unselected patients have been treated by operation and 6 patients were without operation: both had intravenous administration of 5-Fu as adjuvant treatment.

In the operated group, 5-Fu 0.5/d was given for preoperative preparation: 4 of them had mild disease, and 2 died postoperatively, while 3 had severe disease, and all died after surgery (mortality 71%). Operative findings included gross haemorrhagic ascites and typical haemorrhagic necrosis of the pancreas, but the surface of the pancreas appeared dry as there was dry gangrene and a distinct edge between necrotic and normal tissues.

In the unoperated group, 2 patients with mild disease received 5-Fu 0.5/d and 4 with severe disease 5-Fu 1.0-2.5/d. All patients survived and no serious complications occurred. The total dose was 4.5g, 6.0g, 8.0g and 10.25g respectively.

Theoretically and in this clinical investigation, a high dose of 5-Fu not only reflected gradations in the severity of pancreatitis but also suggested a close relationship with the production and release of trypsin and pancreatic kallikrein, since 5-Fu inhibits RNA and DNA synthesis and blocks secretion by the pancreatic cell. In addition 5-Fu reduces overlow of pancreatic fluid, with relief of severe pain and promotion of dry gangrene which usually improves the condition of the infection.

P0081 CLINICAL STUDIES ON LYMPHATIC METASTASIS OF PANCREATIC HEAD CANCER TO PARA-AORTIC LYMPH NODES

H. Kobayashi, T. Nagakawa, N. Kadoya, Y. Nakano,
T. Nakamura, N. Ueda, K. Maeda, M. Kayahara, T. Ohta,
K. Ueno, I. Konishi, T. Matumoto and I. Miyazaki
Department of Surgery II, School of Medicine,
Kanazawa University, Kanazawa, Japan

It is necessary for improving surgical prognosis to perform extensive lymph node dissection in pancreatic head cancer, because it shows a tendency to occur lymphatic metastasis quickly. Especially para-aortic lymph nodes as well as the other are important resected portion , so that the recurrence is mainly at the local site or the liver. In order to establish an appropriate para-aortic lymph node dissection, thirty nine cases of pancreatic head cancer were reviewed and eight cases of pancreatoduodenal cancer were studied to investigate lymphatic extent in para-aortic lymph nodes by the intraoperative injection of activated carbon particles. The carbon's extent were observed microscopically. Para-aortic lymph nodes were classified on A, B1, B2 and C according to the direction from the rostral site to the caudal and on IVC-pre, latero, retro, Inter, Aor-pre, latero and retro according to the closs section. Six cases( 15.4% ) among thirty nine cases had the metastatic para-aortic lymph nodes. The metastaticlymph nodes existed mainly in B2 area and Inter or Aor-pre. The most site that the carbons flowed was compatible with that of metastatic lymph nodes.

It found that, at first, the dirction of the carbon's extent was toward the dorsal site of B2 area, especially under the level of the renal artery, than from the rostral to the caudal. Above mentioned facts show that lymph node dissection of the para-aortic region should be an en bloc resection to the dorsal site as well as from the rostral to the caudal.

### P0082 HYDATID CYST DISEASE: A series of 194 cases

E. Sözüer, A. Bilge,A Sağlam, Ö Yavuz. Resident

Erciyes University,Medical School,Kayseri,Turkey

Hydatid disease is a serious health problem in Turkey. The aim of this study is to give general knowledge about disease and its symptoms, signs, diagnosis, surgical techniques and complications.

194 patients with hydatid disease were admitted to Surgical Department of Erciyes University and Şişli Etfal Hospital (İstanbul) between 1978 and 1989 and rewieved retrospectively. 87(44.1%) patients were male and 107 (55.8 %) female. In the patients with abdominal hydatid cyst the most frequent symptoms were right upper abdominal pain (65.9%), feeling of weight abundance(34.8%). The most frequent signs were he patomegaly (43.7%) and palpabl mass(38.5%). 135 patients were examined with ultrasonography which has diagnostic walue of 93.4 %. Preoperative complications were intrabiliary rupture(4.1 %) and infection of cyst(8 %) and anaflactic schock. All patients were operated on by using various surgical techniques; partial cystectomy-omentoplasty(87), partial cystectomy-external drainage(55), marsupialization (25), partial cystectomy- capitonnage (10), introflexion (7), pericystectomy (5), hepatic resection(4) and only biliary drainage(1).

Main postoperative complications were wound infection(13 %),abscess formation (2.6 %)and biliary fistula(2.6 %). Total mortality rate was 2 % in this series.

The diagnosis of liver hydatid cyst is not a problem after having ultrasonography. It also gives opportunity to follow the patients postoperatively. There is still controvery about surgical techniques performed. In this study partial cystectomy- omentoplasty was found to be superior to the other techniques from the point of morbidity, duration of hospitalisation and drainage of abdominal cavity. It can be thougth omentum may absorb the fluid oozing in the residuel cavity.

P0083 **ASSESSMENT OF LIVER FUNCTION AFTER HEPATECTOMY WITHOUT BLOOD AND PLASMA TRANSFUSION.**

J. Belghiti, B. Suc, F. Fékété

Service de Chirurgie HI Beaujon 92118 Clichy France

Liver function after hepatic resection is difficult to evaluate. This might be due to the presence of underlying disease, postoperative complications and to the consequences of perioperative blood and plasma transfusion. The aim of this work was to assess postoperative liver function tests after hepatectomy without perioperative blood and plasma transfusion. In all patients chronic liver disease and postoperative complications were absent.

From 1987, 33 patients underwent hepatic resection for benign (n=26) and malign (n=7) tumors. Resection without transfusions accounted for respectively 11%, 19% and 38% of all liver resections performed in 1987, 1988 and 1989 in our department. Portal triad clamping was performed in all patients during 5 to 45 min (mean 22 ±11). There were 12 segmentectomies, 12 bisegmentectomies, one trisegmentectomy and 8 quadrisegmentectomies (right hepatectomy). The weight of the liver resected range from 50 g to 1440 g. Liver tests including prothrombin time, serum transaminases, bilirubin, gamma glutamyl transpeptidase (GGT) and albumin were determined on the first, third and seventh postoperative days. No blood or plasma transfusion were given during this period. Results were as follows.

| | Alanine Transaminase | Prothrombin Time | Bilirubin | GGT |
|---|---|---|---|---|
| Day 1 | 419±256 | 63% | 22±14 | 47±40 |
| Day 3 | 270±206 | 68% | 23±19 | 63±52 |
| Day 7 | 161±112 | 75% | 17±21 | 117±108 |

No significant change of serum albumin was observed after hepatic resection. On day 1, mean value of prothrombin time was 49% in patients with major resection (> 500 g) and 79% in patients with minor resection (< 100g); the difference is highly significant between the 2 groups (p< .01). On day 1, the increase of Alanine Transaminase ranged from 106 to 1110 UI/l and was significantly correlated with the duration of operative liver ischemia (r=.67).

Normal postoperative biochemical changes after liver resection could be summarized as follow: (a) postoperative increase of transaminases is correlated to the duration of operative liver ischemia; (b) postoperative decrease of prothrombin time is related to the extent of resection and; (c) all biochemical changes are transient during the first postoperative week except the increase of the GGT.

P0084 SURGERY OF LIVER METASTASES

E. Forni, F. Meriggi, G. Morone, D. De Previde Prato, A. Maconi, C. Roda
Clinica Chirurgica Generale, University of Pavia, IRCCS Policlinico San Matteo (Italy)

Hepatic resection is now widely accepted as a safe and effective treatment for metastatic colorectal tumors. Current interest concerns the selection of patients who are most likely to benefit from such a therapeutic approach. Over a 20-year period from 1970 to 1990, a total of 40 patients with hepatic metastases from colorectal cancer were treated with various types of liver resection at our Institution. There were 24 men and 16 women who ranged in age from 29 to 80 years. The metastases were solitary in 18 cases. Sixteen of 40 lesions were synchronous; 24 were metachronous. The median interval between resection of the primary tumor and the diagnosis of metastasis was 12 months. The primary tumors were all adenocarcinomas. Operation procedures included 2 right trisegmentectomy, 10 right lobectomies, 8 left lobectomies, 10 left lateral segmentectomies, and 10 nonanatomic wedge resections. The overall mortality rate was 3 per cent. The median hospital day after hepatic resection was 16 days. For all patients, median survival was 32 months, with estimated survival rates of 44%, 24% and 12% at 3,5 and 10 years respectively. Patients with four or more metastases had significantly poorer survival rates than those with a single or multiple up to three metastases ($p<0.01$). Patients with Dukes'B tumors had a median survival time of 120 months compared with a median survival time of 28 months for the patients with Dukes'C lesion ($p<0.05$). Those patients who had complete clearance of their metastases had a statistically longer survival than patients who had less than 1 cm of uninvolved liver between the tumor and the resection line ($p<0.05$).

**P0085** INTRAOPERATIVE RADIOTHERAPY IN PANCREATIC CARCINOMA.

J. González, F. Pardo, J. A.-Cienfuegos, E. Balén, C. Benito, JL. Hernández, J. Voltas, I. Azinovic, F. Calvo.

Departments of Surgery and Radiotherapy. Clínica Universitaria de Navarra. Pamplona. Spain.

Intraoperative radiotherapy (IORT) play an important role in the treatment of unresectable pancreatic cancer. This procedure allows to administer a high dose electron beam to the tumor during the laparotomy.
From September 1984 until August 1989 we have treated 25 patients with unresectable pancreatic carcinoma; none of them had hepatic metastases. All patients underwent the same surgical procedure: pancreatic tumor exposure, histological corroboration, a single dose of 20-25 Gy, biliary by-pass and gastroenteric-anastomosis. Radiotherapy was completed with external fractioned radiation during 4-5 weeks.
Patients age ranged between 22 and 85 years (m. 65) 16 patients were male and 9 female. 21 patients had continous intense abdominal pain. In ten patients the tumor size was greater than 5 cms. Tumor localization was the head of the pancreas in 18 cases, the body in six cases and the tail of the gland in one. We have had five complications related to IORT. Four cases of GI bleeding due to duodenitis and one biliar stricture. The medium survival time of the expired patients was 9 months. Currently 12 patients are alive with a follow up that range between 8 and 30 months.
The most outstanding clinical observation due to IORT was the inmediate decrease of the abdominal pain in 18 of the 21 that suffered from it.
IORT is an usefull terapeutic alternative in local control of pancreatic carcinoma, with important improving in symptomatology, mainly abdominal pain.

**P0086** PERCUTANEOUS-TRANSHEPATIC CHOLANGEAL CATHETERIZATION - ITS DIAGNOSTIC AND THERAPEUTIC USE -

N. Toyama, T. Suwa, and S. Hirakata
Omiya Red Cross Hospital, Yono, Japan

Percutaneous-transhepatic cholangeal catheterization (PTCC) is the diagnostic and therapeutic procedure, using a fine guidewire under fluoroscope, to puncture the intrahepatic bile duct of the patient and to indwell the catheter in it. PTCC was performed in 420 pa-from 1976 to 1989. The purposes of PTCC are, firstly, the biliary decompression and, secondly, the repeated examination injecting the contrast media from the catheter to obtain the accurate diagnosis. In addition, various therapeutic procedures such as cholangeal endoscopic lithotomy, LASER, etc., can be available through the catheter tract. The conventional method of percutaneous-transhepatic biliary drainage has been done in patients with obstructive jaundice showing the dilated bile duct. However, we think that PTCC should be performed in both icteric patients and non-icteric patients with elevation of alkaline-phosphatase and leucine-aminopeptidase which suggests the biliary obstruction. PTCC is the useful diagnostic and therapeutic technique for patients with biliary disorders, and the catheter can be inserted into the pre-dilated bile duct which is not detected clearly by ultrasonagraphy and computed tomography before the jaundice reveals clinically.

**P0087** NEGATIVE SODIUM BALANCE AND HIGH PLASMA CONCENTRATION OF NATRIURETIC PEPTIDE AFTER COMMON BILE DUCT LIGATION

L.M. Oms, J. Valverde, F. Martínez-Ródenas, J.J. Sancho
A. Sitges-Serra
Department of Surgery, Hospital del Mar,
Barcelona, Spain

Renal failure complicating obstructive jaundice is probably linked to a reduction of the extracellular water compartment (Martínez-Ródenas 1989). To elucidate the mechanism leading to volume depletion in obstructive jaundice we carried out two experiments using New Zealand male rabbits. We first studied a group of common bile duct ligated (OJ1, n=17) and another group of sham operated animals (SO1, n=14) for ten days. Water and sodium intake and balance were calculated and renal function was measured. Plasma Atrial Natriuretic Peptide (ANP) levels were measured in seven animals whereas urinary prostaglandin excretion (PGE2, PGI2, PGF2 alpha and TXA2) was determined in five of each group. In the second experiment, ANP, Aldosterone and Plasma Renin activity level were measured 24 and 72 hours after sham operation (SO2, n=6) or common bile duct ligation (OJ2, n=6). Water and sodium balances (11 vs 379 ml, $p<0.0001$ and -0.32 vs 7.78 mEq, $p<0.0001$) and creatine clearance (3 vs 11 ml/min, $p<0.0001$) were lower in group OJ1. ANP and PGI2 were increased in jaundiced rabbits (26 vs 11 fmol/ml, $p<0.02$ and 65 vs 15 pg/min, $p<0.01$) whereas PGE2, PGF2 alpha and Tx A2 did not show significant differences. Aldosterone and ANP plasma concentrations were increased 24 (13 vs 4 ng/100ml, $p<0.0001$ and 41 vs 10 fmol/ml, $p<0.0001$) and 72 hours (14 vs 3 ng/100ml, $p<0.0004$ and 26 vs 12 fmol/ml, $p<0.05$) after common bile duct ligation. Plasma Renin activity was not different at 24 hours but increased at 72 hours (11 vs 3 ng/100ml, $p<0.006$) in the OJ2 group. Volume depletion associated with obstructive jaundice appears to be secondary to a negative balance of water and sodium. This could be related to increased levels of ANP and occurs despite a high plasma Aldosterone concentration initially independent on Renin activity. Increased level of PGI2 should be understood as an attempt to improve glomerular filtration rate by enhancing renal blood flow.

References:

Martínez-Ródenas F, Oms L M, Carulla X, Br J Surg, 1989; 76: 461-464.

### P0088 VITAMIN E AND SELENIUM IN EXPERIMENTAL CHOLESTASIS

S. Singh, J. Chakraborty, M.E. Bailey.

University of Surrey and The Royal Surrey County Hospital, Guildford, UK.

The obligatory role of Bile Salts in Vitamin E absorption is well established (Forsgren 1969, Gallo-Torres 1970), however little work on Vitamin E metabolism in the presence of complete Biliary Obstruction exists. Vitamin E together with Selenium have an important role in immune response mechanisms, a deficiency producing impairment of cell mediated responses (Sheffy and Shultz 1979) and abnormal neutrophil chemotaxis (Sokol *et al* 1984). An impairment of phagocytic function has been reported in patients with obstructive jaundice (Bailey 1976, Pain *et al* 1987).

In order to investigate these factors in complete biliary obstruction 12 Wistar rats underwent bile duct ligation and division. The animals were sacrificed after three weeks. The serum levels of Vitamin E were determined by High Performance Liquid Chromatography using tocopherol acetate as an internal standard. Serum Selenium levels were assayed by Atomic Absorption Spectroscopy. Hepatic Glutathione Peroxidase activity was measured according to a previously described method (Lawrence and Burke 1976).

The serum Vitamin E levels in the cholestatic rats were significantly lower than the controls ($p<0.001$). There was a reduction in the serum selenium levels ($p<0.100$). The Glutathione Peroxidase activities were significantly altered ($p<0.001$).

The results of the study clearly demonstrate a reduction in serum Vitamin E levels, Glutathione peroxidase activity, together with a less significant fall in serum Selenium. The effect of this deficiency on reticuloendothelial function is currently under investigation. The results of this and a human study will be reported at the meeting.

**References**

Forsgren L Acta Chir Scand. 1969;suppl 399:1-29.

Gallo-Torres HE. Lipids 1970;5:379-384.

Sheffy B, Schultz RD, Fed. Proc. 1979;38:2139-2143.

Sokol RJ, Harris RE, Heubi JE. Hepatology (abst) 1984;4:1048.

Bailey ME. BJS 1976;63:774-778.

Pain JA, St. J. Collier D, Ritson A. Eur J Sur Res 1987;19:16-22.

Lawrence RA, Burke RF. Biochem Biophys Res Comm 1976;71:952-958.

### P0089 DONOR/RECIPIENT DISPARITY IN ORTHOTOPIC LIVER TRANSPLANTATION

M. Davenport, K.C. Tan
King's College Hospital, Denmark Hill,
London, UK

Current problems of donor/recipient size disparity in orthotopic liver transplantation will be illustrated with regard to 4 cases in the King's / Cambridge series occurring in a 12 month period.

Patient No.1 was an 18 month old with end-stage liver failure following a portoenterostomy as an infant for extrahepatic biliary atresia. Due to rapid deterioration in liver function the next available donor had to be used. This proved to be a 15yr old boy. At transplantation a formal right hepatectomy was performed to overcome the disparity. This patient is alive 12 mths later.

Patient No.2 was a 7 yr old girl with fulminant liver failure due to an idiosyncratic drug reaction. Urgent transplantation was performed using a donor liver from a 45 yr old man. To overcome disparity a donor right hepatectomy was performed as a bench procedure. An arterial donor graft had to be taken from infra-renal aorta to rearterialise the graft. There was an initial uncomplicated postoperative period with good graft function however she developed a graft v host reaction and died at 3 mts after transplant.

Patient No.3 was a 49 yr old man with end-stage cirrhosis and hepatoma. The donor was a 5 yr old boy. In order to overcome the problem of a short donor IVC the recipient IVC was left in situ during the hepatectomy. The donor suprahepatic IVC was anastomosed to a common right and middle hepatic vein and the infrahepatic IVC closed off. At 2 mths after surgery there is good graft function and rapid donor liver expansion.

Patient No. 4 was a 19 yr old man with fulminant liver failure due to NonA NonB hepatitis. Urgent transplantation was carried out from an 8 yr old child. At surgery the recipient was "filleted" from the failing liver and the small graft anastomosed as for No.3 There was no significant recipient hepatic artery thus the donor was anastomosed to aorta via iliac arterial grafts. At 10 days a recurrence of NonANonB hepatitis caused acute graft failure and a second transplant was performed. All vascular anastomoses were noted to be patent.

Conclusion: No donor liver need be wasted due to size disparity.

### P0090 HIGH MITOMYCIN C CONCENTRATIONS IN LIVER TUMOURS CAN BE ACHIEVED WITH ISOLATED LIVER PERFUSION IN RATS

A Marinelli, DHA Pons, PJK Kuppen, CJH van de Velde
University Hospital, Leiden, The Netherlands

Most anti-cancer agents show steep dose-response curves. In order to achieve higher drug levels in liver metastases while maintaining low plasma levels an isolated liver perfusion (ILP) technique was developed and compared with hepatic artery infusion (HAI). With mitomycin C (MMC) a toxicity study in tumour free WAG rats was followed by a pharmacokinetic study in WAG rats with CC531 (a syngeneic colorectal carcinoma) metastases in the liver. Survival, weight loss, white blood cell (WBC) count and blood chemistry were chosen as toxicity parameters. High performance liquid chromatography was used to measure MMC concentration in tumour tissue and plasma. For each experiment 5-7 rats were used per group. <u>Results</u>: The maximum tolerable dose (MTD) via HAI was 1.2 mg MMC/kg. HAI of 1.5 mg/kg resulted in a tri-phasic time weight curve (1. 10 days rapid weight loss; 2. 20 days stable weight; 3. second fall in weight till death) suggesting an acute and a delayed toxic effect. In ILP setting the MTD was 4.8 mg MMC/kg. Liver toxicity was fatal when higher doses of MMC were administered. The rats treated with the MTD in ILP had a small dip in weight and a transient increase in bilirubin, SGOT and SGPT levels. No dip in WBC count was seen. In the pharmacokinetic study, tumour tissue concentrations were significantly higher after ILP with 4.8 mg MMC/kg (3399 ng/g) than after ILP and HAI with 1.2 mg/kg (1328 and 750 ng/g). The plasma levels of MMC, however, were significantly lower in both ILP groups (1.2 mg/kg:22 ng/ml; 4.8 mg/kg:212 ng/ml) than in the HAI group (1.2 mg/kg:539 ng/ml). <u>Conclusion</u>: In WAG rats the MTD of MMC is 4 times higher in ILP than in HAI setting. With ILP a 5 times higher tumour tissue concentration could be achieved, while plasma levels remained significantly lower.

### P0091 SURGERY FOR ADVANCED CARCINOMA OF THE GALLBLADDER

Y. Sugiura, S. Shima and S. Tanaka
National Defense Medical College,
Tokorozawa, Japan

Surgery for advanced carcinoma of the gallbladder(GBC) which infiltrates both the liver and hepatoduodenal ligament is well known to be difficult. The surgical results were very poor. That should be reconfirmed at least by comparing to those in carcinoma of the hepatic duct junction(HDC).

For the last decade we have been performed simultaneous resection of the hepatic lobe and pancreatic head for 19 cases of advanced GBC, at the same time, mere hepatic lobectomy for 16 cases of HDC. The reason why simultaneous resection was necessary for GBC was high frequence of lymph-nodes metastases around the pancreatic head, 50% in this study.

The surgical results of GBC and HDC were compared in reference to operative mortality rate and cummulative survival rates including operative deaths. Operative mortality rate was 31.6% in GBC and 6.3% in HDC(N.S. by chi square test). Two-year survival rate was 21.1% in GBC and 60% in HDC. No suvival more than three years had been available in GBC while two have survived over five years in HDC ($p<0.05$ by generalized Wilcoxon test).

The surgical results of advanced GBC were much worse than those of HDC. However, the fact that four cases of GBC survived more than two years must encourage surgeons who are taking part in the treatment of GBC. We reflectingly ought to have acknowledged the difficulty of the extended procedure for GBC in the early time when we initiated it.

P0092 THE MALIGNANT EXTRAHEPATIC OBSTRUCTIVE JAUNDICE: COMPARATIVE RESULTS AFTER BILIODIGESTIVE ANASTOMOSES AND ENDOSCPIC AND TRANSHEPATIC CHOLANGIOPROTHESES IN 137 CASES

H.D. Rahn, G. Gamstätter, B. Budur, H. Weigand, H. Peters

Surgical Department, City Clinics, Wiesbaden, Germany

Among the palliative measures of malignant extrahepatic obstructive jaundice competitive treatment of biliodigestive anastomoses has occured through endoscopic and transhepatic cholangioprotheses.

Since 1984 transhepatic cholangioprotheses have been carried out on 69 patients. The lethal rate in the hospital amounted to 10 %. The high rate of complications (49 %) was mainly caused by a cholangitis during the first days, which was however not of long duration by antibiotic therapy. The average survival period was 5 months. In 30 cases of endoscopic implantation of cholangio-prothesis the lethal rate amounted 5,5 %. Most of the complications (77 %) were caused by occlusion of the cholangioprothesis, which however could be remeded by endoscopic flushing or changing of the prothesis. The average survival rate was 6,1 months.
In 38 cases of biliodigestive anastomosis the lethal rate in the hospital amounted 34 %. The rate of complications (15 %) was comparatively low. The mean survival rate was 6,4 months.

The decrease of bilirubin value to normal rates was able to be reduced most effectively and durably by biliodigestive anastomoses. Due to the high lethal rate in the hospital however it is essential, that this most successful measure is carried out only on specific patients in a good physical condition with an expected survival rate of more than six months.

P0093 LIVER INJURY IN 446 PATIENTS

JEJ Krige, J Terblanche

Department of Surgery,
University of Cape Town, Groote Schuur
Hospital and MRC Liver Research Centre,
Cape Town, South Africa

The management of 446 consecutive civilian adult patients with hepatic injuries treated between 1978 and 1987 at a trauma referral centre was evaluated. Two hundred and ninety-five patients (66%) had penetrating injuries (stab wounds: 204 patients (46%), gunshot: 91 patients (20%), 151 patients (34%) had blunt trauma). Seventeen patients (3.8%) died during resuscitation. Simple operative techniques controlled bleeding in 344 (80.2%) of 429 patients who underwent laparotomy of whom 8 (2.3%) died of head injuries. Complex liver injuries in 76 patients (17.7%) required major procedures including hepatotomy and intra-hepatic vessel ligation (6.5%) resectional debridement (7.2%) or sublobar or formal hepatic resection (4%). Twenty-five patients (5.8%) in addition, required peri-hepatic packing for haemostasis. Post-operative complications in 151 of 392 survivors (38.5%) was highest in the blunt trauma group (57.3%). Pulmonary complications, sepsis, fistulae and haemorrhage were the major causes of post-operative morbidity. Overall mortality was 12.1% (54 patients). Seventeen patients died before laparotomy (8: head injuries, 9: major liver parenchymal trauma including juxtahepatic venous injuries in 5 patients involving vena cava (3) hepatic veins (1) and both cava and hepatic veins (1)). Sixteen patients died in the operating theatre (12: exsanguination due to juxtahepatic venous injury 7, liver fracture 3, lumbar veins, aorta 1; 4: head injuries). Twenty-one died post-operatively (multi-organ failure: 7, head injury: 4, sepsis: 3). Eight of 17 patients who had lobar resections, died.

80% of liver injuries can be managed by simple surgical techniques. 20% of injuries are complex and require careful surgical strategy for control of haemorrhage. The major cause of death was pre-operative and intra-operative juxtahepatic venous bleeding.

**P0094** RESECTION OF HEPATOCELLULAR CARCINOMA IN CIRRHOTIC LIVER

C.MARGARIT,J. BALSELLS,,E. MURIO,JL.LAZARO
R. CHARCO I.DIAZ,J.BONNIN
Hospital G.Vall d'Hebron.Barcelona.Spain

In the last 3 years 112 cirrhotic patients were diagnosed of hepatocellular carcinoma in our Hospital. Twenty of them who fullfilled the criteria of localized tumor and good liver function, underwent a laparotomy with the aim of resecting the tumor.In 5 patients resection was considered not indicated,liver transplantation was performed in two cases and 3 patients had a catheter placed in the hepatic artery for chemoembolization.

16 hepatic resections for hepatocellular carcinoma were performed in fifteen patients,one was operated twice for tumor recurrence. Mean age of these patients was 62 + 8 years,12 were males and 3 females. Ethiology of liver cirrhosis was: alcoholic in 6, postnecrotic in 4, autoinmune in one and idiophatic in 4. Liver function was acording to Child-Pugh classification as A in 14 and B in 2.

An abdominal approach by means of a right subcostal incision was used in 14 cases, a midline laparotomy in one and a right thoracotomy in another patient who had multiple abdominal scars from previous laparotomies.For an adequate exploration of the tumor a complete mobilization of the liver,thorough palpation and ultrasound exploration were always done.Intraoperative ultrasound exam was essential to localize the tumor in two cases and useful to delimit the tumor and rule out satellites or other nodules in the rest.Mean tumor size was 4,5 + 3,4 cm..A limited resection was performed in 11 right lobe tumors, meanwhile one left hepatectomy, two left lateral segmentectomies and 2 limited resections were done for left lobe tumors.A Pringle's maneuver with a mean duration of 22 + 6 min.,was utilized 10 times to decrease bleeding.Hemostasis of the transection surface of theliver was achieved with a fibrin sealant (Tissucol) and sometimes collagen.

Operative mortality has been 12,5% (two cases),one from bleeding and coagulopathy and another from liver failure and broncoaspiration.Ascitis has been the most common complication.Follow up ranges from 2 to 30 months (mean 12.7 + 8) the patient operated twice died from tumor recurrence,an other in living with another hepatoma.Tumor recurrence rate is 15.3%.Overall survival rate is 80 %.

Conclusion: resection of hepatocellular carcinomas in cirrhotic liver is feasible in selected cases with acceptable morbidity and mortality.A close follow up is mandatory to detect tumor recurrence.

**P0095** ADENOCARCINOMA OF THE GALLBLADDER
A RETROSPECTIVE STUDY ON 354 CASES

M. Kracht, B.Descottes, and the
French Association for Surgical
Research (A.R.C., A.U.R.C.), Colombes,
France

This retrospective study collected data on 354 patients operated upon between 1978 and 1985 for gallbladder cancer. Gallstones were found as an etiological factor in 78 % of patients. Clinical symptoms included pain (60 %), jaundice (49.3 %), and fever (35.1 %). A significant weight loss was noticed in half of the patients. Imaging procedures included mainly preoperative ultrasonography and CT-scanning. However, the malignant condition of the gallbladder was only recognized in 13 % (ultrasonography) and 8 % (CT-scan) of cases. Thus, a clear preoperative diagnosis was made in only 30 % of cases. During operation, frozen section established the diagnosis in 32 % of cases.
Surgical management included cholecystectomy ( 55 %), and a large resection in 11 % of cases. Adjuvant radio- or chemotherapy was used in 9 % of cases.
Actuarial survival was 44 % at six months and 25 % at 12 months. Analysis showed that the best survival rates were obtained for tumours invading only the muscular layers and treated by cholecystectomy associated or not to a limited hepatic resection.
Adjuvant radiotherapy did not influence survival significantly.
This study suggests that gallbladder has a particularly poor prognosis because of the absence of specific symptoms which might lead to an early diagnosis. Ultrasonography appears to be the best imaging method for the diagnosis of gallbladder carcinoma.

### P0096 GASTRIC RECONSTRUCTION AFFECT CHOLELITHIASIS FOLLOWING AFTER GASTRECTOMY ?

Toshihiko Mayumi, Kitao Hachisuka,
Akihiro Yamaguchi, Masatoshi Isogai
Department of Surgery, Ogaki Municipal Hospital
Ogaki, Japan

Gastric surgery have been implicated in gallstone formation, although the association remains unproven. Whether gastric reconstruction affect in the formation of gallstones is still unknown.
To evaluate the influence of reconstruction on forming cholelithiasis, 91 patients were studied who were operated from 1978 to 1988 for cholelithiasis after gastrectomy. We classify these patients into four groups, A: proximal gastrectomy with esophagogastric anastomosis (3 cases), B: distal gastrectomy with Billoth I anastomosis (24 cases), C: distal gastrectomy with Billoth II anastomosis (55 cases), D: total gastrectomy with esophagojejunostomty (9 cases). In alphabetical order, the incidence of cholelithiasis of bile duct and bilirubincalcium stones was increased as well as the positive rate of the gallbladder bile incubation.
Especially in group A, all patients had colesterol stones and only gallbladder stones, on the other hand in group D, 6 of 9 patients had bile duct stones and all patients had bilirubincalcium or pigment stones. The period from gastrectomy to gallstone formation of group D was more rapid than other groups, significantly.
This study was done to underline not only gastrectomy but also gastric reconstruction have an influence on formation of gallstones.

### P0097 PORTAL AND SYSTEMIC HEMODYNAMIC CHANGES FOLLOWING SUGIURA PROCEDURE OR PORTOSYSTEMIC SHUNT

C VONS, A HADENGUE, C SMADJA, D GRANGE, D LEBREC, D FRANCO.
Hôpitaux Louise Michel, Evry ; Paul Brousse, Villejuif ; Inserm U24, Clichy. France.

The Sugiura procedure (SP) is better tolerated with respect to encephalopathy and liver function than portocaval shunts (PCS). The hemodynamic changes secondary to PCS may explain these differences. We compared the splanchnic and sytemic hemodynamics in patients with alcoholic cirrhosis, before and six months after either SP (9 patients) or PCS (6 patients). The wedged-free hepatic venous pressure gradient (HVPG), azygos blood flow (ABF), thermodilution total hepatic blood flow (THBF, indocyanin green continuous infusion), and cardiac output (CO, thermodilution) were measured in all patients. Results were as follows :

| | Portacaval shunt | | Sugiura procedure | |
|---|---|---|---|---|
| | before | 6 months after | before | 6 months after |
| HVPG (mm Hg) | 21 ±3.3 | 13 ±5.7* | 14.6 ±2.3 | 15.8 ±4.7 |
| ABF (L/min) | 0.5 ±0.3 | 0.2 ±0.03** | 0.47±0.2 | 0.46±0.2 |
| THBF (L/min) | 1.2 ±0.3 | 0.36±0.15* | 1.4 ±0.6 | 1.1 ±0.7 |
| CO (L/min) | 7.23±2.8 | 9.7 ±1.4* | 7.4 ±2.2 | 6.9 ±1.4 |

After PCS there was a decrease in HVPG (*, $p < 0.05$), THBF (*, $p < 0.05$), and an increase in CO (*, $p < 0.05$). ABF was decreased but not significantly (**, $p < 0.2$). After SP, there were no significant changes in HVPG, THBF, CO, or ABF.
These data, despite small groups, indicate : 1- esophagogastric devascularization does not change ABF suggesting maintenance of collateral flow ; 2- there is no increase in CO and therefore cardiac work in SP ; and 3- preservation of THBF in SP may explain decreased encephalopathy and better liver function.

### P0098 ADULT LIFE DUODENAL DUPLICATIONS (DD) AND CHRONIC PANCREATITIS

S.Vesentini, F.Nifosi', C.Bassi, M.G.Guiotto *, S.Corra' D.Lombardi, R.Girelli,G.C. Lazzarin,M.Falconi and P.Pederzoli

Surgical Department-University of Verona, Verona Italy.
* Medical Department-University of Verona, Verona Italy.

DD are seldom observed in adult life but are a well known cause of pancreatitis. DD are indeed an ideal clinical model to answer the questions about the relationship between obstruction and eventual evolution to chronic obstructive pancreatitis (COP) or to chronic calcifying pancreatitis (CCP). In recent years we were able to collect 12 adult life DD (all male patients, mean age 45.3 years). The mean duration of symptoms before the diagnosis was 5.1 years. Ten patients had been assuming more than 40 gr./die of alcohol for many years. The patients were all submitted to a window operation with or without associated procedures (eg cholecystectomy, biliary drainage etc.). Six pts.were followed for 2-7 years (mean 4 years). As for the aim of the present survey we would point out the followings: -
The two non-alcoholic pts.presented without COP or CCP; none of then has been yet controlled;
-Five alcoholic pts. presented with COP; two of them were controlled later (2 years): one patient developed diabetes and the other showed a full blown CCP; both of them did not stop alcohol intake;
-The remaining five alcoholic pts. presented with CCP; four showed at further controls a progressive pancreatic failure
Our experience seems to demonstrate that adult life DD can lead both to acute pancreatitis and COP, whereas the development of CCP is mainly dependent on alcohol consumption. Anyway this is an interesting problem which deserves further investigation.

### P0099 HEPATIC ARTERY BYPASS GRAFTING WITH THE RIGHT GASTROEPIPLOIC ARTERY

Toshiomi Kusano, Masato Furukawa,
Tsunoda Tsukasa* Ryoichi Tsuchiya*

Dept of Surg, Nagasaki Chuo National Hosp
2nd Dept of Surg, Nagasaki Univ Sch of Med*
Nagasaki, Japan

In the upper portion of hepatoduodenal ligament, the right hepatic artery runs upward between the bile duct and the portal vein. On the basis of this anatomical feature, resection of the right hepatic artery with extended hilar hepatectomy is often to helpful to remove residual tumor cells completely as radical treatment for hilar hepatic cholangiocarcinoma. However, end to end anastomosis of the right hepatic artery is too difficult to get late patensy.

So recently, we used the right gastroepiploic artery ( GEA ) to reconstruct to the circulation of the right hepatic artery in 2 patients with advanced hilar hepatic cholangiocarcinoma.

The pedicle, including the right GEA and surrounding tissues, was freed along greater curvature of the stomach. The GEA pedicle was raised up beyond the gasric pylorus and was anastomosed to the distal right hepatic artery by interrupted suture technique using 7-0 monofillament-nylon stitches.

The patients recovered well without evidence of anastomotic insufficiencies of hepatico-jejunostomy or liver dysfunction. Angiography one week after operation showed good patency of the GEA graft.

Pym and colleagues[1)] were the first to report the use of this conduit for direct coronary anastomoses when they described in situ right gastroepiploic grafts to the right coronary artery and posterior circumflex branches.

The method of hepatic artery bypass grafting with the right GEA is very simple and useful after combined resection of the right hepatic artery for surgical treatment of hilar hepatic cholangio-carcinoma. Forthermore, we recommend the GEA is an arterial conduit as an in situ free graft to any hepatic artery system.

References:

1) Pym J, Brown PM, Charrette EJP, et al 1987 ; 94 : 256-259.

### P0100 PRESSURE MEASUREMENT IN PANCREATIC AND BILIARY DUCT SYSTEM IN DOGS WITH ACUTE PANCREATITIS.

H.Fujiwara, Y.Kuroda, Y.Suzuki, Y.Fujino, Y.Ku, Y.Saitoh
Department of Surgery, Kobe University School of Medicine, Kobe, JAPAN

We investigated the influence of the periampullary inflammation caused by acute pancreatitis on the intraductal pressures of the pancreatico-biliary system. Severe and moderate acute pancreatitis were established by the injections of deoxycholic acid and trypsin respectively into the pancreatic duct of mongrel dogs. Manometoric catheters were placed in common bile duct (CBD) and pancreatic duct (PD), connected to the transducer and multichannel polygraph. Under the perfusion with saline (CBD: 0.8 ml/min, PD: 0.2 ml/min), two parameters, residural pressure (RP) and pressure decay time (DT), were measured at 6, 12hours, 1, 2, 3, and 4days after inductions of acute pancreatitis (Matsushiro, 1984).

RP of PD was 39.5±4.7 (mean±SD) $cmH_2O$ in controls. Significant increase in RP of PD (72.6±37.0 $cmH_2O$) was observed in severe pancreatitis on day 2, while the correspondig value in moderate pancreatitis was 46.0±10.4 $cmH_2O$. In contrast to the value in controls (15.7±9.5 sec), significant prolongation of DT of PD was demonstrated in severe pancreatitis (56.7±30.5 sec on first day). In addition, DT of PD in severe pancreatitis was significantly prolonged as compared to that in moderate pancreatitis ($P<0.05$). RP of CBD were simillar to that of PD, but there was no significant change in DT of CBD during observations. In conclusion, pressure measurments of PD may be worthwhile predicting the severity of acute pancreatitis.

References:
Matsushiro T, Nagashima H. SURGERY 1984; 95: 96.

P0101 THE DYSFUNCTION OF CANINE SPHINCTER OF ODDI AFTER GASTRECTOMY

M. Mori, T. Yamasato*, H. Mimura, K. Orita
Department of Surgery and Physiology*,
Okayama University Medical School, Japan

The increased tendency of stone formation in the gallbladder and choledochus after gastrectomy has been reported by many authors. In this study, to elucidate this mechanism, the motility of the sphincter of Oddi and it's response to caerulein after gastrectomy were observed in mongrel dogs.

Two groups of the distal half gastrectomy and the sham operation were prepared. Anesthesia was induced by pentobarbital sodium and maintained by gallamine in controlled respiration. The motility of the sphincter of Oddi was recorded by the pressure transducer with constantly perfusing (0.12ml/min.) method through a cannula placed into the common bile duct.

In the control group, the perfusion resistance and frequency of phasic contraction of the sphincter were 4-6cm $H_2O$ and 4-6c/min. respectively, and the both were decreased by caerulein (10ng/kg, i.v.). Their behavior was same after the sham operation. However, in the acute and one month chronic gastrectomized group, the perfusion resistance and frequency of phasic contraction of the sphincter were increased to 7-11cm $H_2O$ and 7-10c/min. respectively, and the caerulein-induced inhibitory response was changed to excitatory response. Bilateral cervical vagotomy, splanchnicotomy and transection of the gastric antrum didn't affect the caerulein-induced inhibitory response of the sphincter, but transection of the duodenal bulbus did change the response into excitatory one.

In summary, the dysfunction of the sphincter of Oddi was induced by gastrectomy, that was the increase of the perfusion resistance and frequency of phasic contraction, accompanied by the reversed response to caerulein. This suggests a genetic mechanism of clinical cholelithiasis after gastrectomy.

References
Wyatt A.P. Gut 1969: 10: 91
Anderson J.R. et al Br. J. Surg. 1980: 67: 618
Behar J. et al J. Clin. Invest. 1980: 66: 1231

### P0102 FLUIDITY OF LEVER PLASMA MEMBRANE IN DIFFERENT LIVER DISEASES

T.Kasamatsu, J.Tanaka, M.Yoshida,
J.Tamura, K.Fujita, S.Arii, T.Tobe
First Department of Surgery,
Kyoto University School of Medicine,
Kyoto, Japan

The membrane fluidity is one of most important determinants for cellular function, for instance, in substance transport, receptor function and so on. The present study was aimed to investigate the fluidity of plasma membrane isolated from surgically biopsied materials of livers with cirrhosis, jaundice or in regeneration process. Also, an attention was paid at possible direct effects of plasma of patients under multiple organ failure on membrane fluidity. The membranes were isolated by centrifugation and fluidity was determined by measuring fluorescence polarization using 1,6-diphenyl -1,3,5-hexatriene as a probe dye. The fluorescence polarization (p) of normal control was 0.193±0.005 (mean +S.E., N=18). Jaundiced liver showed 0.222±0.008 (N=4, $p<0.02$). Similarly, cirrhotic liver also showed increased p-values up to 0.211±0.005 (N=14)($p<0.02$, compared to control). By contrast, the regenerating liver indicated a restoration of the elevated p-value (0.190±0.007, N=4). Thus, diseased livers generally represent an increase in polarization, in other words, a decrease in membrane fluidity. An addition of the plasma of patients under multiple organ failure to normal liver plasma membrane during the incubation with the probe dye induced to a marked elevation of p-value and a marked decrease in integrated fluorescence intensity, which is quite different from the result of normal plasma, that is, increased integrated intensity without changes in p-values. Above results suggest that deterioration of membrane fluidity is an indicative of impaired cellular function and that multiple organ failure may be evoked by the unknown humoral factor(s) leading to an alteration of membrane fluidity.

### P0103 PERCUTANEOUS MANAGEMENT PYOGENIC HEPATIC ABSCESSES

R. Andersson, L. Forsberg, E. Hederström, S. Bengmark.
Departments of Surgery and Roentgenology, Lund University, Sweden

The outcome of patients with hepatic abscesses has previously been poor. During the last decade a marked decrease in mortality has been noted in association with the introduction of new imagine techniques, such as computed tomography and ultrasonography, leading to a decrease in diagnostic delay and making percutaneous drainage possible.

During the period 1979 to 1989 17 patients (12 men, 5 women; mean age 63 years) were treated with percutaneous drainage. The length of illness before admission was in mean 25 days. Biliary origin was most common (6 patients), followed by hepatic abscesses occurring as a late postoperative complication (seen in 3 patients) and hepatic abscesses occurring in association with acute appendicitis (2 patients). The origin was unknown in 6 patients. Diagnosis was reached by computed tomography or ultrasonography with a diagnostic delay of in mean 11 days. 22 abscesses were found among the 17 patients. The median abscess size was 7 cm.

12 patients were treated with percutaneous drainage with an indwelling catheter within the abscess cavity for in median 2 weeks, while 5 patients were managed with percutaneous puncture and aspiration alone. Bacterial cultures were positive in about 3/4 of patients with E.coli being the most commonly isolated organism. All patients survived. The course following percutaneous puncture was uneventful and no complications attributable to the percutaneous procedure per se were seen. In one patient where technical reasons contraindicated a repeated percutaneous drainage, surgical drainage of an additional hepatic abscess was required.

Percutaneous drainage of hepatic abscesses seems as a safe and reliable method which should be considered as the treatment of choice if facilities and knowledge of percutaneous management are provided.

### P0104 SURGICAL MANAGEMENT OF BENIGN LIVER TUMORS

H. Sanchez, M. Gagner, R.L. Rossi,
R.L. Jenkins, W.D. Lewis, J.W. Braasch,
Department of Surgery, Lahey Clinic & New
England Deaconess Hospital, Burlington &
Boston, MA, USA.

The improvement in diagnostic methods has increased the incidence of benign liver tumors (BLT) and brought difficulties in surgical management. The aim of the study was to compare and assess the surgical management in hepatic hemangiomas (HEM), adenomas (ADEN), and focal nodular hyperplasias (FNH). We reviewed the records of 47 patients with BLT who had 52 surgical procedures. There were 31 patients with HEM, 6 ADEN and 10 FNH. Mean age was 54 and 61% were females in HEM, whereas mean age was 36 and 94% were females in ADEN and FNH. The most common symptom was chronic abdominal pain in ADEN and FNH. Two thirds of HEM were asymptomatic. Oral contraceptive use was associated with more than half of ADEN and FNH but not HEM. The right lobe was the most frequent location; 49% of HEM, 62% of ADEN and 80% of FNH. Angiogram and secondarily liver scan were the most diagnostic test in ADEN and FNH, whereas angiogram and MRI were the most diagnostic in HEM. CT and U/S were not diagnostic for any ADEN, and only 40% of hemangiomas.

| | Biopsy | Enucleation | Resection | OLTx |
|---|---|---|---|---|
| HEM | 8 (3) | 9 | 20 (1) | 0 |
| ADEN | 0 | 0 | 3 | 2 |
| FNH | 1 | 2 (1) | 7 | 0 |
| TOTAL | 9 (3) | 11 (1) | 30 (1) | 2 |

() recurrences. The median follow-up (excluding deaths) is 12 months.

Biopsy was most frequently performed for HEM, and when done for symptomatic patients, recurrences were high. Resections are 3 times more commonly performed than enucleations. Resections and enucleations are curative and recurrences are rare. Liver transplantation (OLTx) was performed for a well diff. hepatoma from an adenoma and for adenomas associated with a glycogen storage disease. Enucleation or resection is the treatment of choice for symptomatic BLT, asymptomatic ADEN or when there is an uncertainty of diagnosis.

**PO105** BILIARY LITHOTRIPSY: WHO SHOULD BE DOING IT?

J.G. Rothschild, M.D., R.B. Reinhold, M.D.
New England Medical Center, Boston, MA

With the expansion of lithotripsy technology from renal stones to biliary stones, the question of who should be undertaking biliary lithotripsy has arisen. Issues of radiographic, i.e. ultrasonic, localization, surgical decision making and managing gastrointestinal symptoms have all been used to define primary lithotriptors.

Using our experience as part of the Dornier National Biliary Lithotripsy Study (DNBLS), we investigated the relative outcomes of surgeons, radiologists and gastroenterologists. We compared results with respect to length of treatment, treatment related complications, need for retreatment, success in fragmentation and six month stone-free rates to assess ability in biliary lithotripsy.

Grouping the centers into those where surgeons or gastroenterologists alone provided treatment, those where radiologists ran the technology, and those where both surgeons and gastroenterologists worked with radiologists on all treatments, we found results as noted in the table below:

| | Surgery Alone | Radiology Alone | GI Alone | Surgery/GI w/RAD |
|---|---|---|---|---|
| Stone-free (%) | 16.8<br>(28.8vs4.5)* | 17.8<br>(19.4vs16.2)* | 8.9<br>(10.8vs6.9)* | 14.5<br>(21.8vs7.7)* |
| Fragmentation(%) <5.5 mm | 53.6 | 77.2 | 46.2 | 41.9 |
| Technical** Complications (%) | 31 | 8.4 | 28.9 | 30.6 |

* UDCA vs placebo
** Unsatisfactory positioning, instrument malfunction, etc.

We conclude that surgeons are well capable of mastering the ultrasonic localization of stones required for biliary lithotripsy. When surgical experience in decision making in biliary disease is taken into account, surgeons might best be able to play a primary role in lithotripsy of gallstones.

### P0106 PROGNOSTIC FACTORS IN SURGICAL PATIENTS WITH HEPATOCELLULAR CARCINOMA IN KOREA

S.T. Kim, D.Y. Noh, H.J. Lee, Y.I. Kim
Seoul Nat'l Univ. Hosp., Seoul, KOREA

The prognostic importance of 13 factors were analysed using univariate and multivariate regression models in 217 patients with hepatocellular carcinoma who had curative or palliative hepatic resections during 10 year period since 1978. The patients who had curative resection had better survival rate than the survival of the palliative resected patients ($P < 0.025$).

Univariate analysis of curative resected cases revealed that Child's classification, resectional type, encapsulation and microangioinvasion affected significantly to the longterm survival ($P < 0.05$) but sex, age, size, AFP, cirrhosis, transfusional amount didn't have statistical meanings.

Multivariate analysis showed microangioinvasion, Child's classification and resectional type were the most valuable factors in predicting survival in decreasing order. The predictability of the prognosis by histopathologic subtypes is also evaluated. The male to female ratio was 5 to 1 and HBsAg was positive in 74.8% of these patients and 2 year and 5 year survival of curative resected patients were 43.7% and 31% respectively.

P0107 **HEPATIC INCIDENTALOMA - A MODERN PROBLEM**

T. Wilson, M. Hollands, J. M. Little.
Westmead Hospital, Sydney, Australia.

With increased reliance on organ imaging rather than clinical skills an increasing number of solid filling defects in the liver of well patients are being identified. The aim of this study was to consider the likely diagnoses of such lesions, to determine how symptoms and signs relate to the diagnosis and to suggest a practical plan of investigation.

All patients with solid undiagnosed liver lesions referred between October, 1985 and October, 1988 were reviewed. Cystic lesions, biopsy proven lesions and lesions detected at routine follow-up following cancer surgery or in patients with hepatitis B were excluded. 36 patients were seen over a 3 year period. 29 lesions (81%) were benign. 24/29 (67%) had non-neoplastic conditions and 5 had benign tumours (14%).The remaining 7 had malignant lesions (19%), 5 being secondary tumours and 2 primary tumours. Hepatic haemangioma was the commonest single diagnosis (20/36 patients; 56%). Patients with physical signs of a liver mass or liver enlargement were more likely to harbour a malignancy. An elevated serum alkaline phosphatase was suggestive of malignancy.

As a result of this study we suggest the following protocol. Liver function tests, serological tests for CEA and AFP and a blood pool scan may be performed as an outpatient. If no clear diagnosis is obtained angiography, CT-angiography and fine needle cytology may be required.

P0108 **OBSTRUCTIVE JAUNDICE CAUSED BY HEPATOCELLULAR CARCINOMA**

W.Y. Lau, A.K.C. Li. Department of Surgery, The Chinese University of Hong Kong, Prince of Wales Hospital, N.T., Hong Kong.

Obstructive jaundice as the main presenting clinical feature of hepatocellular carcinoma (HCC) is uncommon. The major bile ducts can be obstructed by intraluminal necrotic tumour, haemobilia, direct tumour infiltration or tumour compression.

In the past 4 years, we saw 340 patients with HCC. Blood tests showed 69% of patients had a raised total bilirubin level (upper limit of normal = 15 umol/l). However, significant jaundice was detected clinically in 75 patients (22%). Routine ultrasound detected dilated intrahepatic ducts in 11 patients. The remaining 64 patients were in the terminal stage of malignancy. Forty one of them died within 4 weeks.

The total bilirubin and alkaline phosphatase levels (mean $\pm$ s.d.) of the 11 patients with obstructive jaundice were 263 $\pm$ 188 umol/l (mean > 16-fold increase) and 575 $\pm$ 208 1u/l (mean > 4-fold increase) respectively. Endoscopic retrograde cholangiopancreatogram (ERCP) revealed extensive tumour involvement in 8 patients. Jaundice was relieved by endoscopic endoprosthesis in 4 patients, nasobiliary drainage in 2 patients, percutaneous transhepatic stenting in 1 patient and surgical intubation in 1 patient. The survival interval of these 8 patients (mean $\pm$ s.d.) was 35 $\pm$ 20 days. Tumour fragments were shown in the common bile ducts by ERCP in the remaining 3 patients. In 2 patients, major hepatic resection was done after initial tube decompression of the biliary systems. One patient remained tumour free on follow-up at 24 months and the other patient had recurrent tumour detected on follow-up at 17 months after surgery. The tumour was irresectable in the third patient. Multiple surgical and endoscopic procedures kept the bile duct patent for 17 months before the patient finally succumbed to the disease.

The prognosis of patients with HCC is dismal. However, it is important to recognise the group of patients with major bile duct obstruction. With proper management, good palliation, and occasional cure, is still possible in some of these patients.

P0109 SURGICAL TREATMENT OF HIGH BILE DUCT CARCINOMA. MANAGEMENT AND ANALYSIS OF 34 CASES

T.Kakavoulis, G.Stylianides, J.Nomikos
Evangelismos Hospital, 1st,3rd Surgical Unit, Athens, Greece

From January 1979 up to December 1987 34 patients, aged between 35 to 70 with high bile duct carcinoma, were operated on, in the 3rd and the 1st surgical units of the Evangelismos Hospital. In all cases the preoperative diagnosis was made with the percutaneous Transhepatic Cholangiography (PTC) and confirmed by the operative findings and tumor biopsy. Only 5(14,7%) of the 34 patients underwent tumor resection; nine underwent left hepaticojejunostomy by using the Round Ligament Approach. Two patients underwent right hepaticojejunostomy using the mucosal graft approach and two others underwent partial resection of the quadrate lobe and anastomosis of both hepatic ducts with jejunum. The remaining sixteen patients were treated by dilatation of the tumor stricture and insertion of a transhepatic silastic tube, of which sixteen patients, four died in the hospital and seven had to be reoperated on due to bouts of cholangitis and dislodgement of tubes.
Two patients who had undergone tumor resection are still alive 24 and 36 months respectively. The average postoperative survival rate in the tumor resection group was 25,6 (16-46) months. In the operative internal bypass it was 13(8-20) months and in the case of the patients who had undergone intubation it was 13,3 (1-53) months. Tumor resection appeared to have the best long term prognosis. Concerning the other two procedures, the hepaticojejunostomy gave good palliation and presented fewer complications than in the case of intubation.

### P0110 IMPACT OF THE CUSA AND OPERATIVE ULTRASOUND ON HEPATIC RESECTION.

J. M. Little, M. Hollands, T. Wilson.
Westmead Hospital, Sydney, Australia.

New technologies applicable to liver surgery have recently been developed. This study was performed to determine whether they are cost effective and whether they make liver surgery safer.

50 elective hepatic resections performed over a 66 month period were reviewed. Operative techniques were standardised throughout the period of the study.

28/50 resections were performed for tumours. The median duration of the operations was 181 minutes (range 90-400). The median portal clamping time was 15 minutes (range 5-35). The blood loss ranged from 100-4500 mls (median 450mls). 20 patients required transfusion. The diagnostic ultrasound was used 23 times and the CUSA 22 times. Patients in whom the CUSA was used were significantly older (p=0.0063), portal clamping times were significantly longer (p=0.031), blood loss was significantly less (p=0.0039) and fewer patients required transfusion (p=0.0001). The use of operative ultrasound did not increase median operating time (168 minutes compared with 185 minutes, p=0.1103). Principal components analysis suggested that the experience of the operator coupled with the least possible operation were the factors most likely to determine a straightforward post-operative course.

Operative ultrasound allowed more precise planning of operations and its use was not associated with increased operating times or post-operative morbidity. The CUSA allowed lower blood loss, fewer transfusions and a shorter post-operative stay. The 2 devices offered measured savings by a reduction of hospital stay of 4.5 days, a saving of 700 mls median blood requirement and a fall in the transfusion rate from 64% to 9%.

P0111

### CAN FIBRONECTIN PREDICT THE OUTCOME OF ACUTE PANCREATTIIS?

C Wilson, D Maharaj, F McCall, C Gemmell,
C W Imrie. Departments of Surgery,
Haematology and Bacteriology,
Royal Infirmary, Glasgow, UK.

Low fibronectin levels occur in septicaemia and following major surgery or burn injury. In critically ill patients fibronectin-rich infusions improve cardiopulmonary function and tissue oxygenation. We have studied the sequential changes of plasma fibronectin in 25 attacks of acute pancreatitis (24 patients; males - 20, females - 4, mean age 49, range 26-86 years). Aetiologies comprised alcohol - 15, gallstones - 6 and various causes - 4.
Two patterns emerged; 17 patients showed a rising (16) or stable (1) fibronectin pattern throughout their illness, all but one (with respiratory insufficiency) having an uncomplicated clinical course. Eight patients had a falling fibronectin pattern (6) or subnormal levels on the single samples available prior to their death (2). Of the 6 with a falling pattern, 2 developed pancreatic necrosis (both died), one a pseudocyst, one respiratory insufficiency and 2 had a mild, uncomplicated attack. Three of the 4 dying had subnormal fibronectin levels recorded. Fibronectin appears a useful marker of severity in acute pancreatitis, a low or falling pattern predicting a complicated attack in 6 of 8 patients. Reports of success with exchange transfusion or plasmapheresis in patients critically ill with pançreatitis might be explained by the effect of repleting plasma fibronectin.

P0112 SPECIAL FORM OF OBSTRUCTIVE JAUNDICE

D.Z.Xu, H.W.Yang, H.D.Wang,H.G.Huang,
XinJiang Medical College, CHINA

Reported 48 cases of 2 forms of rare special obstructive jaundice due to the rupture of hydatid cyst into bile duct and the invasion of the parasite of liver alveolar echinococcosis.
Both forms were 32 and 16 cases respectively, male 19, female 29, age 13 to 81, 87.5% under 50 years old.
The chief manifestations were low grade fever, jaundice, discomfort of abdomen, tenderness, leucocytosis,eosinophilia, damage of liver function with elevation of SGPT and AKP. 30/48 cases complicated with cholangitis.
Further examinations may be necessary including Casoni test, B-ultrasonic scanning, CT, plain x-ray film, ERCP or PTCD. The diagnosis usually is not difficult, however, it should be differentiated from choledocholithiasis, primary carcinoma or metastasis of liver, hemangioma of liver and hepatic abscesses.
Evacuation of the small hydatid cysts and its débris from the bile duct and drainage with T tube in order to relieve the obstructive jaundice is the first choice in surgical treatment, where as the hemihepatectomy or irregular resection of liver with the lesion should be performed in the alveolar form, while for the irradicable cases, palliative surgery and chemotherapy are useful in relieving the compression of bile ducts.
39 cases recovered, 7 cases failed to surgical operation and 2 cases died of complications.

### PO113 STUDY OF SUBHEPATIC FLUID COLLECTIONS AFTER CHOLECYSTECTOMY WITH AND WITHOUT DRAINAGE.

A.R.Ferreres, J.A.Diez, V.P.Gutiérrez,C.Ruiz

Departamento de Cirugía. Hospital de Clínicas "José de San Martín". Universidad de Buenos Aires.

Different authors have found the increased presence of clinically unsuspected subhepatic collections after cholecystectomy. This study was undertaken to assess the significance of postcholecystectomy fluid collections detected by ultrasound and to correlate them with the use of drains.

We studied a group of 60 patients who underwent elective cholecystectomy, with suture of the gallbladder bed. The age ranged from 22 to 74(mean 60.29) and 52 were women. Independent of the findings at the operation, a sump drain was placed for 2 days in 30 patients and no drain was left inthe other 30.All patients were examined with ultrasound scans at the second postoperative day, before removing the drain, and at the seventh postoperative day.

In the group without drainage no study discovered fluid collection and in the group with drains, 2 patients presented subhepatic collections less than 15 cc. Both were asymptomatic and disappeared at the seventh day, as confirmed by ultrasound.

The incidence of postcholecystectomy fluid collections is not significant. The results suggest that surgical drainage after elective cholecystectomy is unneccessary if surgeons use them for draining subhepatic collections and that no infected collections resorb spontaneously.

References:

Diez JA, Pujato MR, Ferreres AR. HPB Surgery (in press)
Elboim CM, Goldman L, Hann L. Ann. Surg. 1983: 198: 137
Elyaderani MK, Skolnick ML, Weinstein BJ. Surg., Gyencol., Obstet. 1979: 149: 529
Maull KI, Daugherty ME. J. Surg. Res. 1978: 24: 259
van der Linden W., Kempi V, Gedda S. Ann. Surg. 1981: 193: 155

### P0114 NEW CRITERIA OF PATIENTS SELECTION FOR EXTRACORPOREAL PIEZOELECTRIC LITHOTRIPSY (EPL)

V S Saveliev, Yu G Starkov
2nd Medical Institute, Moscow, USSR

The efficiency of EPL for cholelithiasis patients is not high (34%) (Ell et al, 1988) and a considerable proportion (35%) of patients suffer from biliary pain during the first month after EPL (Thiel et al, 1989). In order to achieve more effective EPL we have elaborated and applied two additional criteria for patient selection.

In addition to conventional criteria we have used for the first time the physical characteristics of the echostructure of the stones. We have found the limit of the echointensity and echopermeability of stones (in dB) within which highly effective stone disintegration into small particles (0, 1-0, 3 cm) is possible.

Moreover, a new criterion was introduced - the ratio of the total stone volume to the amount of bile secreted in a single gallbladder contraction.

EPL (Wolf-Piezolith-2300) was used for 76 patients suffering from both radiolucent and radio-opaque stones. The overwhelming majority of the stones was successfully fragmented. Within the 12-week observation period 56 patients (74%) eliminated all stones. Only 6 patients (8%) experienced biliary pain during 3 months after EPL.

References:

Ell, Ch et al. Dtsch med Wschr 1988, 113; 1503-1507
Thiel H et al. Z Gastroenterol 1989, 27; 263-266

### P0115 REGIONAL THERAPY FOR LIVER METASTASES: EXPERIENCE WITH HEPATIC ARTERIAL CATHETERS

J.A. Goldberg, C. Morran, D.P. Leiberman,
I. Stewart, D.J. Kerr, C.S. McArdle.
Royal Infirmary, Glasgow, Scotland, U.K.

Conventional forms of therapy for patients with multiple, bilateral colorectal liver metastases have been shown to be largely ineffective. Attention has therefore turned to the concept of regional chemotherapy which, in theory, should increase drug levels within the tumour and reduce systemic exposure. Complex surgical procedures have been described to insert and maintain intrahepatic arterial catheters. We describe a novel method of inserting catheters in patients with an anomalous blood supply and replacing blocked catheters.

Forty-five patients with histologically proven liver metastases were referred for regional chemotherapy. Eleven patients were found to have extrahepatic disease; the remaining 34 patients had an indwelling hepatic arterial catheter inserted.

Twenty-eight (83%) patients had a single common hepatic artery; the catheter was inserted in the classical fashion. A dual blood supply was present in six patients; in these patients, double catheters were inserted using a vein graft as a conduit.

Catheter blockage occurred in five patients; the blocked catheters were replaced again using a vein graft as a conduit. Sepsis around the portal occurred in two patients; both were successfully resited.

We have therefore simplified the surgical approach to intrahepatic arterial catheter insertion and demonstrated that we can achieve long-term catheter patency.

### PO116 ELEVATED LEVELS OF TUMOR MARKER CA 19-9 AND BILIARY OBSTRUCTION

G. Giorgianni, A. Artemisia,
A. Cogliandolo, M. Gioffrè, B. Micali
General Surgery, University of Messina,
Italy

Ca 19-9 has been reported as a more sensitive marker for pancreatic than colorectal carcinoma (1). It would seem, however, that serum Ca 19-9 is occasionally elevated in patients with benign biliary disease ie sepsis. A direct relationahip was shown, in fact, between acute cholangitis and raised levels of the antigen, the common duct obstruction playing a little part in Ca 19-9 elevation (2).

Materials & Methods Thirty-nine patients with benign biliary disease entered this study. Twenty patients were jaundiced (J) and 19 were not jaundiced (NJ). Among the J group 11 had signs of acute cholangitis (JAC) and 9 had no signs of cholangitis (JNAC). Furthermore, among all patients 7 had evidence of chronic cholecystitis (CC) and 6 were previously cholecystectomised (PC). Sera were obtained from all the patients and stored at -20oC until assayed for Ca 19-9 (commercial reagents from CIS-Diagnostics Laboratories). The cutoff of our laboratory from 40 healthy subjects was previously calculated at 13.1 U/ml (3).

Results Ca 19-9 levels in J and NJ patients were 61.7 U/ml and 14.6 U/ml respectively (p 0.05). In JAC patients the Ca 19-9 levels were 45.1 U/ml whereas in JNAC were 36.5 U/ml (NS).

Conclusions From these results, it is concluded that Ca 19-9 marker can be elevated in disorders other than cancer disease. Benign obstructive jaundice would appear to be a condition associated with the most significant increase suggesting a poor diagnostic role of Ca 19-9 in differentiating benign from malignant jaundice.

Biliary inflammation did not lead a significant elevation of serum Ca 19-9 thus supporting that the antigen, as CEA studies resulted (4), crosses the bile-blood barrier by jaundice exclusively.

References:

1) Steinberg W M, Gelfand R et al Gastroenterology 90:343-9 1986
2) Albert M B, Steinberg W M et al Gastroenterology 92:1292, 1987 (Ab
3) Gioffrè M A, Venuti A et al Il Policlinico 91:1-3, 1984
4) Carr-Locke D L, Gut 21:656-661, 1980

P0117 ANTITUMOR EFFECTS OF SCLEROSING AGENTS AGAINST HEPATIC VX2 CARCINOMA-BEARING RABBITS

N. Tsukuda, H. Taniguchi, A. Oguro, T. Daidoh, A. Itoh, Y. Shioaki, T. Takahashi
First Department of Surgery, Kyoto Prefectural University of Medicine, Kyoto, Japan

Like as ethanolamine oleate (EO), Aethoxysclerol (AS) which are applied to endoscopic injection sclerotherapy fir esophageal varices, and an oil emulsion of phenol (P) that is used in sclerotherapy for hemorrhoids, damage to the injected site because of making thrombus or fibrosis in their pharmacological actions. It is guessed that the application of these agents to the tumor vessels will kill carcinoma cells.

We examined the antitumor effects of intraarterial injection of 5% EO, 1.25% EO emulsion using Lipiodol which is a lipid contrast medium, 1% AS, 0.5% AS emulsion using Lipiodol, and 5% P emulsion using Lipiodol injected intraarterially against VX2 caricinoma metastasizing to the rabbit liver. EO (0.25ml/kg), EO emulsion (0.25ml/kg), AS (0.25ml/kg), AS emulsion (0.25ml/kg), and P emulsion (0.25ml/kg) were administered into the hepatic artery. Only mild liver damage was noted after infusion of each drug. The effectiveness of each agent was histologically evaluated according to the percent necrosis. From 50 to 90 % of necrosis was observed in all groups, and emulsion of EO, AS, and P were more effective than EO and AS.

It is concluded that targeting chemotherapy using the sclerosing agents against to the tumor vessels is useful for intraarterial infusion chemotherapy of the liver tumor.

P0118 SELENIUM'S INHIBITION OF PANCREATIC CANCER INDUCED IN HAMSTERS INDUCED BY N'-NITROSOBIS(2-OXOPROPYL)AMINE

Y.Kise, M.Yamamura, M.Kogota, S.Uetsuji, A.H.Kwon, K.Hioki, M.Yamamoto
Department Of Surgery, Kansai Medical University, Osaka, Japan.

We investigated the inhibitory effect of Se in drinking water against pancreatic cancer in female Syrian golden hamsters induced by N'-nitrosobis(2-oxopropyl)amine (BOP). Four-week-old female Syrian golden hamsters were divided into 2 groups according to the Se level contained in the drinking water, 0.1ppm and 4.0ppm. All the hamsters were fed a purified diet containing less than 0.05ppm of Se. Four weeks later, some hamsters were subcutaneously administered BOP at 10mg/kg body weight 10 times weekly, while others were similarly administered saline as controls. Eighteen weeks after the last injection, the hamsters were sacrificed for histological investigations of pancreas. The rate of palpable cancers was 57% in the high Se BOP group and 71% in the low Se BOP group. The number of ductular adenocarcinomas was 2.17±1.62 in the high Se BOP group and 3.41±2.73 in the low Se BOP group. No tumors were detectable in the saline treated groups. Throughout the 31-week experiment, Se levels and glutathione peroxidase activities in serum and pancreas were significantly higher in the high Se groups, suggesting that the glutathione peroxidase acts a great role in inhibition of pancreatic carcinogenesis by Se.

### P0119 ELECTROMYOGRAPHIC ACTIVITY OF THE GASTROINTESTINAL TRACT FOLLOWING CHOLECYSTECTOMY

J.C.U. Coelho, C.A. Pupo, A.C.L. Campos, A.A. Moss Jr.

Federal University of Paraná, Curitiba, Paraná, Brazil

Electromyographic activity of the stomach and small bowel, both in the fasting and fed states, was evaluated in the postoperative period of eight patients subjected to cholecystectomy. The migrating motor complex (MMC) was recorded on the first postoperative day in five patients, on the second day in two, and on the third day in one. Vomiting occurred in one patient in whom the MMC was recorded only on the third postoperative day. Feeding caused substitution of the MMC by the fed pattern in the stomach and small bowel in all patients. It is concluded from this study that gastric and small bowel motility is normal on the first two days of the postoperative period in most patients subjected to cholecystectomy.

PO120

### FOCAL NODULAR HYPERPLASIA - A PLAN OF MANAGEMENT

J.Pain, E.Howard
King's College Hospital, London, UK

Between 1973 and 1989 22 patients (19 females) with focal nodular hyperplasia (FNH) were seen at King's College Hospital. There were five children, and all adults were aged under 42 years (median 33 years). Fourteen patients (64%) were symptomatic on presentation. FNH was an incidental finding at postmortem in one patient. Twelve of the 14 adult females had taken oral contraceptives (OCP). Histological confirmation of FNH was obtained in all patients.

Twelve patients, nine of whom were symptomatic, underwent hepatic resection shortly after presentation. There were no deaths or major complications, and all remain well on follow up. Four patients underwent either hepatic artery embolisation or ligation. At follow up between six and 10 years they are asymptomatic and only one has histological evidence of residual FNH.

Of five patients initially treated conservatively two were asymptomatic and have remained so for three and 13 years. Three were symptomatic: one became symptom free after stopping the OCP. The two other patients continued to have symptoms, and one of these underwent FNH resection three years later.

The management of FNH requires a flexible approach. Lesions which are asymptomatic can be observed with regular ultrasound, and treated if they enlarge or become symptomatic. Symptomatic patients presenting whilst taking OCP can also have a trial of conservative treatment. Other symptomatic patients, including those who previously took OCP, are best treated by surgical resection, and, where not possible, by embolisation.

### P0121 DECISION MAKING IN THE SURGERY OF ECHINOCOCCAL CYSTS

Z. Ráth, F. Jakab, J. Faller, I. Sugár

Semmelweis Med Univ, Dept of Surgery,

Budapest, Hungary

The pitfalls in the surgery of Echinococcal cysts are well known even in recent days in spite of new pre-operative diagnostic modalities. There are still unexpected findings intraoperatively. The aim of this study is to collect cases from our past material characterizing the decision making in the surgery of Echinococcal cysts.

The major liver resection - first of all the right and left trisegmentectomy are preferred mainly in the cases of Echinococcas alveolaris, which regarded basically to be very infrequent. On the basis of ultrasound, CT and angiography and last, but not least, intra-operative findings, the indication of trisegmentectomy could be decided. A characterizing case of a 44 year old woman is presented.

The central localisation of the Echinococcal cyst is considered problematic if the hepatic vein or inferior vena cava is involved. The partial resection or capitonnage are treatments of choice. On the other hand, the central localisation of the Echinococcal cyst could be complicated by direct communication with the hepatic ducts.

The so-called 'floating membrane' phenomena could cause jaundice by obstruction of the common bile duct. The surgical intervention should include the resectional procedure, the removal of floating membrane from the common bile duct and effective drainage of the biliary tree.

For the demonstration of each form the characterizing cases are presented.

### PO122 ACUTE CHOLECYSTITIS: SURGICAL TREATMENT

R.Jashari,R.Binishi,A.Krasniqi,

D.Limani,Sh.Robaj,A.Rrusta

Clinic of surgery and Ortopedy,

Prishtina,YU

In this study we analysed all the patients with acute cholecystitis, treated in the time period of last two years (01.01.88-31.12.89). There were 25.238 patients hospitalized in our department and 779(3.8%) suffering from gall-stones. 612 (78,56%) were operated and 167 (21,43%) treated without surgery (medicamentously).247 patients with clinical and laboratory signs of acute cholecystitis made only 31,70% of all patients (779) suffering from gall stones. 80 (32,38%) of them were treated surgicaly and 167 (67,61%) without surgery. In 19 (23,75%) operatively treated we found gangrenous cholecystitis, among of which 4 where perforated with developed biliary peritonitis and 12 without perforations. In 3 of them was found cholecysto-enteric fistula (2 in colon transversum and 1 in duodenum).

We reserved colecystectomy in the acute stage for the cases with tendency for the perforation or perforated cholecystitis with peritonitis.

Operative complication-we had in one case-lesion of cystic artery. Postoperatively we have one bleading from cystic artery, and three lesions of main bile duct, one pneumonia and three wound infections.

Death rate was one patient out of 612 operated patients or 0,16%.

References:

1. Rensburg L.C.:The menagment of ac. cholecystitis in the elderly, Br J Surg. 1984, 71: 692-3.

2. Way L.W.:In Curent surgical diagnosis and treatment Lange medical book, 1988.

3. Issa M. et all.:Early surgical treatment of acute cholecystitis. J Chir (Paris) 1984, 121: 101-4.

4. Herman R.E.: The spectrum of biliary stone disease, Am J Surg 1989; 158: 171-3

5. McSherry Ch.K.:Cholecystectomy: The gold standard Am J Surg 1989; 158: 174-8

### P0123 ELECTIVE CHOLECYSTECTOMY IN PATIENTS WITH HOMOZYGOUS BETA-THALASSAEMIA

N.Legakis, G.Bonatsos, G.Sakellariou, G.Velmachos, P.Peveretos, B.Golematis

First Dept. of Propaedeutic Surgery, Athens University, Greece.

Chronic hemolysis predisposes patients with homozygous beta-thalassaemia to the formation of pigment gallstones. To eliminate the risks of subsequent anaesthesia and surgery in this vulnerable group of patients we have adopted a particular strategy in their surgical management. At the same laparotomy for the inevitable splenectomy, we perform cholecystectomy as well as an incidental appendectomy with the same rationale: to eliminate the risk of subsequent surgery. The indication and the timing of splenectomy is decided upon by the haematologists and is mainly based on the presence of excessive splenomegaly and/or hypersplenism.

Our patients material consists of 168 children and young adults (mean age 20.8 years with a range of 11-38 years). Sixty eight patients (40%) were proven to have gallstones in the gallbladder. There was a gender difference (female : male ratio was 1,5:1) and increasing age was associated with an increased prevalence of gallstones.One patient require an incision in the common bile duct to remove multiple stones. Apart from cholelithiasis, other pathological findings in the removed gallbladders were chronic inflammation, cholesterosis and haemosiderosis.

There was no increase in the postoperative complications due to the addition of cholecystectomy and appendicectomy, whilst the mean duration of hospitalization was 11 days. Almost all patients were transfused preoperatively, with the aim to achieve on hematocrit greater than 30%.

In conclusion, strict adherence to a preoperative transfusion regimen, careful anesthesia and meticulous surgery with a close postoperative care insures the best outcome. Elective cholecystectomy is preferred to prevent the complications of cholelithiasis, which may necessitate an emergency operation in an unprepared patient.

**PO124** PANCREATIC PSEUDOCYSTS: OUR EXPERIENCE ON 94 CASES OBSERVED.

D. Marrano, O. Campione, V.M. Greco,
R. Casadei, S. Zuppiroli, L. Torricelli.
Istituto di 1^ Clinica Chirurgica - Bologna

In the 1st Surgical Clinic of the University of Bologna, 94 cases of pancreatic pseudocysts have been observed in the period between 1975 to 1989. Pseudocysts developed during a chronic pancreatitis were 58,5% of cases (53 patients), during a recurrent acute pancreatitis were 38,2% of cases (36 patients), and following a trauma in 3,3% of cases (5 patients).

The surgical operations performed were: a) external drainage in 26 cases; b) internal drainage in 40 cases; c) removal in 28 cases: 8 of which were cystectomy and 20 were cystectomy with pancreatic resection (11 left pancreatectomy with splenectomy, 5 left pancreatectomy with splenectomy and Wirsung duct drainage, 3 duodenocephalopancreatectomy and one subtotal pancreatectomy).

In our experience we prefer: 1) to perfom a radical operation (pseudocyst removal, associated or not with a pancreatic resection) when it is possible; 2) to perform an internal drainage (preferring cystojejunostomy on y-en-Roux loop combined with a pancreatic drainage in the forms resulting from a chronic pancreatitis) when performing radical operation is not safe; 3) an external drainage should be done only when it is necessary.

Recently in our experience these operations have been significantly increased. This is due to more accurate methods of diagnosis using modern imaging techniques (ultrasound, CT-scan). They enable us to have an early diagnosis of pseudocysts in which the wall is not formed yet or still immature.

The immediate and long-term results have been good. Concerning immediate postoperative period only 5 cases of pancreatic fistulae (3 after external drainage) were observed. However these patients had a complete recovery after medical theraphy with somatostatine and total parenteral nutrition.

### P0125 AVAILABILITY OF PREOPERATIVE BILIARY DECOMPRESSION BEFORE EXTENDED RADICAL PANCREATODUODENECTOMY IN JAUNDICED PATIENTS

K.Ueno, T.Nagakawa, N.Kadoya, T.Nakamura, Y.Nakano, H.Kobayashi, K.Maeda, N.Ueda, M.Kayahara, T.Ohta, I.Konishi, T.Matsumoto, I.Miyazaki,
The Second Department of Surgery,
School of Medicine,
Kanazawa University, Kanazawa, Japan

The relationships between the effect of preoperative biliary decompression (PBD) and postoperative complication after pancreatoduodenectomy(PD) were studied. PD were classified into three groups; "extended", "semiextended" and "limited" operations. "Extended" operation involved complete dissection of the regional lymph nodes, extensive removal of retroperitoneal connective tissue and segmental resection of the portal vein. Postoperative complication consisted of anastomotic insufficiency, prolonged renal or hepatic failure, etc. The PBD effect was appraised by serum bilirubin decreasing rate and classified into four grades.

One hundred and twenty two operations of PD in patients with pancreatobiliary carcinomas were performed. These consisted of 59 extended, 31 semiextended and 32 limited operations. 108 patients were treated with PBD. The numbers with excellent, good, fair and poor PBD effects were 17 , 45, 33 and 13 cases respectively. The overall postoperative complication rate was 36.1% (44 patients). In the patients with the poor PBD effect, the complication rate was higher(61.5%) than in those with the excellent effect(17.6%). And the group in the patients undergoing the extended operation with the poor PBD effect had significantly higher complication rate(56.0%) than in those undergoing the limiting operation with the excellent effect(20.2%).

The conclusion was as follows; when the extended radical PD was performed, PBD was necessary. And preoperative serum bilirubin decreasing rate was available index for predicting postoperative complication. So if the PBD effect was poor, the extended operation should not be performed.

### P0126 TAMOXIFEN IN THE TREATMENT OF ADENOCARCINOMA OF THE PANCREAS

R.S. Stubbs, J. Prasad, P. Morum

Department of Surgery, Wellington School of Medicine, Wellington, New Zealand

There is some evidence to suggest human pancreatic adenocarcinoma may be responsive to sex hormone manipulation. Numerous brief reports have appeared indicating a survival advantage for patients with unresected adenocarcinoma of the pancreas treated with tamoxifen 20 mg twice daily. We have treated 13 patients with non-resected carcinoma of the pancreas in this way and have a follow-up of between 8 and 22 months. For each patient we have selected three historical controls matched for age, sex and stage of disease at diagnosis. The median survival of the 39 control patients was 18 weeks, whereas for the tamoxifen treated patients it was 29 weeks. Life table analysis (see figure) suggests initial benefit for tamoxifen treated patients but the difference is not statistically significant (p=0.1).

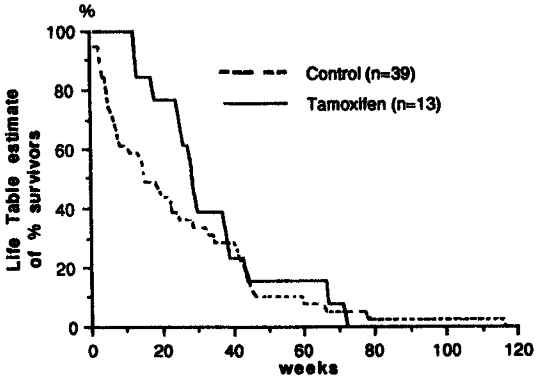

We conclude that while tamoxifen alone may not be of significant value in the treatment of carcinoma of the pancreas, the use of more potent sex hormone inhibitors is worthy of investigation.

### PO127 REGIONAL CHEMOTHERAPY OF COLORECTAL SECONDARIES TO THE LIVER

R. Stangl, A. Altendorf-Hofmann, J. Scheele
Department of Surgery, University Hospital, Erlangen, FRG

From 1977 through 1987, a total of 114 patients with colorectal liver metastases underwent regional chemotherapy using 5-FU either alone or in combination with Mitomycin C. All patients were followed until January 1, 1990, or death.

The overall response rate in patients suitable to be classified according to WHO-criteria was 83% (CR = 14%, PR = 42%, NC = 27%). For the entire series, median and maximum survival from catheter placement were 12,4 and 51 months, respectively. A highly significant influence on Kaplan-Meier estimated survival was found for grading of the primary tumour, hepatomegaly, percentage of liver volume replaced by tumour, elevation of alkaline phosphatase, LDH, and combination of either alkaline phosphatase or bilirubin with hepatomegaly, extrahepatic tumour, and response to treatment.

Significant side-effects were observed in 34,1% of patients. Initial tumour progression addressed the liver in 56 patients, and various extrahepatic sites in 27 patients. In 20 patients tumour progressed simultaneously both in and outside the liver, and in 10 cases the location of initial progression remained unknown. One patient died in complete response from acute gastrointestinal bleeding, which for several reasons is unlikely to be related to treatment.

If compared to a group of 119 patients with a similar stage of disease (no extrahepatic tumour, primary growth completely removed) who remained untreated between 1970 and 1987, regional chemotherapy failed to improve survival significantly. Although these untreated patients consist a historical control group, this result indicates that prognostic benefit from regional chemotherapy needs to be judged against a no-treatment arm in a randomized prospective manner.

P0128 **INTRAOPERATIVE ULTRASONOGRAPHIC STAGING OF EXOCRINE PANCREATIC CANCER.**

**G.Flati, D.Flati, B.Porowska,P.Negro, A.Trecca,C.Zompetta,M.Catarci,M.Carboni**

**V Surgical Unit-II Dept.Surg.-ROME-I**

Although the role of intraoperative ultrasound in treating insulinomas and other endocrine neoplasms (1) has been rather well defined during the last ten years , a little experience has been accumulated with intraoperative ultrasonography (IU) of exocrin pancreatic cancer (EPC). It is generally agreed that only 20% of pzs with EPC are potentially candidates to pancreatic resections. In most of these cases intraoperative decision making is still a major concern. In a consistent number of pzs the real involvment of major vessels cannot be reliably excluded or confirmed by the classic diagnostic means and is indaginously verified by surgical exploration. On July 1988 we started the present prospective study in order to assess the role of ultrasonography in intraoperative staging of EPC.
Fifteen patients (12 males and 4 females, mean age 63.2 y. and 68 y. respectively) underwent laparotomy with preoperative diagnosis of pancreatic cancer. In three patients the tumor was localized in the body tail and in twelve it was localized in the head . Intraoperative ultrasonography (IU) was performed following a standard procedure aimed at defining: 1) size of the tumor, 2) its anatomic relationships with duodenum, CBD , splenic vessels (SV) ,superior mesenteric vessels (SMV) and portal vein (PV), 3) liver morphologic changes, 4) regional linfonodes involvment. According to our protocol neoplastic invasion of portal vein ,splenic vein or major regional arteries represent a contraindication to exeretic surgery. The IU findings allowed us to stratiphy our patients according the following classification:
Grade 1 : Intracapsular tumor< 2 cm (n=1)
Grade 2 : Intracapsular tumor> 2 cm (n=1)
Grade 3 :Invasion of duodenum and/or CBD and/or peripancreatic tissue.(n=5)
Grade 4: Invasion of SV and/or SMV and/or PV and/or adjacent organs or distant methastasis.(n=8)
In 2 out of 5 patients with liver methastasis the preoperative diagnosis was correct. In 3 pzs they were discovered intraoperatively and in 1 of them occult intraparenchimal methastasis were revealed by ultrasound.
In conclusion a routine use of IU in expert hands can help in avoiding useless surgical manouvers in verifing the infiltration of big vessels when the angiographic imaging is doubtful and is practically a valid mean for a rapid and objective intraoperative staging of exocrine pancreatic cancer.
References:1)Klotter H.J.,et Al. Wordl J. Surg. 1987, 11:635-641

### P0129 CORRECTION OF STEATORRHOEA WITH ENZYME SUPPLEMENTATION AND LOW-FAT DIET AFTER SURGICAL SUPPRESSION OF EXOCRINE PANCREATIC FUNCTION

M. Braga, A. Zerbi, M. Cristallo, S. Dal Cin, D. Agape,

C. Bonato and V. Di Carlo - Pat. Chirurgica - IRCCS

San Raffaele - University of Milan, Italy

The occurrence and extent of maldigestion and malnutrition was studied in 14 patients who had undergone pancreatoduodenectomy (PD) with occlusion of the Wirsung duct by Neoprene injection, which results in sclerosis of the acinar pancreatic tissue, but spares the endocrine function. Indications for PD were: chronic pancreatitis (4 cases), pancreatic carcinoma (4 cases), cancer of the papilla of Vater (3 cases), endocrine pancreatic tumor (3 cases).
Digestive function was assessed by D-xylose test and determination of faecal fat excretion; nutritional status was evaluated by monitoring body weight and measuring serum albumin, total iron binding capacity, total lymphocytes count, cholinesterase.
Before discharge patients were put on a 70 g/day dietary fat intake. Faecal fat was 32.91 ± 8.1 g/day without enzyme replacement and fell to 14.21 ± 6.6 g/day with pancrelipase supplementation (16,050 USP Units lipase per meal). D-xylose test was normal in all patients. At discharge all patients were underweight (mean 88.3 % of their usual body weight) and 9 patients showed an alteration of laboratory nutritional parameters.
At time of discharge a diet of 2,400 Kcal/day with a low-fat intake (50 g/day), associated with enzyme supplementation, was prescribed.
Six months after surgery nutritional status and faecal fat excretion were re-evaluated while patients were on low-fat diet. Faecal fat excretion further decreased to 8.32 ± 3.9 g/day ($p < 0.01$ vs before discharge value). All patients but one normalized nutritional parameters, and gained weight reaching 93.0 % of the usual body weight. In particular, six patients reached their preillness usual body weight.
Our data show that the association of enzyme replacement therapy and low-fat diet allows a good correction of steatorrhoea and a significant improvement of nutritional status.

### P0130 BACTERIOLOGY OF BILIARY TRACT DISEASE

M.J.Panovski,N.P.Panovski.Department of Digestive Surgery,Institute of Microbiology,University of Skopje,YU

Infection is a problem in biliary surgery and precautions should be taken to reduce it./Keighley 1988/. A policy for the prophylactic use of antibiotics, based on local knowledge of bile bacteriology is essential.(Gunn 1982/.
A bacteriological study was made of 332 patients undergiong surgery for biliary tract disease. Samples were sollected during operation and were cultured both by aerobic and anaerobic methods. Sensitivity tests were done as well. Analysis /cluster and multivariate/ to determine clinical findings associate with biliary sepsis, was done too.
There was a correlation between the pathoanatomic supstrate and the positive bacteriological finding.

| Diagnoses | | bile bacteriology | | | | |
|---|---|---|---|---|---|---|
| | . | positiv | | Aer. | Anaer. | Mixed |
| | No | No | % | % | % | % |
| Cholecystitis chr. | 162 | 23 | 14.1 | 86,9 | 13.1 | |
| Cholecystitis acuta | 89 | 42 | 47.2 | 83,4 | 4,7 | 11,9 |
| Choledocholithiasis | 43 | 29 | 67,4 | 79,3 | - | 20,7 |
| Malignant stricture | 10 | 6 | 60 | 100 | - | - |
| Post op.stricture | 4 | 4 | 100 | 100 | - | - |
| Cholangitis | 24 | 21 | 87,5 | 71.4 | - | 28,5 |
| Total | 332 | 125 | 37,6 | 82,4 | 4.0 | 13,6 |

Most frequently found bacteria were: E.Coli, Klebsiella Enterobacter, Enterococcus, C.perfringens. Most of the bacteria were sensitive to amoxycillin-Clavulonis acid, piperacillin, or combination of gentamicin and ampicillin.

The authors stand up for selective approach in the anti biotic prophilaxis in biliary surgery.

References

Gunn AA.World J.Surg 1982: 6 : 301
Keighley MBR. British Medical Bulletin 1988 : 44 ; 374

**P0131** CARCINOMA OF AMPULLA OF VATER:A SERIES WITH UNUSUAL LOW SURGICAL RADICALITY AND SURVIVAL

G.C.Pansini F.Buccoliero T.Virgili E.Grandi

G.Carrella M.Rubbini M.Indelli D.Cantarini

General Surgery,Pathology,Oncology,Digestive Endoscopy Institutes - Ferrara University

Italy

A multicentric study from Japan reported in 1977 a 5-year survival rate of 6% for resected ampullary carcinoma,compared with 50% to 62% reported in recent North American and European studies. This discrepancy might be accounted for by difference in tumor pathologic features(tumor grade,size,local invasiveness,degree of differentiation) at time of presentation,as Neoptolemos suggested.

In a series of 11 carcinomas of ampulla of Vater we observed an unusual aggressive disease at time of clinical presentation,with consequent low operability,surgical radicality and survival rates.Of eleven patients with histological diagnosis of ampullary carcinoma only six underwent laparotomy with a view to resection of tumor,at this institution between 1984 & 1989;five patients underwent biliary drainage followed by biliary endoprostheses.
Of six patients who underwent operation,five had pancreaticduodenal resection with no operative mortality;one had palliative by-pass because of undiagnosed locally advanced disease with mets.
What is the influence on survival?
Of five resected patients,two died at 13 and 23-month follow-up; three patients are disease-free at 12,24 and 26-month follow-up respectively;one patient (by-pass proc.) is alive with disease.
Of five non operated patients,one died 2 months after endoprostheses,two resulted lost at follow-up (probably died within 6 months after biliary prostheses),but two patients are still living at 12-months follow-up.

This series shows that ampullary carcinoma can behave with aggressiveness as head-pancreatic cancer and the stage of the disease at time of clinical observation is probably the most determinant prognostic factor in survival prediction.

### PO132 ARTERIAL RECONSTRUCTION USING AN AORTIC GRAFT IN PORCINE LIVER TRANSPLANTATION.

K. Oldhafer, J. Hauss, G. Gubernatis, R. Pichlmayr
Medical School, Hannover,F.R.G.

Orthotopic liver transplantation (OLT) in pigs is a well-established experimental model (Calne 1968). Various techniques have been described for arterial reconstruction. However, in order to investigate haemodynamics and microcirculation after OLT, a standardized arterial reconstruction is required. Aortic anastomoses between the diaphragm and the coeliac artery are often associated with two complications: pneumothorax and injury to the lymphatic sac. The purpose of this investigation was to study the haemodynamics and usefulness of aortic grafts for arterial reconstruction.

Methods. OLT was performed in 10 pigs weighing 18-25 kg. 10-15 cm length of aorta was removed together with the coeliac artery: in 7 cases a proximal part (thoraxic aorta) and in 3 cases a distal part (infrarenal aorta) was used. The aortic graft near the coeliac artery was prepared to a funnel-shaped form. The infrarenal aorta just below the left renal vein was used for anastomosis in the recipient. The hepatic arterial flow was measured in the donor and 1 hour after reperfusion in the recipient using an electromagnetic flowmeter (Cliniflow II, Model FM701D).

Results. The access to the infrarenal aorta was uncomplicated. No pneumothorax and no lymphatic fistula were observed after OLT. The flow of the hepatic artery was 140.0 $\pm$32.2 ml/min in the donor and 200.0 $\pm$69.2 ml/min after reperfusion. Thrombosis of the hepatic artery was observed only in 1 case.

Conclusion. The infrarenal aorto-aortic anastomosis using an aortic graft proved to be a reliable and standardized technique for arterial reconstruction in porcine OLT.

References.

Calne RY, et al. Brit J Surg 1968; 55: 203

### P0133 OXYGEN CONSUMPTION DURING AND AFTER LIVER TRANSPLANTATION

CW Pinson, MD*, JW Ragsdale, MD*, CW Deveney, MD*, RL Jenkins, MD**. *The Oregon Health Sciences University, Portland, OR, USA. **New England Deaconess Hospital, Boston, MA, USA.

Liver transplantation provides an opportunity to evaluate how the diseased and grafted liver influences total body metabolism as measured by oxygen consumption. Oxygen consumption was measured in 14 patients undergoing liver transplantation. Cardiac output, hemoglobin, and paired arterial and mixed venous blood samples were obtained in the pre-anhepatic phase, anhepatic phase, after portal reperfusion, after hepatic artery reperfusion, and postoperatively. Oxygen consumption was calculated by the Fick equation and normalized to an oxygen consumption index ($VO_2I$ in ml/min/$M^2$).

Baseline, or pre-anhepatic $VO_2I$ was 95 ± 21. Anhepatic $VO_2I$ was 76 ± 19, a 20% decline ($p < 0.05$). Portal revascularization of the allograft liver increased $VO_2I$ to 106 ± 23, a 10% increase from baseline and 28% increase from the anhepatic phase. After hepatic artery revascularization, $VO_2I$ rose to 114 ± 28, a 17% ($p < 0.05$) rise over baseline and 33% ($p < 0.05$) rise from the anhepatic phase. Postoperatively, the $VO_2I$ rose to 150 ± 11 at 24 hours, a rise of 37% ($p < 0.05$) from baseline and 49% ($p < 0.05$) from the anhepatic phase.

This study demonstrates that the end-stage liver influences 20% of the total body oxygen consumption. The incremental increase in total body oxygen consumption was 17% after hepatic artery revascularization and 37% postoperatively, representing a relative total body hypermetabolism with the newly grafted liver compared to the diseased liver. These observations are important in understanding the altered physiology surrounding liver transplantation.

P0134 THE CHOLANGIO-DUODENAL INTERPOSITION OF A JEJUNAL SEGMENT AFTER RESECTION OF PROXIMAL AND CENTRAL BILE DUCT TUMORS

B.Kremer, D.Henne-Bruns, *H.Grimm
Dep. of Surgery and *Surgical Endoscopy; Univ. of Hamburg; FRG

From Jan. 1988 to Dec. 1989 20 patients with proximal and central bile duct carcinomas were operated in our department. In 15 patients the tumor was resected by removal of the biliary bifurcation, in 7 cases combined with a left, right or central hepatic resection. In 2 patients a transhepatic drainage of the anastomosis was additionally placed for postoperative "afterloading" therapy.
In 3 patients only a transtumorous/transhepatic catheter was introduced for postoperative intraluminal irradiation because of irresectability of the tumor.
In 2 patients an explorative laparotomy was performed followed by liver transplantation in one case.
Lethality of all procedures was 15% and of the resected patients 6.6%.
Because of the high incidence of tumor recurrency in this typ of carcinoma even in curative resections, in 7 patients the bilio-intestinal reconstruction has been performed by cholangio-duodenal interposition of a 20 cm jejunal segment.
In contrast to the Roux-en-Y technique this procedure allowes an endoscopic follow-up investigation and a local intervention like laser resection or pigtail drainage in cases of tumor recurrency.

### P0135 RESULTS OF BILIARY RECONSTRUCTION AFTER ORTHOTOPIC LIVER TRANSPLANTATION (OLT)

Lüsebrink R., Raakow R., Lefebre B., Neuhaus P.

Earlier,the biliary anastomosis has been termed the "Achilles heel" of liver transplantation. Rates of biliary complications in OLT were reported with a frequency of 15 to 25% even in recent literature, thus causing major morbidity and mortality. In experimental and clinical OLT it could be shown that side-to-side anastomosis carries the lowest risk of bile leakage or late stenosis.We investigated results with routine side-to-side biliary anastomosis in a new clinical programme of OLT.

From october 1988 until december 1989 we performed 61 OLT in 57 patients. In two cases of HBsAg positive recipients HBV hepatitis led to graft failure 5 resp. 6 month after first OLT,so retransplantation had to be carried out.
In 4 patients roux-en-y choledocho-jejunostomy was chosen because of the recipients underlying disease (2 PSC,2 central cholangiocarcinoma).
In 53 patients side-to-side anastomosis was used for reconstruction of the biliary tract with broad adaptation of both common bile ducts with a 5-0 with running suture. The same technique was used in the cases of retransplantation. The anastomosis was stented with a T-tube which was left for at least 5 weeks before removal. T-tube cholangiography was performed before clamping and before removal of the T-tube.
Currently 53 patients are alive with a follow up of 1 to 14 months. No biliary complications such as breakdown of the anastomosis or mechanical obstruction occured. One patient developed a subhepatic biloma on the 3.p.o.d.,however repeated T-tube cholangiograms and HIDA-scans could not demonstrate anastomotic leakage. The biloma vanished after percutaneous catheter drainage within 3 days,so this biliary leakage was thought to be due to an abberrant bile duct within the gallbladder fossa.
In virtually all cases a mild rise in cholestatic parameters (aP,j-GT) without elevation of serum-bilirubin was observed 2 to 3 days after clamping of the T-tube,which was always reservible.

Since no biliary anastomotic complications were observed since the start of our programm we will continue to use side-to-side anastomosis of the common bile duct as the method of choice for biliary reconstruction in OLT.

**P0136** COMPLICATIONS OF SENGSTAKEN-BLAKEMORE TUBE TAMPONADE OF ESOPHAGEAL VARICES

K.Zieniewicz, B.Michalowicz, J.Pawlak, P.Malkowski, J.Szczerban

Dept.of General Surgery & Liver Diseases
Institute of Surgery, Medical Academy
Warsaw, POLAND

Sengstaken-Blakemore tube tamponade (S-B.T.) was introduced to clinical practice in 1950. Together with IV infusion of vasopressin is now essential and widely used conservative treatment of massive bleeding from esophageal varices. Correctly applied it allows to control the hemorrhage in about 80% of cases.

We analyzed 502 patients treated in 1980-1989, bleeding acutely from esophageal varices in course of liver cirrhosis or portal vein thrombosis . The source of bleeding was established endoscopically. The S-B.T. preceded endoscopic sclerotherapy, shunting procedure or esophageal staple transection. The aim of the study was to estimate the complications of S-B.T.

1. ischemic areas of mucosa of esophagus and/or cardia were found endoscopically in 442 pts(88%); necrotic changes being the source of recurrent fatal bleeding - 25 pts (5%), or esophageal strictures causing prolonged severe dysphagia - 3 pts (0,6%).
2. aspiration pneumonia - caused by blood or saliva aspiration from above the esophageal baloon - 9 pts (1,8%).
3. acute asphyxia caused by esophageal baloon displacement - 1 patient (0,2%).

Conclusion: S-B.T. lasting longer than 12-16 hours threatens with necrotic changes of esophageal or cardial mucosa, that in turn complicates postoperative period after endoscopic sclerotherapy or esophageal staple transection.

**P0137** THERAPEUTIC PROTOCOL WITH SOMATOSTATINE IN PANCREATIC SURGERY

M. Moreno Azcoita; L. Sanchez Urdazpal; J.M. Jover; J.L. Ramos; J. Alvarez; P. Quijano.

Hospital Central Cruz Roja. Madrid. Spain.

Pancreatic surgery has a high index of postoperative complications due, in most cases, to pancreatic fistula that has been reported in 7-20% with an average of 15%.

Somatostatine (SS) has proved its effectiveness to diminish digestive secretions at any level and has been checked in digestive tract fistula. Due to its efectiveness in this particular field, in 1987 we started a protocol in order to prevent pancreatic fistula after pancreatic surgery.

In the protocol the patients included were those with pancreatic resections bigger than 40% and those with pancreato-jejunal anastomosis. Those patients with insulin-dependent diabetes and severe renal failure were excluded.

PATIENTS AND METHODS. Up to now, 26 patients have fulfilled the criteria and have been included in it . The cases were as follows

| | |
|---|---|
| Cefalic duodeno-pancreatectomy | 5 |
| Left resection: in total gastrectomy | 11 |
| Left resection in pancreatic tumour | 4 |
| Pancreato-jejunal anastomosis | 6 |

SS was administered at a dose of 250 mcg/h. for 5 days.

RESULTS. Average age in this group was 60,3 y. (20-83).

Morbidity appears in 3 cases (11,5%) (1 colonic fistula because of drainage tube; one with pneumonia and acalculous cholecystits treated with percutaneous cholecystostomy) and the other with pancreatic fistula lasting 7 days (3,8%).

Operative mortality was 3,8% (1 c.) in a female patient 71 y.o. operated on because of gastric carcinoma. 15 days before this operation she was submitted to aortoiliac bypass and she died necause of pulmonary thromboembolism.

None of our cases shows any complications that could be attributed to pancreatic resection or anastomosis.

CONCLUSION. SS can be satisfactorily employed in every case of pancreatic surgery because it seems to diminish morbidity caused by pancreatic resection or anastomosis.

### P0138 HYDATID DISEASE OF THE LIVER: CONSERVATIVE OR RADICAL SURGICALTREATMENT?

SKALTSAS S., HADJIMARKOU A., BLIOURAS N., FERETIS C., DARAS B., KEKIS B.

GREEK CHURCH HOSPITAL-ATHENS MEDICAL CENTER GR.

Hydatid disease is common in Greece, with approx, twelve hundred new cases reported annually. The Liver is involved in about 60% of the cases.

Surgery is mainly performed whenever serious complications such as Infection, Suppuration and Intra-Biliary or Intra-Abdominal rupture occur.

Conservative Surgery as a partial Cystectomy with Omentopexy or external drainage with or without marsupialization is mainly done, while more radical procedure such as total cystectomy or segmental hepatic resection are rarely performed.

A total of 117 Patients with Liver Hydated disease were operated in our Department during the period 1972-1989. Sixty-eight of these cases presented complications, out of which 25 were more severe. Eighty-eight of the patients underwent conservative surgery with a mean hospitalization time of $37 \pm 4$ days. Postoperative complications were observed in 18 of them.

Radical operations were performed in the remaining 29 and severe postoperative complications were mentioned in three patients while the hospitalization time was $17 \pm 2$ days. There was no operative mortality in any of the groups. Most of the radical operations were performed from 1981-1989.

Decision on conservative or radical surgery was based not upon cyst complications but on the patient's general condition, the size and location of the cyst. Shorter time of hospitalization as well as decrease of the post-operative comlications have been the advantages for deciding on a radical surgery for hydatid disease of the liver.

P0139 EXPERIENCE OF 113 OPERATIONS FOR HEPATOCELLULAR CARCINOMA IN A WESTERN COUNTRY. A MORPHOLOGICAL ANALYSIS

N.Nicoli, R.Colombari*, L.Marchiori, G. Mangiante, M.Mainente, V.Costa, A.Asnicar, E.Piazzola*, F.G.Musajo,F.Capra**, M. Casaril**, A.Dagradi.
Department of Surgery, Pathology*, Internal Medicine**, University of Verona, Italy.

Hepatocellular carcinoma(HCC), even quite rare in western country, can be now detected in pre-clinical stage using US and alfa-fetoprotein level assesment in at risk subjects. Surgical resection is the only curative treatment.
Between I970 to I989 we surgically treated II3 HCC, 44 in normal liver and 69 in cirrhotic liver. Each tumor was studied with a picture of cut surface and microscopically with at least 5 blocks from different areas of neoplasia. We evaluated dimension, growth pattern(Okuda 1984), capsule, grading of differentiation(Edmonson I954) and microscopic architecture(Gibson I978). The capsule,when present, was frequently infiltrated by neoplastic cells. Vascular invasion has been observed not only in larger tumor but also in small HCC. Regarding to differentiation,in the same tumor we frequently saw a mixture of well, moderate or poorly differentiated areas. Only in 27 cases we could find a pure type of architecture, in all other cases being mixed type.
In consequence of this wide variety of histological pattern we cannot distinguish reliable classes of prognosis based just on morphological data.

Reference:
Okuda K, Peters RL, Simson IW. Cancer I984;54:2165.
Edmonson HA, Steiner PE. Cancer I954;7:462.
Gibson JH. WHO n.2O, I978.

### PO140 ULTRASOUND GUIDED PERCUTANEOUS TRANSHEPATIC CHOLANGIOGRAPHY AND DRAINAGE IN PATIENTS WITH HILAR CHOLANGIOCARCINOMA

JS Laméris, EJ Hesselink, PA van Leeuwen, HGT Nijs and OT Terpstra. Dep of Radiol and Surg. University Hospital Rotterdam, NL

The use of ultrasound guided percutanous transhepatic cholangiography and drainage(PTCD)in 56 patients with hilar cholangiocarcinoma was evaluated. In 12 patients PTCD was performed as a preoperative measure either to outline tumour extension or to treat cholangitis. Postoperatively the catheters were used to guide Iridium 192 for radiotherapy in 9 patients with irresectable tumour or tumour residue. In 23 inoperable patients with tumour diameter smaller than 3 cm and in whom at least one catheter could be manipulated through the tumour, PTCD was combined with in- and external radiotherapy. The remaining 21 patients were palliated with endoprostheses and catheters only. Complete drainage of the bilairy system was achieved in 35 patients(62%). The majority of these(33) had an internal drainage by means of endoprostheses, two had a combination of an endoprosthesis and an external catheter drainage. Of the 21 patients(38%) with in- complete drainage 13 had endoprostheses, 4 a catheter and an prosthesis and 4 external catheter drainage. Procedure related complications occured in 11 patients. In 10 these complications could be classified as minor, requiring only conservative measures. In one patient with ascitic fluid and severe cholangitis PTCD caused bacterial peritonitis of which the patient subsequently died. The median survival of patients with PTCD only was 4 months. A significant increase of survival was seen in patients treated with PTCD and radiotherapy. (median survival 8 months). No significant difference between the surgical treated and the radiotherapy group could be shown.
We conclude that ultrasound guided PTCD, being a safe technique, should have a place in the treatment of patients with hilar cholangiocarcinoma.

**P0141** SURGICAL OUTCOME IN PATIENTS CONSIDERED CANDIDATES FOR RESECTIONAL PANCREATIC SURGERY

D.J. van Leeuwen, L. Th. de Wit, A.H.M. Molenaar, K. Huibregtse, M.N. van der Heyde. Hepatopancreatico-biliary Unit, Academic Medical Center, Meibergdreef 9, 1105 AZ Amsterdam-NL

Pre-operative assessment for resectability of pancreatic head carcinoma (PC) is made mostly with imaging studies. We were interested in the **surgical outcome of "resectable" patients with PC who were referred to a tertiary surgical center** (AMC).
108 consecutive referral patients were followed prospectively in 1987 and 1988. Further selected evaluations with endosonography, angiography and review of previous studies were done at AMC before surgical decisions were made.
**Results.** 38 patients (35%) were eventually deemed not suitable for a laparotomy because of premature death (n=1), major vascular involvement (16), tumor diameter of >5 cm (2), refusal of further treatment (5), distant metastasis (5), general deterioration (2), change of diagnosis (8) (stones/pancreatitis-5 lymphoma-1; abscess-2; ) and bypass procedure performed elsewhere (1). Of the 70 (65%) who were operated on, only 41 were suitable for the Whipple procedure with 2 requiring additional portal vein reconstruction. In 18 patients, only bypass procedures were done because of surprise operative findings of major vascular involvement (11) or distant metastases (7). 3 required total pancreatectomy because of portal vein tumor ingrowth (1) or potential anastomotic difficulty (2). 5 underwent other planned non-resective surgery. 2 were found to have benign disease and did not undergo any resection.
**Conclusions:**1) 1/3 of all "resectable" PC patients will be excluded from surgery in a tertiary center. 2) In spite of extensive and high quality pre-operative imaging, resectability remains difficult to predict. 3) Laparotomy for palliation or resection should not be withheld from the willing patients who have equivocal imaging studies.

### PO142 A CYTOLOGICAL STUDY OF PANCREATIC ENDOCRINE TUMOURS.

AB AKOSA, LA DESA, IS BENJAMIN,
T KRAUSZ. .R.P.M.S. London.

The significance of fine needle aspirates in the diagnosis of endocrine tumours of the pancreas has not been extensively studied. Few cases have been reported in the literature.
The relevance of early diagnosis and treatment of pancreatic tumours and the need to distinguish the two main types of tumours, exocrine and endocrine requires that proper cytological criteria be laid down to facilitate this distinction.
We examined 10 cases of cytologically diagnosed endocrine tumours of the pancreas with the view to establishing a morphological criteria for diagnosis.
The presenting symptoms were weight loss (n=9), pain (n=6), jaundice (n=3) and diarrhoea and dizziness (n=1)
Hepatomegaly (n=4), abdominal mass (n=3) and peripheral lymphadenopathy (n=2) were noted.
5 samples were obtained pre-operatively and 5 intra-operatively.
The aspirates were cellular with numerous small clusters and individual cells.
Monotony of the cells with abundant granular cytoplasm was observed. The nuclei were small and eccentric with a ' salt and pepper' chromatin pattern and a small nucleoli.
Immunocytochemically, the tumour cells expressed general neuroendocrine markers and the presence of peptide hormones were also demonstrated.
On review, one of the cases failed to meet the criteria and the histology revealed a well differentiated adenocarcinoma.
The use of these criteria should enable recognition of this inportant subgroup of pancreatic tumours and help to direct appropriate management.Immuncytochemistry used as an adjunct is helpful in further characterisation of the tumour.

### P0143 RHABDOMYOSARCOMA OF THE BILIARY TREE: A REVIEW OF 4 CASES

N.D. Heaton and E.R. Howard.
King's College Hospital, London. UK.

This rare tumour comprises 0.8% of all rhabdomyosarcomas in childhood and the patients usually die within 12 months. Treatment with intensive chemotherapy may lead to long-term survival.

Patients

Four children (3 male, 1 female), mean age three years, presented with obstructed jaundice. A cystic condition of the bile duct was diagnosed on ultrasound scans in three and the correct diagnosis was made in one. The correct diagnosis was made at laparotomy in all 4 cases and confirmed by frozen section. All four had incomplete resection margins on histology.

Treatment

All four underwent surgery for diagnosis and to achieve biliary drainage. Three patients had resection of the extra-hepatic biliary tree and a choledocho-jejunostomy using a roux loop and one underwent a tumour debulking operation and insertion of a T-tube. Three children were treated with chemotherapy. One died at one year from recurrence; one had an extended survival to six and a half, dying of extra-hepatic disease. The third case is alive and well without recurrence fifteen months after surgery. The infant who did not receive chemotherapy died three months after surgery.

Conclusion

Two of our four cases have had prolonged survival after surgery and chemotherapy for this aggressive tumour.

### P0144 INDIUM LABELLED LEUCOCYTE IMAGING IN ACUTE PANCREATITIS

C Hall, M C Winslet, G Sagar

A Calbreath, S R Hesslewood, L K Harding

J P Neoptolemos

Dudley Road Hospital, Birmingham

England

An abnormal indium labelled leucocyte (In 111) image is said to be a useful predictor of those who will develop complications of acute pancreatitis (1). We have performed In 111 imaging, ultrasonography and a prognostic score on patients admitted with a clinical and biochemical diagnosis of acute pancreatitis, and compared the results.

50 patients (30 male, 20 female) were studied. The aetiology was gallstones in 23, alcohol in 20 and unknown in 7. Gallstones were detected by ultrasonography in 22 patients, but the pancreas was only visualised in 37 patients and was considered abnormal in 13. This confirms previous work that shows ultrasonography is excellent at determining aetiology, but poor at predicting severity (2).

Prognostic scoring was completed within 12 hours of admission. 11 were predicted severe, but only one of these went on to become clinically severe, possibly due to treatment protocols.

Clinically, 6 patients had a severe outcome (2 respiratory complications, 2 pseudocysts, 1 pancreatic ascites, 1 death). 4 of these had an abnormal In 111 scan and there was one equivocal result, a sensitivity of 67%. Of the 44 patients with a mild outcome, 24 had normal In 111 images giving a specificity of 55%.

The positive predictive value of In 111 imaging is only 21%. We conclude that this limits its usefulness as a predictor of clinical outcome despite the sensitivity of 75%.

(1) Anderson J R et al. J Nucl. Med. 1986; 27: 345-352
(2) McKay A J et al. Br J Surg. 1982; 69: 369-372

### PO145 DETECTION OF LIVER METASTASES IN COLORECTAL CANCER: A PROSPECTIVE STUDY

K.-G. Tranberg, B. Ohlsson, C. Lundstedt, H. Ekberg, E. Hederström. Department of Surgery and Roentgenology, Lund University, Lund, Sweden.

The aim of this prospective study was to determine the value of imaging (ultrasound (US), computed tomography (CT), angiography, arterially enhanced CT (CTA), biochemical analysis (liver function tests, carcinoembryonic antigen (CEA)) and intraoperative surgical evaluation for the detection of colorectal liver metastases.

Between 1984 and 1986 71 patients without clinical evidence of liver metastases were investigated with angiography, US, CT and CTA of the liver within 2 weeks before surgery of colorectal cancer. Intraoperative surgical evaluation was standardized. The clinical follow-up lasted for 3.5-5.5 (median 4.5) years and included regular determinations of liver function tests and CEA and repeat CT and US at 1 year after operation in patients without proven metastases. If there was evidence of liver tumour at the repeat imaging or at any time during the clinical follow-up, it was assumed that tumour was present at the time of colorectal surgery.

The accuracy of surgical evaluation, angiography, US, CT and CTA was 92, 77, 77, 83 and 85 %, respectively. Corresponding predictive values for a positive test were 100, 93, 92, 89 and 83 %, whereas predictive values for a negative test were 88, 74, 74, 81 and 85 %, respectively. Preoperative determination of liver function tests were not helpful in detecting liver metastases. Preoperative CEA was also of limited value (accuracy 70-80 % depending on cut-off level). Accuracy or predictive values were not improved by combinations of tests. 22 patients (31 %) had synchronous liver metastases, and another 4 patients (6 %) developed metachronous liver metastases after 1-3 years. 8 of the 22 patients with synchronous metastases, and none of the 4 patients with metachronous disease, underwent liver resection.

It is concluded that US, CT and CTA are rather accurate in detecting liver metastases. However, none of them is good enough to merit its routine use before colorectal surgery, partly because of the accuracy of the surgical evaluation. They are potentially vaulable for follow-up if used at frequent intervals and not only when prompted by clinical or laboratory examination.

### P0146 A New Nonoperative Method of Percutaneous Transgastric Fistulo-drainage for An Intractable External Pancreatic Fistula

T.Ohta, T.Nagakawa, N.Kadoya, H.Kobayashi, Y.Nakano, T.Nakamura, N.Ueda, K.Maeda, M.Kayahara, K.Ueno, I.Konishi, T.Matsumoto, I.Miyazaki
The Second Department of Surgery, School of Medicine, Kanazawa University, Kanazawa, Japan.

External pancreatic fistula is a well-known complication of pancreatectomy and is conventionally classified, according to the volume of the output, as a partial fistula (less than 200 ml per day) and a total fistula (more than 200 ml per day). A partial fistula usually closes spontaneously, and operative intervention is rarely needed. On the other hand, a total fistula often has little tendency to close and therefore may require surgical intervention. Herein we report a case of an intractable total pancreatic fistula following pancreatoduodenectomy which was successfully cured by a new nonoperative method of percutaneous transgastric fistulo-drainage(PTFD). This technique under the control of two-way x-ray television system is safe and is considered to be useful for the treatment of an intractable total pancreatic fistula. In the future, this may be the management of choice for such refractory total pancreatic fistula. The detailed procedure is introduced below.

Procedure of PTFD: A percutaneous endoscopic gastrostomy was created into the left hypochondrium using a Sacks-Vine gastrostomy kit. 3 weeks after the gastrostomy, 19-gauge intravenous cannula was thrust across the gastrostomy site and posterior gastric wall into the pancreatic fistulous tract under the control of two-way x-ray television system. After the cannula was confirmed to be present in the pancreatic fistulous tract by injecting contrast medium, a flexible guide wire was passed through the cannula into the pancreatic fistulous tract. The guide wire was then pushed through the pancreatic fistulous tract to the orifice of the body surface. A 10-French drainage catheter with a few lateral foramina was inserted back along the guide wire to establish PTFD. On about 30th day after this procedure, PTFD catheter was removed and an endoprosthesis (a double mushroom type catheter with a few lateral foramina) was inserted from the orifice of the pancreatic fistula into the stomach to convert an external drainage to an internal drainage.

### P0147 POLYMORPHONUCLEAR COUNT IN ASCITIC FLUID AFTER LAPAROTOMY IN CIRRHOTIC PATIENTS.

C. Vons, E. Faivre, R. Chiche, J. Belghiti
Service de Chirurgie HI Beaujon 92118 Clichy France

Polymorphonuclear (PN) count greater than 250/mm$^3$ in ascitic fluid is the most important criteria of diagnosis of bacterial peritonitis in cirrhotic. After a laparotomy the value of PN count has been questioned. The aim of this work was to evaluate ascitic fluid PN count to diagnosis postoperative bacterial peritonitis. A prospective study was undertaken in 14 patients with cirrhosis and ascites undergoing hepatectomy (n=4), portocaval shunt (n=4) and biliary and digestive surgery (n=6). Fifty-six consecutive specimens of ascitic fluid were obtained by paracenthesis or through abdominal one-way suction tubes left in place. In 6 cases, ascitic fluid was blood stained and PN count was unreliable; none of these specimens demonstrated positive ascitic fluid culture. In the remaining 50 specimens PN count ranged from 5 to 5920/mm$^3$. PN count lower or greater than 250/mm$^3$ was accorded to the results of ascitic fluid culture. Results were as follows :

| | PN count < 250/mm3 (n=38) | PN count > 250/mm3 (n=12) |
|---|---|---|
| Positive ascitic fluid culture (n=6) | 2 (5%) | 4 (33%)* |

* $p<0.05$

These results suggest that, as in non operated cirrhotic patients : (a) PN count should be taken in account in the diagnosis of postoperative bacterial peritonitis; (b) PN count greater than 250/mm3 is a good criteria for the diagnosis of bacterial postoperative peritonitis.

### P0148 POSTVAGOTOMY CHANGES OF THE CANINE BILIARY TRACT PRESSURE

M. Tezuka, H. Kogure, K. Monma, A. Kadowaki, N. Satoh, Y. Tajima
Department of Surgery, Dokkyo University School of Medicine, Tochigi, Japan

There has been a considerable divergency of opinion as to the pathogenesis of an increased incidence of postvagotomy cholelithiasis. This experimental study was undertaken to clarify the relationship of cholestasis to the changes of pressure of the biliary tract after vagotomy using dogs.

Gallbladder pressure was directly measured by cathetertip pressure transducer before and after vagotomy. In contrast to no changes in the non-vagotomized dogs, three types of changes were noticed in the vagotomized dogs: rise of the pressure, decline of the pressure and no change. The pressure showed a tendency to decrease in the vagotomized cystic duct ligated dogs. This might imply the gallbladder pressure is controlled by the inflow of hepatic bile from the common bile duct.

The wave patterns of the internal pressure of the duodenal papilla were analyzed in respect to three parameters: maximum pressure, minimum pressure and frequency(cycle/5min.). In the vagotomized group, the maximum pressure was markedly reduced following vagotomy. The wave patterns of the perfusion pressure of the common bile duct following vagotomy were separately assessed at the base line and the peak pressure. Following vagotomy, the perfusion pressure at the base line showed no significant change. The peak perfusion pressure, however, significantly decreased after vagotomy, reflecting the decreased papillary contractility.

In conclusion, our data support the view that dysfunction of papillary contractility after vagotomy might be the possible cause of cholestasis.

P0149 TOLERANCE TO TOTAL PARENTERAL NUTRITION IN CHINESE PATIENTS WITH SEVERE ACUTE PANCREATITIS

C.O. Mok, S. Paterson-Brown, R.D. Mitchell, W.W.K. King, A.K.C. Li
Department of Surgery, Prince of Wales Hospital, The Chinese University of Hong Kong, Hong Kong

Over a two years period, sixteen Chinese patients ( 9 males, 7 females, mean age 55 years ) with severe acute pancreatitis were given total parental nutrition (TPN) for a mean duration of 22 days ( range 4-100 days ). A carbohydrate-based TPN providing 1.5 to 1.75 x the basal energy expenditure was given. Fat emulsion was also administered to 7 patients requiring long term TPN to avoid essential fatty acid deficiency. Their serum amylase, triglyceride and cholesterol levels remained unchanged. Nutritional efficacy was achieved in all sixteen patients by the maintainance of the mean body weight ( pre-TPN 60.4 kg and post-TPN 60 kg ) and the improvement of the mean serum albumin level from 28 g/l ( range 21-40 ) pre-TPN to 33 g/l ( range 26-42 ) post-TPN. There was no impairment of their liver function as measured by the serum bilirubin, alkaline phosphatase and alanine aminotransferase level. Two of five patients requiring insulin for blood glucose control developed transient hyperosmolar nonketotic coma. Two patients developed catheter sepsis within 2 weeks of TPN. Overall, twelve patients were discharged while four patients died from complications of pancreatitis. We conclude that a carbohydrate-based TPN can provide adequate nutritional support with low morbidity to patients with severe acute pancreatitis. Furthermore, the use of fat emulsion as supplemental energy substrate and source of essential fatty acid is well tolerated in this group of patients with pancreatitis.

### P0150 CHOLEDOCHAL CYST IN ADULT LIFE

**G.Belli, A.Monaco, A.Daboval, M.F.Armellino, A.D'Agostino**; University of Naples; II^Faculty of Medicine and Surgery; General Surgery and Organ Transplantations; ITALY

Choledochal cyst is a rare congenital condition with an incidence of one in thirteen thousand live births(1).However, in nearly 20% of patients with bile duct cysts, the diagnosis is delayed until adulthood(2). At this stage, because of the frequent association with related hepatobiliary pathology,a more complex and challanging situation will result. The authors show their experience in two cases of choledochal cyst in adult life observed in the last 2 years. Both patients had a type I Cyst according to "Todani Classification".The first case,a 24 years old man drug abuser,was admitted for intensive jaundice and,although preoperative US, TC and ERCP showed a type I choledochal cyst,a PTC was necessary to show the whole hepatobiliary anatomy. The second patient, a 55 years old man,operated on for post-pancreatitis fluid collection was discovered having a type I choledochal cyst with an anomalous junction of the distal common bile and pancreatic duct as showed by an intraoperative cholangiopancreatography. Both patients were treated with cholecystectomy and cystic resection plus hepaticojejunostomy by Roux-en-Y jejunum loop (histology was negative for malignancy).The postoperative course was uneventfull and both patients remain well and symptoms free with normal liver function tests at two years and three months follow-up,respectively.

Although the surgical management of adults bile duct cysts is controversial, we believe that the treatment of choice is total cystectomy and Roux-en-Y hepaticojejunostomy particularly for type I.The theoretical advantages of this approach include a reduced incidence of anastomotic strictures, stone formation, cholangitis and intracystic malignancy.

1)Flanigan D.P. "Biliary cysts"Annans Surg.1975,182:635-643

2)Nagorney D.M. and Coll."Choledochal cysts in adults:Clinical management" Surgery 1984, 96:656-663

**P0151** COMPLICATIONS IN ARTERIAL ANASTOMOSIS IN LIVER TRANSPLANTATION

E. MORENO; I. GARCIA; I. GONZALEZ-P; R. GOMEZ; H. BONET; C. LOINAZ; J. BERCEDO; P. VORWALD; E. CUARESMA.

Hospital 12 de Octubre. Madrid. Spain

During 1986-1989 in the Servicio Cirugía de Aparato Digestivo II of Hospital 12 de Octubre (Madrid, Spain) carried out 108 Liver transplant (LOT) in 91 patients which 75 were adults and 16 children.
MATERIAL AND METHODS: The arterial thrombosis (AT) performed were as follows:

| GROUP | DONOR | RECIPIENT | PATIENTS | % |
|---|---|---|---|---|
| A | Celiac-Axis | Hepatic artery | 65 | 60,1 |
| B | Celiac-Axis | Celiac-Axis | 22 | 20,3 |
| C | Celiac-Axis | Splenic-artery | 8 | 7,4 |
| D | Hepatic artery | Others | 6 | 5,5 |
| E | Double Anastomosis | | 6 | 5,5 |

11 Patients (10,18%); 9 adults(8,3%) and 2 children(1,85%) showed hepatic artery thrombosis (HAT): Group A: 7 cases; B: 1; C: 1; D: 1 and E: 1.
The clinical presentation follows three patterns:
1: Acute Hepatic failure: 3 cases, day appearance rate: 4,3.
2: Relapsing Bacteremia : 7 cases, day appearance rate: 22,5
3: Delayed Biliary Leak: 1 case, day appearance rate: 55
Diagnosis was made by HIDA TM99, ultrasound and angiogram.
In 7 cases were performed retransplant (Groups A: 4 cases; B: 1; C: 1; and D: 1) In two cases the AT was treated with catheterization plus urokinase injection.
RESULTS: After retransplantation, 5 patinets showed well general condition. The other 2 patients presentd primay graft failure and was impossible to find a donor to carry out a third L.T. In 2 cases the AT was treated by catheterization and urokinase, presenting good results mortality was 36,3% in adults and it was due to delay in finding donors to make retransplant. There was no child mortality.

### P0152 A SIMPLIFIED ONE STAGE MODIFICATION OF PORTOAZYGOS DISCONNECTION FOR MASSIVE VARICEAL HEMORRHAGE

J. Manny, E.M., Luttwak, A Rivkind, Z. Eyal
Hadassah Medical Center, Jerusalem, Israel

A one stage method of portoazygos disconnection is described which controls three main pathways of increased portal pressure to gastroesophageal varices: 1, the short gastric vessels; 2,the coronary vein; 3,transmural gastric communicants. This procedure is performed through an abdominal approach, is technically simpler than other methods and seems to achieve comparable devascularization with favorable results.

The abdomen is entered through an upper midline incision extending from the xyphoid process to 3 cm. below the umbilicus. The spleen is mobilized and removed. The stomach is rotated medially and the coronary vein identified. The vein is then doubly ligated and transected at its emergence, posterior to the upper border of the pancreas. The lesser omentum is entered above the crow foot of the nerve of Latarget and devascularization of the lesser curvature performed, carefully avoiding any injury to the ascending branch of the left gastric artery. At this stage, the stomach is stapled across its upper part and the stapler line is enforced with interlocked continuous running sutures using 00 Prolene. A side to side anterior gastrogastric anastomosis, no less than 3 cm., is made in order to prevent emptying disturbance of the pouch. A second row of Lembert sutures of 000 silk is placed anteriorly and posteriorly, covering the line of staplers.

11 patients, age 35-55, with cirrhosis of the liver, were operated upon during active variceal bleeding. All the patients survived. During a follow up period of 6-9 years, minor rebleeding developed in 2 patients and was easily controled by sclerotherapy. This procedure seems to achieve long term control of variceal bleeding comparable to shunting procedures. Portoazygos disconnection has several advantages: 1, it is not associated with further compromise of hepatic portal inflow, avoiding additional insult to the already failing liver. 2, many patients have hypersplenism, splenectomy has a prompt beneficial effect on the hematologic status. Obliteration of all transmural communications by complete tranverse stapling of the stomach is mandatory.

Gastric partitioning in morbid obesity shows that the stapling becomes a fibrotic band. Gastorgastric anatomosis should be done in every instance, because gastroplastic proceures would compromise the completeness of obliteration of the transmural communicant veins.

P0153 PANCREASTRANSPLANTATION WITH BLADDER DRAINAGE: $H_2CO_3^-$ AND $Na^+$ LOSS IN DOGS AND RATS

Schang, T.*, Sutherland, D.E.R., Najarian, J., Timmermann, W.*, Hamelmann, H.*
Univ. of Minnesota, Minneapolis, USA, *Dept. of General Surgery, Univ. Hospital, D-2300 Kiel

Bladder drainage of exocrine secretion is widely applied in pancreas transplantation (ptx) for advantages both technical and in rejection monitoring. The disadvantage of electrolyte and bicarbonate loss was studied in rat and dog models.

Dogs: Following recipient pancreatectomy allogeneic ptx was performed with whole duodenum-to-bladder anastomosis (A: n = 7) or pancreatic duct to bladder anastomosis (B: n = 8). Dogs were monitored in metabolic cages postoperatively. 10 untreated dogs served as control group C.

Rats: Syngeneic (Lew-Lew) ptx was performed in diabetic recipients. I) Whole duodenum-to-bladder anastomosis (n = 39), a) no electrolyte substitution (N = 19), b) NaCl-substitution (n = 11), c) $H_2CO_3^-$-substitution (n = 9). II) whole duodenum-to-bladder anastomosis with ligated pancreatic duct (n = 5). III) duct-to-bladder anastomosis (n = 15).

Results:

Dogs: A: All dogs died before rejection could occure with acidosis, dehydration and low serum $Na^+$ at day $\bar{x}$ = 6 p.op. B: All dogs survived until grafts were rejectd at day $\bar{x}$ = 10 (serum glucose $\geq$ 150 mg/dl).

Rats: Ia) Without substitution all rats died at day $\bar{x}$ = 3 p.op., b) with NaCl-substitution they died at day $\bar{x}$ = 14, c) with $H_2CO_3^-$-substitution they died at day $\bar{x}$ = 5. III) All rats with duct-to-bladder anastomosis survived > 100 days. Serum $Na^+$/mval/L at day 2 p.op.: Ia) 126$\pm$ 6,3, III) 136 $\pm$ 2 (p 0.01).

Conclusion: Pancreaticoduodenal transplantation with bladder drainage leads to lethal loss of $Na^+$ and $H_2CO_3^-$ in dogs and rats. Sodium loss by the duodenum is crucial. Appliing this technique the duodenal segment should be as short as possible.

### P0154 PERI-HEPATIC PACKING IN LIVER TRAUMA

JEJ Krige, PC Bornman, J Terblanche

Department of Surgery,
University of Cape Town, Groote Schuur
Hospital and MRC Liver Research Centre,
Cape Town, South Africa

The efficacy of therapeutic liver packing used for control of haemorrhage in 22 (7.5%) of 294 patients undergoing operation for liver trauma during a six-year period (1983-1988) was evaluated. Sixteen patients had blunt trauma, 5: gunshot wounds, 1: knife stab. Nineteen had right lobe injuries, 3: left lobe, 5 also had hepatic vein injuries. Thirteen patients had documented coagulation defects. Six patients (27.3%) died post-operatively (head injuries: 3, multiple organ failure: 2, liver sepsis: 1). Four patients had recurrent bleeding from the liver injury despite packs in situ, 3 of whom required further surgery and repacking and one patient had bleeding controlled by selective hepatic artery embolization. Packs were removed from 16 survivors at laparotomy at an average of 3.1 days after insertion. Five patients re-bled during pack extraction and were successfully repacked. Packing provided ultimate definitive control of bleeding in 18 patients (82%). Seven patients (32%) developed intra-abdominal sepsis following packing, one of whom died. Sepsis occurred in patients with associated visceral injury or prolonged packing (> 3 days). Major morbidity of which sepsis was the predominant factor occurred in 12 (75%) of the 16 survivors.

Despite the high sepsis rate, therapeutic liver packing and planned re-laparotomy provided life-saving control of continued hepatic haemorrhage, aggravated by coagulopathy, when other methods of haemostasis had failed.

### P0155 ACUTE HEMORRHAGE IN CHRONIC PANCREATITIS

Iacono C., Serio G., Modena S., Trivisone M., Zicari M.°,
Taddei G.°, Girardi C., Recchia A., Dagradi A.,
Departement of Surgery and Radiology°
University of Verona ,Verona - Italy.

The erosion of pancreatic and peri-pancreatic arteries in case of pancreatic inflammatory disease can be clinically characterized either by G.I. tract or intra-retroperitoneal hemorrage, its frequence ranging between 1,7% (Trapnel et al. 1971)and 7,5%(Eckauser et al. 1980). Chronic pancreatitis (C.P.) is more often associated with this complication , which is lethal in most eveniences in case of delayed treatment (Stabile et al.). Aim of this presentation is to refer the personal experience on hemorrhages as a complication of C.P..

Between 1970 and 1988 we had 13 cases of massive hemorrhages out of 548 patients operated for C.P. (2,3%)(200 of these had one or more pseudocysts), which required emergency treatment due to G.I. bleeding in 11 cases and intra-retroperitoneal hemorrhage in 2 cases. Gastroduodenoscopy, done in 9 cases, disclosed upper G.I. tract bleeding in all eveniences, and at the papilla of Vater in 5 cases. CT, done in 10 cases, and Sonography, done in 7 cases showed the presence of pancreatic inflammation and/or pseudocyst, being able to suspect an hemorrhagic complication only in 4 cases.Selective arteriography was done in 11 cases, showing in 10 of them either extravascular leak of the contrast material (4 cases) or a pseudoaneurism (6 cases). In three cases angiography was followed by embolization of the affected vessel: in one it was the final treatment, being followed by surgery in the two other cases. The bleeding had its origin from the splenic a.(X6), pancreatico-duodenal a.(X4), left gastric a. (X2) and middle colic a. (X1). The performed surgical procedures were distal pancreasectomy and pancreoduodenal resection respectively in 3 and in 1 cases, and internal drainage of the pseudocyst in the remeining 8 cases, following ligation of the bleeding vessel at its origin or - if not possible - on the wall of the pseudocyst.

Three patients died postoperatively due to hemorrhagic shock or sepsis; the other are in a fair condition, with no hemorrhagic recurrences, at a 3 months to 10 years follow-up. In order to decrease the operative mortality we personally advocate an early and correct diagnostic approach, based on gastroduodenoscopy, sonography, CT and angiography, as well as a therapeutich approach which foresees as choice modality hemostatis with pancreatic and cystic resection; as alternative modalities definite hemostasis is achieved at the origin of the bleeding vessel or by fixing the vessel on the pseudocyst wall, followed by internal drainage, or embolization of the affected vessel.

References :
Trapnel J.T.: Ann. R. Coll. Surg. Engl. 1971: 49,361.
Eckauser F.E.,Stanley J.C., Zelenock G.B. et al : Surgery 1980: 88, 135.
Stabile B.E., Wilson S.E., Debas H.T.: Arch. Surg. 1983: 118,45.

### PO156 RESECTION OF HEPATOCELLULAR CARCINOMA WITH MASSIVE BILIARY TRACT INVASION

M. Kracht, N. Rotman, D. Mathieu, D. Cherqui and P.-L. Fagniez, Depts. of Surgery and Radiology, Henri Mondor Hospital, Creteil, France

Two patients with massive obstruction of the biliary tract by direct invasion of the tract by a small hepatocellular carcinoma have been resected successfully.
Patient 1, a 64 year old woman, prsented with a 3 cm tumour situated between segment II and III ducts, and was admitted with a clinical history of cholangitis. Patient 2, a 60 year old man, presented with a four cm tumour in segments VIII and IV, and had marked anomalies of liver function tests.
Both patients were explored by ultrasound and CT-scan which demonstrated material in the biliary tract. There was no massive portal invasion at the time of surgery.
Patient 1 had a left lobectomy, and patient 2 an extended right hepatectomy.
In both cases, the biliary tract was cleared from tumour clots with a Fogarty balloon catheter, and then flushed extensively with a saline solution.
Resection margins were clear in both patients. The postoperative course was uneventful.
Preoperative elevated alphafoetoprotein levels fell to normal after surgery.
Patient 1 was found to have arecurrent tumour two years after the resection, whereas patient 2 is considered disease-free at 21 months follow-up.
These cases suggest that even massive invasion of the biliary tract by a hepatocellular carcinoma does not preclude resection with a good survival rate if care is taken to obtain a complete tumour clearance of the biliary tract.

### P0157 LYSOSOMAL ENZYMES AFTER BILIARY DRAINAGE FOR OBSTRUCTIVE JAUNDICE

Akira Nakano, Kensuke Ogura, Yasuhisa Mochizuki, Hitoshi Sekido, Shuji Tsuchiya

Second Department of Surgery, Yokohama City University, Yokohama, JAPAN.

It is known that activity of lysosomal enzyme in liver is increased after ligation of bile duct in the rat, although it has not yet been determined whether the increased activity reflect augmented production or diminshed clearance of the enzymes. Recently we reported the presence of lysosomal enzymes in bile and lysosomes could excrete their content by exocytosis directly into bile. Our AIM was to test the hypothesis that the drainage of lysosomal enzyme into bile is a major mechanism for clearance of the hepatic lysosomal enzyme in obstructive jaudice. METHOD: Rats underwent operation with ligation and division of the bile duct. After seven days, permanent bile fistulae were constructed so as to permit cotinuous bile collection from freely-moving, unanesthetized rats. We collected bile for 72 hours and analyzed it for lipids and activity of lysosomal enzymes (ß-glucuronidase, N-acetyl-ß-glucosamidase). Rats were sacrified and analyzed livers for lysosomal enzymes activity before and after doing the biliary drainage. RESULTS: Activity of lysosomal enzyme in liver was increased rapidy after ligation of bile duct. After making the bile fistulae, amount of biliary lysosomal enzyme was increased gradually with the independent pattern of other biliary lipid. Corresponding changes in hepatic amount of lysosomal enzymes were seen; hepatic amount of lysosomal enzymes was decreased after doing biliary drainage of obstructive jaudice. CONCLUSIONS: Increased hepatic lysosomal enzymes in obstructive jaudice excreted mainly into bile in vivo, therefore biliary drainage can be advantage for patients with obstructive jaudice.

### P0158 RESULTS OF LIVER RESECTIONS IN LOCALIZED INTRAHEPATIC CHOLELITHIASIS

D FRANCO, F KEMENY, A KARAA, F KAHWAJI, C SMADJA, D GRANGE. Groupe de Recherche sur la Chirurgie du Foie et de l'Hypertension Portale. Hôpitaux Louise Michel, Evry ; Paul Brousse, Villejuif ; et Bicêtre, Le Kremlin Bicêtre, France.

Between 1983 and 1989, 13 patients (10 women and 3 men aged 20 to 67 years) with localized intrahepatic cholelithiasis were treated by surgical resections : one patient with recurrent stones, another with stenosis of a bile duct repair and 11 patients with primary stones. In the latter cases stones developed in cystic dilation of the intrahepatic ducts. Eleven patients had had one or more previous biliary surgical and/or endoscopic procedures. Severe cholangitis was the usual chief complaint and was triggered in 9 patients by previous biliojejunal anastomosis or endoscopic sphincterotomy. There were 5 right hepatectomies, 2 left hepatectomies and 6 left lateral segmentectomies. All patients had an infected intrahepatic bile. Postoperatively one patient developed a right subphrenic hematoma treated by reoperation and three patients had a subphrenic abcess resulting in percutaneous drainage. The rate of postoperative infection was much higher than in our experience of liver resection for tumors, probably resulting from peroperative inoculation of the peritoneal cavity by the infected bile. All patients were followed up for 1 to 6 years. There was no recurrent biliary stones in any of them. Two patients had recurrent cholangitis resulting from stenosis of previous endoscopic sphincterotomy in one and from stenosis of previous biliojejunal anastomosis in the second. Symptoms disappeared after reoperation. These results suggest that liver resection is the best treatment of localized intrahepatic cholelithiasis. Unappropriate biliary procedures should be avoided in these patients since they may result in severe cholangitis and in late biliary complications.

**PO159** SURGICAL TREATMENT OF HEPATOLITHIASIS WITH BILIARY STRICTURE

J.H. Chiu, W.Y. Lui, T.J. Liu, F.K. P'eng
Veterans General Hospital, Taipei,
Taiwan, Republic of China

In contrast to ordinary cholesterol cholelithiasis, hepatolithiasis pose serious health problems in Eastern Asia (Nakayama and Koga 1984). Although much effort has been devoted to the surgical treatment of hepatolithiasis (Choi, Wong and Ong 1982; Sheen and Ker 1984), the prognosis of this disease is far from satisfactory. Recent work has been led to the correlation of treatment and the pathological findings (Koga, Miyazaki, Ichimiya, and Nakayam 1984).

From Jan. 1980 to Dec. 1988, 657 patients with intrahepatic gallstones in Taipei and Taichung Veterans General Hospitals were reviewed. Among them, 156 patients proved to have intrahepatic biliary stricture were divided into five groups, according to the surgical modality (Table 1). The overall surgical mortality was 7.5% and morbidity was 18.5% (Table 2). Thebetter outcome were obtained by partial hepatectomy in group IV. The mortality was 8.1% and morbidity 16.2% . About 93.5% patients had good or fair subjective improvement (Table 3) and only 6.1% patients received re-operation for the stones recurrence (Table 4). We conclude intrahepatic biliary stricture with distal ductal stones formation is best treated with partial hepatectomy. However, the preoperative liver functions should be carefully evaluated.

References:
Nakayama F, Koga A. World J Surg 1984: 8: 9.
Choi TK, Wong J, Ong GB. Br J Surg 1982: 69: 86.
Sheep PC, Ker CG. Prog in Clin & Biol Res 1984: 152: 303.
Koga A, Miyazaki K, Ichimiya H, Nakayama F. World J Surg 1984: 8: 36.

**PO160** A NEW METHOD FOR HEPATIC INTRA-ARTERIAL HIGH DOSE CHEMOTHERAPY USING DIRECT HEMOPERFUSION UNDER HEPATIC VENOUS ISOLATION

Y. Ku, M. Saitoh, S. Fujiwara, T. Iwasaki, M. Tominaga, Y. Maekawa, H. Ohyanagi, Y. Saitoh
1st Department of Surgery, Kobe University School of Medicine, Kobe, Japan

We undertook hepatic artery infusion of high dose adriamycin (ADR) in three patients with unresectable hepatocellular carcinoma utilizing direct hemoperfusion (DHP) under hepatic venous isolation (HVI). Five minutes infusion of ADR at dosages of 100-150 mg/m$^2$ was combined with the DHP of 20 minutes under HVI. During treatment, hepatic effluent was drained and adsorbed selectively by the DHP through the tip of a cuff-cannula (18-24F), which was placed at the retrohepatic inferior vena cava (IVC) via the left femoral vein. HVI was obtained by concomitant occlusions of the retrohepatic and suprahepatic IVC using the cuff inflations. The remaining IVC blood was drained by a cannula (16-18F) through the right femoral vein and joined with the hepatic effluent before the centrifugal pump. A return cannula (18F) was placed in the left axillary vein. Peak systemic levels of ADR were observed between 5 and 10 minutes after the initiations of drug infusions, the mean value of three patients being 1.19 µg/ml. Peak values at the inlet of DHP were 19.71, 25.59 and 17.16 µg/ml in patients 1, 2 and 3, respectively. A peak was obtained 5 minutes at the outlet, the values being 1.75, 1.82 and 0.79 µg/ml. Postoperative CT studies revealed remarkable reductions in tumor sizes of all patients. Serum transaminases reached a peak on day 2 and rapidly decreased within a week. Postoperative recoveries were uneventful. Serious side effects of ADR were not encountered.

In conclusion, the use of this method may allow a significant breakthrough for the treatment of unresectable hepatic tumors.

### P0161 A NEW PORTAL VENOUS CANNULA FOR ORTHOTOPIC LIVER TRANSPLANTATION

KC Tan,JB Wood,J Bokos,S Chitmitrapap

Kings College Hospital, London, UK

One of the most important technical advancements in orthotopic liver transplantation has been the development of a safe bypass system during the anhepatic phase (Shaw et al 1984). Without this, there is often a resultant reduction of cardiac output and the sudden elevation of portal pressure may culminate in severe bleeding. Most bypass systems involve cannulating the portal vein and the inferior vena cava via the femoral; the blood is then returned via the axillary vein.

It is difficult maintaining the tapered and smooth-walled shunt within the short portal vein. Often the shunt is inserted too far into one of the tributaries resulting in inadequate portal decompression. It is also difficult to position the straight shunt away from the operative field without intermittent kinking during implantation.

We described a new heparin bonded portal venous cannula for use during orthotopic liver transplantation. We have used it in our last 18 adult patients achieving a consistent portal flow of more than 2 litres per minute. This new shunt has a 'shoulder' 4 cm from the caged tip which allows it to be securely maintained within the portal vein. With its 60 degree curvature 7 cm from the tip the shunt can be conveniently positioned away from the operative field. In addition, this cannula will allow the patient to be bypassed before the liver is fully mobilised. This is most useful in the patient with portal hypertension who has had previous upper abdominal surgery as prior portal decompression may reduce the anticipated blood loss during hepatectomy.

Reference

Shaw B W Jr et al. Annals of Surgery 200(4): 524-534.

**P0162** RAT MODEL FOR CELL KINETIC MEASUREMENTS OF THE EFFECT OF HEPATECTOMY ON LIVER TUMOR.

PVM Pahlplatz, A. Marinelli,CJH van de Velde,
University Hospital, Leiden, The Netherlands

Partial hepatectomy has a positive influence on the development of artificially induced liver metastasis in the laboratory animal (Fisher 1959) Several studies have also shown that removal of the primary tumor has a stimulating effect on the growth of residual tumor, possibly due to increased levels of 'growth stimulating factors' (Gunduz 1979).
Out of concern that these cytokinetic effects might have clinical significance after resection of solitary livermetastasis, we developed a model to perform cell kinetic measurements.
In the albino WAG/RIJ rat livermetastasis were induced by injecting $1x10^6$ CC531 colonca cells in the ileocolic vein. When no partial hepatectomy was performed 0- 10 metastasis were seen after 28 days, while in partly hepatectomized rats 20 - 40 metastasis were seen. Similar growth acceleration was seen when the tumorcells were subcapsular inoculated.
Earlier studies from our laboratory showed thas by in vivo labelling of tumor cells with 5'-bromode oxyuridine (BrdUrd) reliable data about cell kinetic changes after surgery
are acquired (Van Dierendonck). Labelling subcapsular induced metastasis with BrdUrd showed a labelling index of 40% positive cells after 1 hour and 85% after 72 hrs continuous labelling.
Conclusion: By in vivo labelling with BrdUrd it will be possible to quantify the effect of partial hepatectomy of the cell kinetics of residual tumor in the liver.

References:
Fisher B. Cancer 1959; 12:929-31
Gunduz N. Cancer Res 1979; 39: 3861-65
Van Dierendonck JH. 1990, Univ. Thesis Leiden

### P0163 LIVER HYDATIC CYSTS OF HILAR AND POSTERIOR LOCATIONS. TREATMENT AND DIAGNOSTIC OPTIONS

J.M.SCHIAPPA, J.GIRÃO

Hosp.dos Capuchos - Serviço 6 - Lisbon,

PORTUGAL

The hydatic cysts presenting with a posterior and with a central (hilar) location, present specific problems concerning diagnosis and, particularly, treatment.

Considering this, the authors review their series of 55 patients with liver hydatic cyst, in which 21 (38%) have these locations, and discuss and present their actual options when facing these situations or its complications.

The main issues are about how to determine and how to prevent posible complications, as well as when to resect.

**PO164** RAPID-BOLUS DYNAMIC CT IN ACUTE PANCREATITIS WHAT IS THE SIGNIFICANCE OF CT NECROSIS?

N.J.M. London, T. Leese, J.M. Lavelle,
Department of Surgery, Leicester Royal
Infirmary, Leicester, UK

Thirty-four patients with prognostically severe acute pancreatitis have undergone rapid-bolus dynamic CT scanning. Twenty-four scans showed 'CT necrosis' which in 13 patients involved $<$1/3 of the pancreas, in 4 patients 1/3-2/3, 5 patients $>$2/3. Cytological examination of fine-needle aspirates from 'CT necrotic' areas revealed necrotic amorphous debris. Repeat scans at 6 weeks in 3 patients with $>$2/3 necrosis showed persisting non-enhancement and ERP in one of these patients failed to demonstrate any pancreatic ducts in the 'CT necrotic' area. The 10 patients whose scans did not show 'CT necrosis' had clinically mild attacks. Amongst the 24 patients whose scans showed necrosis, 10 proved to have mild attacks; 14 had severe attacks. There were 6 deaths, 4 from multi-organ failure and 2 from pancreatic infection. The 2 patients who died from pancreatic sepsis were the only 2 with infected fine-needle aspirates. Disease severity was unrelated to the extent or site of 'CT necrosis'. Three patients developed pseudocysts in areas of 'CT necrosis'. We conclude that 'CT necrosis' does represent pancreatic necrosis but that disease severity is unrelated to its site or extent. Infected necrosis carries a high risk of death whilst sterile necrosis may lead to pseudocyst formation and in a proportion of patients (20%) is associated with death from multi-organ failure.

### P0165 PRIMARY HEPATIC TUMOURS OF MESENCHYMAL ORIGIN

L. Angrisani, T Ismail, S Hubscher,
R West, J Buckels, P Mcmaster
The Liver Unit, Queen Elizabeth Hospital,
Birmingham, U.K.

With the increasing use of imaging techniques, there are increasing reports of primary hepatic tumours of mesencymal origin. We reviewed our experience of 19 patients (16 female; mean age 37 years, range 3-61 years) over a 5 year period to August 1989.

Liver resection or transplantation was performed in 11 patients (60%) with 1 perioperative death. Six patients had a benign giant cavernous haemangioma. Three underwent hemi-hepatectomy and the remaining 3 were managed conservatively with serial CT Scan monitoring. All were well at a median follow up of 36 months (range 12-84 months). Three patients had epitheloid-hemangio-endotheliomas. One had lung metastases and underwent chemoradio-therapy; 2 patients had liver transplantation and are alive and disease free at 6 and 57 months. Angiosarcoma was diagnosed in 5 patients all of whom died within 12 months of diagnosis. Three had chemotherapy and 2 had liver replacement but both developed recurrence despite a complete resection. Two patients had a partial hepatectomy for a leimysocarcoma. One died post-operatively (had cirrhosis) and the other is alive at 18 months. A further 3 patients (all female) had a juvenile type embryonal sarcoma. Two underwent partial resection whilst the third patient with lung metastases is responding to chemotherapy.

Our experience confirms that primary hepatic tumours of mesenchymal origin are a separate subgroup with a range of biological activity with require an integrated approach to their management at a specialized unit.

P0166 A SURVEY OF GALLBLADDER OPERATIONS.

M. G. Neely, University of Queensland,
Mater Misericordiae Hospital, Brisbane,
Australia.

A study of 811 consecutive patients in a surgical unit undergoing operations on their gallbladders over the past 15 years is presented. The ages ranged from 14 to 90 years with a mean of 48 years. There were 79% females and 21% males. Forty (5%) of these patients had had some form of cancer previously diagnosed, the commonest being colonic. Thirteen patients had a concomitant peptic ulcer and 80 an hiatus hernia. Twenty nine patients had associated acute pancreatitis. Six percent were jaundiced at operation. Five patients were pregnant at the time of operation. While some were operated on during their first attack, the mean duration of symptoms was over two years. Under half of the patients had oral cholecystograms. Of these over 60% showed stones while over 30% showed a non functioning gallbladder. Three percent showed cholecystitis glandularis proliferans. About half the patients had ultrasonography of their gallbladders. Over 90% of these showed stones. There were 807 cholecystectomies and 8 cholecystostomies. Operative cholangiograms were done in 84% of cases and were considered normal in 75% of these. Stones were shown in 11% of these. The common bile duct was shown to enter the duodenum at the middle of the second part of the duodenum in only 71% of investigations. In four cases the bile ducts were injured. At operation fistulae were found to either the duodenum or colon in nine cases. Mucoceles were present in 5% of cases and empyemas in 4%. The gallbladder was gangrenous in 2% of cases and perforated in 4 cases. No stones were found just under 3% of cases. Cholecystitis glandularis proliferans was found in 3.5% of cases. Carcinoma of the gallbladder was present in six cases, their mean survival time being 241 days. The common bile duct was explored in 89 cases and no stones were found in 19 of these. Retained stones were present in 6 cases requiring subsequent removal. Four patients developed pancreatitis post-operatively. The wound infection rate was 4%. There were two deaths within 30 days, one of which occurred in a patient with gallstone pancreatitis and the other in a gallbladder carcinoma.

### P0167 THE ROLE OF HEPATIC RESECTION IN THE MANAGEMENT OF BLUNT LIVER TRAUMA

M. Hollands, T. Wilson, J. M Little.
Westmead Hospital, Sydney, Australia.

The place of liver resection in the management of blunt liver injuries is controversial. The aim of this study was to consider the indications for resection and review its place in light of the associated morbidity and mortality.

Data on 306 patients with liver injuries was collected prospectively over a 10 year period to January, 1989.

42 patients (14%) required resection. There were 23 grade VII and 18 grade VI injuries. Resectional debridement utilising the plane of injury was used in 35 patients whilst resection along anatomical planes was performed on 7 occasions. In 32 patients bleeding was the major problem which in 23 originated from the hepatic veins. In 7 patients the major problem was devitalised liver parenchyma and in 3 patients an intra-hepatic bile duct injury. 15 patients died (36%). The most common cause of death was continued bleeding (9/15), usually from chest and retro-peritoneum as well as liver (7/9). Survivors spent a median of 32 days in hospital (range 11-162) and sustained a median of 2 complications (range 0-6).

In conclusion the principal indication for resection is uncontrolled bleeding, especially from the hepatic veins. The morbidity and mortality reflect the degree of liver injury and other associated injuries rather than the operation performed.

P0168 FACTORS INFLUENCING SUCCESSFUL EXTRACORPOREAL GALLSTONE LITHOTRIPSY (ESWL)

R Reinhold, J Rothschild, R Malt
NE Medical Center, Mass General Hospital
Boston, USA

Factors influencing successful ESWL was analyzed in 82 consecutive patients treated at NEMC/MGH and enrolled in the U.S. Dornier ESWL trial. Only pts with 1-3 radiolucent stones with cumulative diameter $<$30 mm. were included in this analysis. All patients were prospectively randomized to URSO vs placebo. Data was analyzed for fragmentation (FR) at six weeks and stone free incidence (SF) at six months. FR was considered successful if largest FR$\leq$5 mm by ultrasound. Patients were stratified into 2 groups: Grp I: pts achieving FR after a single ESWL vs Grp II: pts requiring second ESWL (largest FR$>$5 mm).

| | Grp I Pts. | | Grp II Pts. | |
|---|---|---|---|---|
| N= | 49 | | 33 | |
| Female | 32 | (65%) | 20 | (61%) |
| Receiving URSO | 26 | (53%) | 16 | (48%) |
| Single Stone | 40 | (82%) | 23 | (70%) |
| Mean FR size (mm) | 3.1 | | 10.8 | |
| Stone free @ 6 months | | | | |
| URSO | 55% | | 18% | |
| PLACEBO | 13% | | 25% | |

64% of Grp II achieved successful FR after ESWL #2 yet $<$20% were SF despite URSO. URSO administration was identical in both grps i.e. URSO had no correlation with successful initial FR. Only patients with successful initial FR + URSO achieved satisfactory SF at six months: the other 3 groups were $<$25% SF. This study suggests that patients with optimal gallstones receive one ESWL prior to the initiation of URSO. If FR is successful, pt should begin URSO for at least 6 months. If ESWL #1 technique was adequate, the cost effectiveness of ESWL #2 and/or initiating URSO should be questioned.

### P0169 CARCINOMA OF THE GALLBLADDER

D.J. Gouma, M.F. von Meyenfeldt
University Hospital Maastricht, The Netherlands

Carcinoma of the gallbladder is generally associated with a poor prognosis, because most patients present with advanced disease. The presenting signs and symptoms however are reported to be of little value for the prognosis.
The aim of this study was to evaluate retrospectively the clinical course of patients with a gallbladder carcinoma and especially the relation of symptoms and outcome.
From 1977 until 1988 72 patients (58 female; 14 male) were admitted with a histologically proven gallbladder carcinoma. Fourty four patients (61%) had also gallstones. The mean age was 73 years (range 46-90). Fifty eight patients complained of pain, nausea and vomiting 41, jaundice 29 and weightloss 37. A palpable mass was found in 38 patients.
Treatment: Cholecystectomy was performed in 16 patients; cholecystectomy + exploration of the common bile duct in 8 patients; PTC (13) with biliary drainage in 7 patients and biliary bypass in 7 patients; laparotomy + biopsy in 14 patients. No treatment was performed in 20 patients. Staging according to AJCC was stage 0: 2 patients; stage 1: 4 patients; stage 2: 5 patients; stage 3: 10 patients; stage 4: 47 patients; unknown: 4 patients.
The mean survival was 266 days. Five years survival was only found in stage 0 and 1. The mean survival according to signs and symptoms is summarized in table 1.

<u>Table 1</u>

| Symptoms | Yes | % | Surv.(days) | No | % | Surv.(days) |
|---|---|---|---|---|---|---|
| Pain | 58 | 81 | 312 | 13 | 19 | 30 |
| Nausea/vomiting | 41 | 57 | 389 | 30 | 43 | 105 |
| Weight loss | 37 | 51 | 181 | 34 | 49 | 380 |
| Jaundice | 29 | 40 | 118 | 43 | 60 | 372 |
| Mass palp. | 38 | 53 | 132 | 34 | 47 | 419 |
| Temperature | 10 | 14 | 590 | 62 | 84 | 230 |

Conclusions: The overall survival confirmed the advanced stage of the disease at presentation and the poor prognosis. Signs and symptoms were associated with the outcome probably reflecting factors related to symptomatic gallstone disease.

P0170 ORIGIN OF LACTATE DEHYDROGENASE ELEVATION IN ACUTE PANCREATITIS

C Wilson, W D Fraser, M D Gardner, C W Imrie.
Departments of Surgery and Biochemistry,
Royal Infirmary, Glasgow, UK.

Lactate dehydrogenase (LD) elevation is an important marker denoting severe acute pancreatitis and which may indicate pancreatic necrosis. Its pattern of elevation differs in attacks secondary to alcohol and gallstones suggesting a different mechanism or origin. Of 100 patients having LD assayed, its origin was investigated by studying isoenzyme patterns in 20 patients with LD elevation.

Eight showed a "skeletal muscle pattern" all in association with marked creatine kinase (CK) elevation (mean 1856U/1, range 760-6400U/1). Aetiologies of the attacks included alcohol (3), gallstones (3), hypothermia (1) and post-ERCP with renal failure (1). Multiple intramuscular injections were a common prelude and the likely explanation for the LD elevation in 6. Nine patients showed a "liver pattern"; 7 had gallstones, 6 showing a "liver-type" transaminase elevation (ALT $>$ AST). Two with an alcohol aetiology had normal transaminases. The CK elevation was less marked in this group (mean 441U/1, range 75-950U/1) and was associated with fewer intramuscular injections. An LD pattern suggesting a pancreatic origin was seen in 2 patients and a "shock-type" pattern in one. Our findings suggest that LD elevation in acute pancreatitis results from several different mechanisms and is not specific for pancreatic damage nor useful in identifying the aetiology of such damage.

### P0171 HISTOGENESIS OF GALLBLADDER CANCER

A. Inada, T. Tatsumura, M. Tsuda,
K. Yamamoto and F. Konishi*
Toyama Medical and Pharmaceutical
University, Toyama, Japan
* Kanazawa Medical University,
Ishikawa, Japan

In order to study the histogenesis of gallbladder cancer, metaplastic changes and dysplasia in the mucosal epithelium were investigated in 36 cases of gallbladder cancer.

Intestinal metaplasia and dysplasia was observed at the rates of 81% and 61% in non-cancerous tissues, and goblet cells or endocrine cells were found at the rates of 75% in cancerous tissues.

In addition, CPS III type of mucin (by concanavalin A-hoarseradish peroxidase method) (Katsuyama and Spicer 1978), which is preferably demonstrated in the pyloric glands, was observed in the tumor cells of 47% of cancers. Thus, gastric metaplasia as well as intestinal metaplasia seems to be important as a predisposing lesion to gallbladder cancer.

By means of reconstruction method carried out on the specimens of cancer it was shown that multifocal gradual transition among metaplasia, dysplasia and cancer, suggesting that dysplasia is an important step in cancer development.

As for mucin secretion, the rate of sialomucin-containing cells was notably high in the lesions of dysplasia and cancer, increasing in intensity in this order, accampanied with positive CEA.

The results of the present study support the hypothesis that cancer arises from metaplasia-dysplasia-carcinoma sequence.

Recently, percutaneous transhepatic cholecystoscopic examination has become commonly use nowadays. If the finding obtained by this technique reveal granular appearance in the mucosa, it frequently consists of dysplastic change or in some cases microscopic cancer. Therefore cholecystectomy should be perform in such a case.

Reference:
Katsuyama T, Spicer SS. J Histochem cytochem 1978; 26: 233

### PO172 DRAIN OR ULTRASONOGRAPHICAL MONITORING AFTER CHOLECYSTECTOMY

A. Bilge, A.Sağlam, M. Güleç, K. Ödev

Erciyes University, Medical School, Kayseri/Turkey

Drainage is a common practice after cholecystectomy. Some authors claim that routine drainage of the subhepatic region following cholecystectomy increases the morbidity. This study was designed to investigate whether using a drain after cholecystectomy is necessary.

Eightytwo patients undergoing cholecystectomy (68 F, 14 M) were studied. In the first group (n=57) drains were not used. In the second group (n= 25) the subhepatic region was drained. Three days later post-operative ultrasonographycal examination of all patients showed one patient in the non-drainege group had fluid accumulation. This dispersed after three weeks. Another three patients in this group developed wound infection and abscess. No fluid accumulation was detected in the drainage group but changes of wound dressings were at least once a day. There were no post-operative complications for this group except for a minor wound infection in one patient.

This study indicates that intra-abdominal fluid accumulation very rarely occurs after cholecystectomy. Since only one fluid accumulation was detected in the non-drainage group, fluid was probably absorbed by omentum. Drainage did appear to increase the fluid flow and it was necessary to change the wound dressing once a day for three days. The drain itself may cause more fluid oozing.

It is concluded that, generally, there is no difference between using and not using a drain, one expect that a drain appears to increase the morbidity. Post-operative ultrasonographical examination is better than that using a drain after cholecystectomy.

P0173 *ODDI SPHINCTER RESISTANCE AFTER CHOLECYSTECTOMY*

*S.Akgün,A.Menteş*

*Dept. of Surg.,Agean University,İzmir,Turkey*

*Oddi sphincter resistance has been determined before and after cholecystectomy in eight patients,who were healthy except for gallstones.A luminal catheter was introduced into the duodenal lumen through the cystic duct and was connected to a recording system.Recordings were made as the catheter was pulled in a stationed fashion.A high pressure zone was detected when the catheter was pulled into the Oddi sphincter complex,in every case. Following cholecystectomy with complete clearence of the cystic to choledochal junction,including nerve fibers at this site,the same procedure was repeated.Resistance showed no significant increase from duodenal baseline in any patient.These results were controlled manometrically in 10 separate patients.The mean intracholedochal pressure was 15 cm$H_2O$ before and 11 cm $H_2O$ after cholecystectomy and exhibited a highly significant (t=10.29,p=0.00) decrease. We suggest that,these results may either be due to cholecystectomy per se, or to a physiological sphincterotomy effect caused by transection of pericystic nerves,during cholecystectomy.*

### P0174 SURGICAL DIAGNOSIS IN A DEVELOPING COUNTRY.

G.Grunblatt; M.León; G.Lamura; J.Miguez.

Hospital M.Perez Carreño, Cirugía III

Caracas, Venezuela.

Since 1983 our country suffers from a severe economic recession marked with currency devaluation and high inflation rates. This unfortunate situation has caused a severe shortage of medical, surgical, and laboratory supplies, which are mostly imported. It is for these reasons that in our hospital, the largest medical facility in the nation, many a times our surgical patients have to undergo procedures without the pre operative testing common in developed countries.

Over a period of five years, 1984-1988, we selected two groups of emergency patients according to the diagnostic facilities available at the time their given surgical procedure was performed. Both groups included multiple trauma, gunshot wounds, and knife stabbed patients. They were included in this series if upon being laparatomized the intra operative diagnosis involved damage to one or more of the following organs: liver, gall bladder and biliary tract, and pancreas. Group (I) involves 150 patients, and group (II), 250.

Group (I) : clinical diagnosis + ultrasound + laboratory + radiology pre and intra operatively.
Group (II): clinical diagnosis + basic laboratory.

Findings were as follows:

| | Group (!) | (II) |
|---|---|---|
| mean operative time | 3hrs.10min. | 3hrs.45min. |
| intra operative complications | 8% | 7% |
| intra operative mortality | 3% | 4% |
| medical complications post op. | 4% | 5% |
| post operative mortality | 2.5% | 5.5% |
| patients in ICU | 3.2% | 3% |
| mean hospital stay | 12days | 11days |
| mean age | 27yrs.6m. | 31yrs.4m. |
| male/female | 135/15 | 221/29 |

### P0175 A CASE REPORT OF HEPATOCELLULAR CARCINOMA ASSOCIATED WITH HEPATOLITHIASIS

A. Oguro, H. Taniguchi, A. Itoh, T. Daidoh, N. Tsukuda, Y. Shioaki, T. Yamane, K. Sawai, T. Takahashi
First Department of Surgery, Kyoto Prefectural University of Medicine, Kyoto, Japan

Though cases of cholangiocarcinoma associated with hepatolithiasis are considered to result from repitition of chronic intrahepatic cholangitis and stagnation, the relationship of hepatocellular carcinoma and hepatolithiasis has not been clarified and there has been no report of it at all. As we experienced a case of hepatocellular carcinoma associtated with hepatolithiasis, the case will be reported as a first one in the world.

The patient was a 44-year-old male who underwent liver resection for hepatocellular carcinoma associated with hepatolithasis. He had had cholecystectomy for cholecystolitiasis at the age of 18 and choledocholithotomy for choledocholithiasis at the age of 28, and it was unknown whether then he had intrahepatic stones. The tumor was located at the posterior superior segment of the liver with a daughter nodule. Intrahepatic bile ducts of the right lobe were markedly dilated and filled with numerous pigment stones. Right lobectomy of the liver was performed. The tumor was diagnosed as type 2 hepatocellular carcinoma according to Edmondson's classification, and there was no evidence of cholangiocarcinoma in histological examination.

It was suggested that hepatocellular carcinoma was can occur in the liver with intrahepatic bile stasis and chronic cholangitis due to hepatolithiasis as well as cholangiocarcinoma.

References:
Edmondson HA, Steiner PE. Cancer 1954:7:462

### P0176 SQUAMOUS CELL CARCINOMA OF THE COMMON BILE DUCT – A CASE REPORT –

K. Nakagawa [1], N. Takakura [1], T. Sakata[1]

H. Mimura[2] and K. Orita[2]

[1] Shyobara Red Cross Hospital, Hiroshima,
[2] Okayama University Medical School, Department of Surgery, Okayama, Japan

Squamous cell carcinomas of the common bile duct have been reported infrequently since Cabot's first report in 1930.

A 68-year-old male with jaundice who proved to have tumor of the common bile duct underwent pancreatoduodenectomy combined with a resection of the hepatoduodenal ligament including the bile duct, portal vein and hepatic artery. Portal vein was anastomosed end to end, and the gastroduodenal artery was anastomosed to the right hepatic artery. Histologically, the tumor was a well-differentiated squamous cell cercinoma of the intrapancreatic bile duct, infiltrated to the pancreas slightly. He passed 15 months with no evidence of recurrence after operation.

Concerning the etiology of this tumor, it is theorized that the normal columnar epithelium of the bile duct under the chronic inflammatory stimulus can change into squamous epithelium leading to squamous metaplasia and eventually carcinoma. On the other hand, it is also considered that squamous cell carcinomas in this unusual sites are more likely to be due to squamous metaplasia of the adenocarcinoma itself than to malignant transformation of a previously benign metaplastic epithelium.

### P0177 JEJUNOSTOMY IN HPB SURGERY

E. Stanowski, B. Owczarczyk, J. Lapiński
Postgraduate Medical Center, Warsaw,
Poland

In this paper we report the results of applying jejunostomy in patients who underwent operations of upper alimentary tract as well as in operations of pancreas and biliary tract. In our Clinic jejunostomy has been performed in 62 patients in the period between 1986-1989: 22 women and 40 men in the age group between 20-80 years. Altogether 17 patients ie 27.4 per cent underwent HPB surgery:

| | |
|---|---|
| pancreatic carcinoma | 8 |
| Vater's papilla carcinoma | 2 |
| complications after biliary operations | 5 |
| after pancreatoenterostomy leakage | 2 |

The jejunostomy was performed by the technique introduced by H Delany et al. During the first two days after operation the jejunostomy was used for relief and during the next days for feeding. The catheters have been removed at varying times, postinsertion, from five days to six months.

The volume of secretion was estimated after 1-2 days. The level of albumin and hemoglobin was monitored before and after operation. Among patients with complications after operation of biliary ducts and pancreatoenterostomy fistulas the wounds healed by first intention. The leakages healed as well.

In case of biliary fistulas by Kehu's drain the jejunostomy was used for administration of bile. In patients with cancers the feeding jejunostomy was performed until their deaths.

Slight complication was observed among 4 patients (out of 17): 2 patients due to the poor food toleration, 1 patient due to a slight leakage, 1 patient due to his accidentally removing his catheter. Our experience shows that the jejunostomy is a simple, safe surgical procedure for feeding patients after HPB surgery, which can be used not only in a hospital but also at home.

References:
Delany H, Carnevale N, Garrey J, Surgery 1973:73:786

### P0178 ALTERATIONS IN LIVER FUNCTION AFTER ELECTIVE HEPATIC RESECTION

D.Humzah, J.Pain, E.Howard
King's College Hospital, London, UK

Close attention to postoperative care is essential for minimising mortality and morbidity after hepatic resection. The perioperative liver function tests of 35 patients undergoing major elective hepatic resection have been studied. No patients had underlying cirrhosis or major postoperative complications. Patients were subdivided into three groups: Group A (n=11), adults undergoing <50% liver resection; Group B (n=12), adults undergoing >50% resection; Group C (n=12), children.

Serial measurements of serum bilirubin, alkaline phosphatase, aspartate amino transaminase, gamma glutamyl transferase, albumin and total protein were taken preoperatively and on seven occasions over the first 21 postoperative days. For each parameter measured a similar pattern of change occurred in all groups with the exception of bilirubin which was higher ($p<0.05$) in Group B immediately after surgery compared with Group A. Bilirubin had fallen to normal levels in all groups by 14 days. Marked initial rises in transaminase fell to normal levels within seven days. There were no significant changes in alkaline phosphatase or gamma glutamyl transferase after surgery. Albumin and total protein fell after surgery but reached normal levels within 10 days.

Following uncomplicated hepatic resection changes in liver function follow typical patterns. Except for changes in bilirubin level these are unaffected by the age of the patient or extent of resection. A recognition of the changes that normally occur will help in the management of these patients.

### P0179 EXPERIENCE WITH SURGICAL TREATMENT OF HYDATID DISEASE OF THE LIVER

Xu Ming Qian
Research Unit of Hydatidology of
The People's Hospital of Xinjiang, Urumqi
People's Republic of China

There were 1390 patients with hydatid disease treated by operations between 1953-1988. Of these, 1002 cases had hydatid cyst in the liver and 1113 operations revealed 1485 echinococcus cysts.

The method of removal of the hydatid cysts: 1. Hepatic lobectomy 2. Removal of intact hydatid cyst. 3. Removal of hydatid cyst by puncturing.

Management of residual cavity: Removing the cyst left a cavity in the liver and bile containing bloody effusion accumulated which forming retention cyst usually resulted in liver abscess.

The clinical experiment studies of this series revealed the mechanism of cavity complicated with biliary fistula and natural rule of the cavity closure, allowing rational ectocyst cavity management and modification of operative method and reducing the frequency of postoperative complications as well as shorten the healing period.

In our series of 1002 cases, 6 deaths occurred (0.54%).

**P0180** **RENAL CHANGES IN CDCA IN DOGS CAN IT BE A NEW PERSPECTIVE IN ACUTE RENAL FAILURE IN JAUNDICE?**

*Mehmet TUĞCU,Rasih YILMAZ,Gül YÜCE, Orhan ÖZBAL*

*General Surgery and Pathology Dept's , Medical School,Ege U.Izmir/TURKEY*

*Acute renal failure is one of the most common and serious complication of jaundice due to extrahepatic biliary tract obstruction(1,2) Hepatic paranchima injury and depressive effect of hyperbilirubinemia are responsible from functional and histological destruction of renal system.*

*CDCA prevents hepatic changes due to cholestasis in hepatocytes, Thus,the depressive effect of liver injury does not seen upon other systems in organism.Isolated "cholemia" model is created with CDCA in many experimental studies.The renal changes are detected in this model.*

*CDCA(Choledochocaval anastomosis) was performed in 14 Mongrei dog Dogs were observed for 10-11 days and then sacrified.Histopathologi - cal changes were searched in post-mortem period.*

*Acute tubuler necrosis were observed in 11 of 14(78%) dogs.There were swelling and peritubular edema in other 3 dogs.Focal necrosis areas were present macroscopically in 5 dogs.*

*The increasing BUN and creatinin levels were observed during follow-up period.Oliguria were seen in all dogs.Anuria were observed in 7 dogs between 5th-10th days.Creatinin clearence abolished in all dogs*

*These results are similar to clinical findings of diseases seen with hyperbilirubinemia.In the other side,there is not cholestatic changes in this model.Hyperbiliruinemia must be responsible from renal changes in patients without occurence hepatic dysfunction.*

*1.Allison MEM :Br J Surg 66:392-397,1979*
*2.Alon U :Clin Sci 62:431-433,1982*

PO181

### THE EFFICACY OF SOMATOSTATIN IN CONTROLLING POST-INJECTION SCLEROTHERAPY BLEEDING FROM VARICES, OESOPHAGEAL ULCERS AND OESOPHAGITIS

S Jenkins, S Ellenbogen, N Jaser
J Baxter & R Shields
University of Liverpool, Liverpool, UK

Bleeding from the varices themselves, oesophageal ulcers or oesophagitis after injection sclerotherapy is usually minor but can occasionally be massive and difficult to control. Since somatostatin (SRIF) is a safe and effective treatment for the acute variceal bleed we have evaluated it's efficacy in these post-injection sclerotherapy complications. Thirty-eight patients experienced a significant upper gastrointestinal bleed at various times after injection sclerotherapy. A significant bleed was defined as either a systemic disturbance: blood pressure $<$100 mm Hg ; pulse$>$100 beats min requiring blood transfusion or the necessity to transfuse 2 or more units to restore the haemoglobin levels. Endoscopy revealed that 16 patients were bleeding from varices, 14 from oesophageal ulcers and 8 from oesophagitis. All patients were treated with SRIF (bolus dose of 250 µg followed by a continuous infusion of 250 µg/h) for between 2 and 7 days. Haemorrhage was successfully controlled in all patients bleeding from oesophagitis and in 12 of the 14 bleeding from oesophageal ulcers by somatostatin infusion. In the two patients with persistent bleeding from ulcers, haemorrhage was eventually controlled by hourly bolus injections of SRIF for a period of 24 h superimposed on the continuous infusion. SRIF was also successful in controlling haemorrhage in 15 or the 16 patients bleeding from their varices. The results of this study suggest that SRIF is a safe and effective treatment for controlling severe haemorrhage after injection sclerotherapy.

### P0182 PLACE OF ENDOBILIARY MANAGEMENT OF PATIENTS WITH OBSTRUCTIVE JAUNDICE

Karimov Sh.I.,Ahmedov R.M., Kim V.L.
Ergashev U.Yu.
Medical Institute, Tashkent, USSR

Radical interventions in patients with obstructive jaundice have a high post-operative lethality due to increasing hepatic insufficiency. In connection with this fact percutaneous transhepatic endobiliary interventions are applied more and more widely as the first stage of preparation for the main radical method or as a final treatment.

The results of management in 129 patients with obstructive jaundice from who 71 had benighn diseases of bile ducts and 58 patients had jaundice of malignant genesis are analysed from this standpoint. The number of aged and elderly patients was 64.5%. Ninety per cent of patients had severe concominant cardiovascular and respiratory diseases.

Treatment was performed in 2 stages. Stage 1 was aimed at the biliary system decompensation, elimination of infection, withdrawing from the state of hepatic insufficiency, and at the 2nd stage a permanent bile passage was restored with surgical intervention or endobiliary ones.

The findings on performance of intervention technique, versions and ways of endobiliary interventions will be presented in the report.

### P0183 ROLE OF INTRAOPERATIVE ANGIO-ECHOGRAPHY AT HEPATIC SURGERY

Ken TAKASAKI, S.KOBAYASHI, A.SAITO, Y.SHIMIZU, A.YAGAWA, T.TUGITA, A.ARUGA, T.HAYASHI,S.MIYAZAKI,K.KATURA,T.SUZUKI, F.HANYU.
Institute of gastroenterology Tokyo Women's Medical College.

In Japan, about 80% of hepatocellar carcimona(HCC) are associated with liver cirrhosis. Then the range of hepatic resection must be limited in consideration of remaining liver function.

On the other hand ,it is thought that, in case of HCC,intra hepatic metastases are formed through the portal vein in other area.

So at the operation, the area which include the tumor must be systemically completely removed, to improve the curability even a small tumor. For these purpose, we developed a method of hepatic resection performing hilar preparation of portal vein as a unit of Glissonean code.

Using this method, we can transect any one of intrahepatic trifling branch of portal vein selectively.

At the operation,it is necessary to be confirmed the distribution of small satellite nodules around the tumor,if any, and the trifling branch of Glissonean code which feed the tumor.

We developed arterial and portal angio-echography under $CO_2$ gas injection into the vessels.

By these examination, we can confirm the trifling branch which include the tumor and satellite nodules, and also can measure the distance of surgical margin.

### P0184 PARTIAL PERICYSTECTOMY VERSUS CAPITONNAGE IN LARGE HEPATIC ECHINOCOCCUS CYSTS

A. Krasniqi, R. Jashari, R. Binishi,
I. Dreshaj, D. Limani, A. Rrusta
Clinic of Surgery, Medical Faculty,
University of Prishtina, Yugoslavia

Recently in the treatment of hepatic echinococcus cysts total or partial pericystectomy is prefered to other methods such are marsupialization or capitonnage (Papadimitrios 1970, Belli 1986).

The aim of this paper was to present our experience in the treatment of large hepatic echinococcus cysts. As large cysts were considered those which involved more than three liver segments and had communications with biliary tract. In five year period (1982-87) 65 patients with hydatid cysts were treated; 32 of them had large cysts. Cyst daughters in common bile duct and choledochus were found in 11 (34.4 %) of patients with large ones.

Two methods of treatment were used: 1. cystectomy followed by capitonnage of cavity (12 patients), and 2. cystectomy followed by partial pericystectomy, cholecystectomy, exploratory choledochotomy (removal of eventual cyst daughters), T drainage with intraoperative cholangiography, and omentoplication by Papadimitrios (20 patients). Saline solution was injected through T tube to identify biliary openings on the cavity surface, and after that smaller openings were carefully sutured, whereas the eroded larger ducts were continually sutured using vicryl 5.0. The perioperative mortality rate was zero.

After the analysis of each patient treated with first method, one retention of cyst daughter in choledochus and one recidivation of hepatic cyst were recorded. Patients treated with second method had longer hospital stay, but no cyst retention nor recidivation were found.

These results suggest that in the treatment of large hepatic echinococcus cysts (where total pericystectomy is infeasible) the second method has better outcome and it provides better possibilities for postoperative follow-up.

References:

Papadimitrios J, Mandrekas A. Br J Surg 1970: 57:431.
Belli L, et al. Surg Gynec Obstet 1986: 163:127.
Hashemian H. Abdominal operations 1980: 1329, New York

**P0185** THE INTERMEDIATE RESECTION OF THE PANCREAS.

D. Marrano, O. Campione, V.M. Greco,
R. Casadei, F. Bezzi, P. Piva.
Istituto di 1^ Clinica Chirurgica della
Università degli Studi di Bologna (Dir.:
Prof. D. Marrano).

The intermediate resection of the pancreatic body is performed to avoid wide excision of the glandular parenchima reducing, therefore, the incidence of secondary diabetes and exocrine pancreatic insufficiency.
The indications for this type of operation are: 1) trauma of the pan creatic body; 2) benign tumors, both endocrine and exocrine, for which an enucleation or enucleoresection are not possible.
The technical procedure consists of a destructive fase, with excision of the pancreatic body, and a reconstructive fase regardind the proximal and distal slices.
The proximal cephalic slice can be closed manually or by a mechanical suturer (T.A. 55), while for the distal slice a termino-terminal anastomosis with the jejunum on Roux-en-y loop is usually done.
Our experience comprises 2 cases of intermediate resection for a non-functioning endocrine tumor and for a glucagonoma.
The limited indications justify the numerical scarsity of this operation in our case histories and in the data emerging from a revision of international literature.

P0186 5'-NUCLEOTIDASE ACTIVITY
A NEW MARKER OF LIVER GRAFT VIABILITY.

T.van Gulik, R.Nio, W.Frederiks, T.Karsten,
F.Marx, P.Verbeek, J.Vaillant, N.Lygidakis,
J.Ringers, L.deWit, M.v/d Heyde, P.Klopper.
Dept.of Surgery, Academic Medical Center,
University of Amsterdam, The Netherlands.

In liver transplantation, there is a need for markers which can accurately predict viability of a preserved liver graft prior to transplantation. 5'-Nucleotidase (5'-NT) activity in the bile canalicular membranes is a sensitive parameter of ischemic liver cell damage. We are assessing the localization of bile canalicular 5'-NT activity as an indicator of preservation induced injury in cold stored canine livers. Our initial results are reported.

Canine liver biopsies are incubated during 120 min. at 37$^{o}$C and 4$^{o}$C resp. 5'-NT activity is scored in cryostat sections by means of a histochemical assay. The scoring system is based on the sharpness and intensity of the stained bile canalicular membranes. 5'-NT activity is expressed as percentage of the initial (in vivo) value. 5'-NT activity decreased to 0% and 77-83% resp. after normothermic (37$^{o}$C) and hypothermic (4$^{o}$C) ischemia.

In mongrel dogs, the liver is flushed in situ with cold (4$^{o}$C) UW-solution (UW) and Euro-Collins solution (EC) by cannulating the right and left portal vein separately. Left and right liver lobes are subsequently cold stored (4$^{o}$C) in either solution. After 48h preservation time, biopsies (5x5mm) are taken from peripheral sites and 5'-NT activity scored. 5'-NT activity decreased more rapidly in livers cold stored in EC (0-6%) as compared with UW (37-39%) and can be associated with the lower preservation tolerance known of EC preserved liver grafts.

A canine liver was cold stored in UW-solution. After 24h preservation time, 5'-NT activity had decreased to 60%. The liver was orthotopically transplanted and 1 hour after reflow, 5'-NT activity was 49% (reperfusion trauma?). Immunosuppressive therapy was omitted. The dog survived and serum aspartate transaminase (AST) values had returned to near-normal by p.o. day 3. A biopsy was taken on postoperative day 6 in which 5'-NT activity was 24%. AST at that time showed an elevation suggestive of rejection.

The determination of 5-NT activity provides a simple and sensitive test to assess graft viability and this method is further explored in relation with preservation-reperfusion studies and rejection phenomena.

**PO187** DIAGNOSIS AND SURGICAL MANAGEMENT OF LIVER HYDATIDOSIS.

A. Rigas, M.Digalakis,G.Papoutsis,
K.Kilakos, S. Fournogerakis.
Tzanion General Hospital of Piraeus.
1st Surgical Unit.

The last 20 years 318 patients with hydatid disease of the liver have been treated in our surgical unit.

Diagnosis was made using clinical criteria, skin tests, serology and imaging techniques. In 228 patients the cyst was not complicated (Group A). In 90 patients the cyst was infected or communicated with the biliary tract (Group B).

We performed the following types of operations: a)partial cyst resection (185 patients),b)Complete cyst resection (67 patients),c)Right lobectomy (1 patient),d) Evacuation, irrigation and drainage (65 patients).

Postoperative complications were: a) External biliary tract fistula (35 patients), b) Liver abscess (3 patients), c) Peritonitis (8 patients), d) Wound infection (13 patients),e) Pneumonia (3 patients), f) Death (1 patient). There was no significant difference in complication rates between groups A and B.

The lower postoperative hospital stay was 11 days for the patients with evacuation and omentoplasty and higher for the patients with marsupialization (22 days).

We believe that partial cystectomy with omentoplasty, or evacuation, irrigation and omentoplasty is the procedures of choise in the treatment of hydatid disease of the liver

**P0188** CHEMICAL AND CRYSTALLOGRAPHIC STUDIES ON CALCIUM CARBONATE GALLSTONES

S.HUANG,C SU,W LUI,C.WU
General Surgery,Veterans General Hospital
Taipei,Taiwan,R.O.C.

Between August 1986 and December 1987,28 cases of calcium carbonate gallstones were chemically analyzed by atomic absorption photospectrometry and crystallographically studied by the X-ray powder diffraction method and infrared absorption spectroscopy ( KBr method ).
Chemically,calcium carbonate was the major constituent,ranged from 36.0% to 82.5% and averaged 64.5%.Crystallographically,calcium carbonate has three different polymorphic crystalline forms: calcite, aragonite and vaterite.In nature the most stable calcite (hexagonal) is most commonly found and aragonite(RHOMBIC) is next .On the other hand vaterite, which is unstable hexagonal modification, rarely occurs in biological systems.

But in our gallstones series in man, aragonite was most commonly found,with an occurrence rate of 82.1%, while that of calcite was 57.1%, vaterite 35.7%. Moreover, 8 cases contained all three forms of calcium carbonate polymorphs: calcite,aragonite and vaterite. This was a very unusual condition.

References:

Saito K, Omori H. Gastroenterologia Jpn 1986; 21:162
Lowenstam H. Science 1975; 182:363
Lagergren C. Acta Chir Scand 1962; 124:320

P0189 A SIMPLIFIED PROCEDURE FOR INTRAOPERATIVE RADIATION THERAPY IN THE TREATMENT OF PANCREATIC CANCER.

A. Zerbi, M. Carlucci, R. Castoldi, D. Parolini, V. Fossati, C. Staudacher, F. Volterrani, V. Di Carlo-Pat. Chirurgica, IRCCS San Raffaele, University of Milan, Italy.

A widespread use of intraoperative radiation therapy (IORT) in the treatment of pancreatic cancer is hindered by technical drawbacks as the availability of operating rooms by the department of radiotherapy or the presence of a linear accelerator in an operating room.

In San Raffaele Institute a simplified IORT procedure was developed, by using a methylmethacrylate cylinder placed on the tumor mass or tumor bed. The skin is then sutured around the superior border of the cylinder itself thus avoiding successive closures and reopenings of the incision in the linear accelerator suite, after the transport through isolated but nonsterilized corridors and elevators. The treatment cone of the linear accelerator gantry is telescoped perpendicularly inside the cylinder and the appropriate radiation dose is delivered, while the patient is continously monitored by closed-circuit television.

In the last 3 years 23 patients underwent IORT at our Department for pancreatic cancer located in the head (18 cases) or in the body (5 cases). IORT was associated to biliary and/or gastric by pass in 7 patients, and as adjuvant radiation therapy followed in 10 cases a pancreatoduodenectomy. Unresponsive pain related to the pancreatic tumor was present in 9 of the 23 patients. Radiation doses from 1500 to 3000 cGy (mean 2000 cGy) with electron beams energies between 8 to 22 MeV were delivered. No surgical infection or other complication IORT-related was observed. A complete relief of pain was observed in 6 of 9 cases (66.6%). Median survival in treated patients is 9 months (7 patients alive).

In conclusion IORT according to this procedure resulted to be easy and safe. Preliminary results suggest a promising role of IORT in local control of unresectable pancreatic cancer and in adjuvant treatment after resection.

### P0190 RARE ANOMALY OF BILIARY TRACT AS A CAUSE OF IATROGENIC INJURY DURING CHOLECYSTECTOMY

Dr. Karel Jaros
Surgical Department Bulovka,
Prague, Czechoslovakia

This abstract describes a rare anomaly of a biliary tract, when the right and left hepatic ducts are entering independantly into the gall bladder.

The hepatic ducts were accidently cut during cholecystoctomy. At first the injury was treated by provisional drainage through a silactic stent placed into the common bile duct.

Post operative stricture of the hepatic ducts was finally resolved by biliary-enteric anastomosis.

The author describes his experience with reconstruction of iatrogenic strictures in 10 of his patients.

Also described are the limiting factors in the treatment of recently occurred injuries and the treatment of later strictures of biliary tracts.

References:

Bismuth H., Franco D., Corlette M.B., Hepp J,: Long Term results of Roux-en-Y hepaticojejunostomy. Surg Gynec Obstet, 146, 1978, 161-167.

Blumgart L.H. Kelley C.J., Benjamin I.S.: Benign bile duct strictures following cholecystectomy: critical factors in management. Brit J Surg, 71,1984,836-843.

Hepp J: Hepaticojejunostomy using the left biliary trunk for iatrogenic biliary lessons: the french connection World J Surg, 9, 1985, 507-511

P0191

### BACTERIAL INFECTION AND CHRONIC PROLIFERATIVE CHOLANGITIS OF THE INTRAHEPATIC BILE DUCTS IN DOGS

T.Nakamura,T.Nagakawa,T.Ohta,N.Ueda,N.Kadoya,
H.Kobayashi,Y.Nakano,K.Maeda,M.Kayahara
K.Ueno,I.Konishi,T.Matsumoto,I.Miyazaki
The Second Department of Surgery, School of Medicine,Kanazawa University, Kanazawa,Japan.

Morphological changes of the intrahepatic bile ducts and hepatic parenchyma after loading bile stasis and bacterial infection of the biliary tract on partial lobe of the liver of mongrel dogs were studied in order to examine the pathophysiology of the hepatolithiasis. Three groups of dogs were prepared by cannulation to the bile duct branch draining the left lateral two lobes of the liver and were treated as follows:Group 1, the cannula was obstructed after injection of $10^7$-number E.coli and $10^7$-number B.fragilis;Group 2, the cannula was obstructed after injection of E.coli only; Group 3,the cannula was obstructed without injection of bacteria. 3 months later, livers of sacrificed dogs were morphologicaly examined. In Group 1 dogs, epithelial adenomatous hyperplasia was noted in association with chronic proliferative cholangitis which was characterized by moderate to severe periductal fibrosis with a large number of inflammatory cells and mucus producing glandular elements of the bile duct wall. These findings are found most frequently in human hepatolithiasis. In Group 2, periductal fibrosis was severe, but epithelial hyperplasia or proliferation of glandular element was little found. In Group 3, there was no finding of periductal fibrosis, epithelial hyperplasia, or mucous glands. Hepatic atrophy of Group 1 and 2 dogs was significantly severer than that of Group 3 dogs. From the above results, it is suggested that chronic proliferative cholangitis may be raised by bacterial infection (especially by anaerobic bacterial infection) of the biliary tract, in addition to bile stasis.

### P0192 LYMPHOPROLIFERATIVE DISORDERS AFTER LIVER TRANSPLANTATION (OLT) - A RECENT EXPERIENCE

J.C.Emond, T.G. Heffron, J.R. Thistlethwaite, X. Rogiers, O.I. Olopade, C.E. Broelsch
University of Chicago, Chicago, Illinois, USA

Lymphoproliferative disorders (LD) have been recognized as a complication after organ transplants. Past incidence has varied by immunosuppressive regimen and ranges from 1-5%. It has been suggested that the recent use of lower doses of cyclosporine (CY) might result in a lower incidence of this complication. To assess this we reviewed our experience with LD in 230 patients receiving OLT since 1985.

Patients & Methods: 230 patients received 289 grafts between 1985 and 1990. All patients were managed using standard protocols based on CY and steroids +/- azathioprine (AZA). CY therapy was based on blood levels accepting therapeutic levels HPLC 150-200. Solumedrol and OKT3 were used for control of rejection.

Results: Six liver transplant patients (2.6%) developed LD including 4 children (3.6%) and 2 adults (0.9%). Median time of onset was 3 months. Two of 6 patients developing LD had acute hepatitis as the cause of liver failure. Sites of origin were tonsils and adenoids (n=1), ethmoid sinus (n=1), cervical lymph nodes (n=1), and three in liver graft (n=3). One lesion in the hepatic hilus presented as a biliary stricture. All LD were of B-cell origin: 5 monoclonal and 1 polyclonal. Rejection treatment was required in 4 (67%), OKT3 in 2 (33%), CMV infections occurred in 5 (83%). Comparable frequencies in other OLT patients were 78%, 15%, and 22%. Outcomes: One pediatric patient with a polyclonal LD after 9 months responded promptly to reduction in immunosuppression and is asymptomatic after 22 months. Four of 5 with monoclonal LD were treated by reduction in immunosuppression and one by chemotherapy (CHOP). Only one presented late (24 months) and all 5 died within 4 months of diagnosis associated with multiple septic complications.

Discussion: These results demonstrate that LD continues to be a risk despite the modifications in the use of CY. Children may be more susceptible. LD occurred very early with a virulent course and death in 5 of 6 cases. The association of CMV and sepsis suggests a context of global immune failure.

P0193

### ROLE OF PRE-OPERATIVE US IN GUIDING HEPATIC SEGMENTECTOMY: variations in blood supply of right lobe

C. Martinoli, G. Cittadini jr., R. Conzi, G.B. Arnone
U. Cosce and F. Griffanti Bartoli
Institute of Radiology "R" and Surgical Pathology "B"
University of Genoa, Italy

In segmental right hepatectomy it is difficult to guide exactly the resection because of the lack of precise anatomic landmarks on the liver surface between 5th, 6th, 7th and 8th segments. In order to preserve completely, when possible, the adjacent segments around selective resection, traditional techniques based on Couinaud map consist of identification and ligation of vascular pedicles after detaching the hilar plate. This ligation creates complete ischemia of segmental groups supplied alternatively by right paramedial (5th and 8th segments), right lateral (6th and 7th segments) or left vascular (2nd, 3rd and 4th segments) branches, producing a change in colour which allows a nearly bloodless division of the liver. In order to isolate single segments (critical in the right lobe) from the surrounding ones, and namely 5th from 8th, and 6th from 7th, the recent introduction of operative ultrasound has made possible limited resections by guiding clamping of the appropriate distal vascular pedicle, while preserving adjacent functioning parenchyma and so reducing the risk of post-operative hepatic failure. Really, an accurate preoperative ultrasonography may provide the same precise vascular mapping of the liver, thus allowing to determinate volumetric segmental relations, vascular anomalies, atipical branching, shunts, vascular wall infiltrations and thromboses, in order to planify the successive resection on operative ultrasonographical basis.

The aim of the present study was to analyze the principal anomalies in vascular distribution of the right lobe by means of the use of pre-operative ultrasonography. The main variants frequently observed were: i) inconstance of right paramedian branch, with 5th and 8th segmental pedicles arising from a single large sized right lateral truncus; ii) double or multiple segmental branching to 6th and 7th segment from the right lateral portal pedicle, and iii) exclusive or double vascular perfusion of the 8th segment from paramedial and/or lateral truncus. Results are discussed vs Trinh Van Minh' data.

PO194 PANCREATIC MUCINOUS DUCTAL ECTASIA (PMDE) surgical problems about 4 operated cases

B. SASTRE,J.SAHEL,MJ.PAYAN,JC. SARLES, S. AGOSTINI Hopital Ste Marguerite, 270 bd de Ste Marguerite 13009 - Marseilles (France)

Four cases of PMDE, localized 3 times, diffuse once, covered by a muco-secreting epithelium as described by ITAI and al (1) were operated on in 1 year. The patients (2M,2F;30-72 years) had abdominal pain in 3 cases with in two antecedent of acute pancreatitis access ; jaundice revealed the fourth case. ERCP always showed ampullary dilatation of the pancreatic ducts, filled with radiotransparent casts, joining up a Wirsung duct dilated below . those lesions were located to the head twice, to the body and tail once and were diffuse in the last case. Pathological examination confirmed the diagnosis and brought to evidence in one case in-situ malignant transformation.2 pancreaticoduodenal resections (PDR), one caudal pancreatectomy and one subtotal-PDR were performed. If resection of the located lesions must be achieved because of potential malignant transformation, the type of surgical digestive reconstruction after PDR may be discussed. It seems better, in this particular case to perform a pancreato-gastric anastomosis than a pancreato-jejunal one in order to allow post-operative ERCP of the remaining tail. In diffuse cases must we have to do, even in young patients, a total PDR in spite of its metabolic effects, or is it licit to achieve, in obviously untransformed cases, a subtotal-PDR to preserve the endocrinal function, as it was done once here? Type, frequency and duration of the survey which is imposed by partial pancreatic resection for PMDE are yet imprecised and discussed.

(1) ITAI and al Radiology, 1986,161, 697-700

**PO195** BIOHUMORAL AND RADIOLOGICAL FINDINGS IN FOCAL NODULAR HYPERPLASIA( FNH )OF THE LIVER

G.MANGIANTE,FG MUSAJO,A MARABINI,PG GIORGETTI
G BENATI,L MARCHIORI,A ASNICAR,C PROCACCI,
N NICOLI,A DAGRADI
CLINICA CHIRURGICA UNIVER.OF VERONA-ITALY

From 1970 to 1989 29 FNH in 29 pts were diagnosed at our Institute. All the pts underwent complete liver biohumoral screening,that always was negative,except for a serum rise of gammaGT in 8 pts(27.6%) No patient with elevate gammaGT had a concomitant liver disease or history of alcohol intake or enzyme inductor drugs:only in 1 patient were an history of oral contraceptive intake for 24 mo.7 pts with rised gammaGT underwent resection of their FNH with prompt normalization of the serum value of the enzyme,while in the not resected patient the enzyme is still above the normal range.
US were performed in 27 pts,CT in 21,angiography in 14,Tc99m-colloid scintigraphy(HCS) in 14 pts and Tc99m-HIDA scintigraphy(HBS)in 13 pts.The results of these tests are summarized in the following table:

TABLE

| | n.pts | no mass | focal les | FNH diagnosis |
|---|---|---|---|---|
| US | 27 | 3 | 21 | 3 |
| CT | 21 | 3 | 4 | 14 |
| ANG | 14 | 0 | 6 | 8 |
| HCS | 14 | all no focal defects | | |
| HBS | 13 | 1 | 0 | 12 |

The diameter of the FNH midiagnosed both by US and CT was between 0.8 and 1.3 cm.Only 1 FNH of 0.9 cm of diameter located near a fibrolamellar HCC was not detected by HCS.
We never observed false positive diagnoses of FNH:expecially HBS in other 32 pts that underwentthe exam for liver masses never demostrated lesional cholestasis.
From our experience we now believe that CT diagnosis of FNH combined with no focal defects at HCS and lesional cholestasis at HBS are sufficient to make a correct diagnosis of FNH if the oncological markers are negative.

**P0196** CLINICAL SIGNIFICANCE OF PERCUTANEOUS TRANSHEPATIC GALLBLADDER DRAINAGE FOR ACUTE CHOLECYSTITIS

T.Itoh, Y.Bandai, K.Shimada, Y.nomura, K.Shimomura, Y.Iishizaki, S.A.Nayeem, Y.Idezuki
Second Department of Surgery, Faculty of Medicine, University of Tokyo, Tokyo,Japan.

In patients with acute cholecystitis, percutaneous transhepatic gallbladder drainage ( PTGBD ) can diminish the indications for emergent operation.

From 1979 to 1989, we performed ultrasonically guided PTGBD in 72 patients. The indications for PTGBD were obstructive jaundice in 39 cases and severe acute cholecystitis in 33 cases. In 33 patients with acute cholecystitis, 13 patients were postoperative cases and 3 patients were complicated with severe liver cirrhosis. Drainage tube was inserted via the percutaneous transhepatic route by Seldinger's method.

In all cases with acute cholecystitis, PTGBD was successfully performed without severe complications. Bile leakage and shock were not experienced. After PTGBD, almost every patients were recovered well immidiately. One patient died due to multiple organ failure but cause unrelated to PTGBD. All poatoperative cases were diagnosed as noncalculous cholecystitis, therfore, after recovery cholecystectomy was not indicated. Postoperative acute cholecystitis is not so rare as previously considered. Diagnosis can be maken by ultrasonography. In high-risk patients such as Child C liver cirrhosis, PTGBD must be especially good method for therapy.

### P0197 CEPHALIC DUODENOPANCREATECTOMY IN THE TREATMENT OF DEGENERATED DUODENAL VILLOUS ADENOMAS.

M. Moreno Azcoita; J.M. Fradejas; J.M. Jover; P. Gomez Maestro; J.L. Ramos; J. Alvarez.

Hospital Central Cruz Roja. Madrid. Spain.

Villous adenomas (VA) are epithelial neoplasias that can appear in all the digestive tract, although its most frquent location is colo-rectal . Duodenal villous adenomas (DVA) are located in the upper digestive tract, where they represent 1% of the overall duodenal tumours.

Four patient are presented in this poster. They have been diagnosed and treated in our Department. The VA were located in the ampullary area in 3 cases, and in the third duodenal portion in fourth case.

From the clinical point of view symptomatology was different in each of them, since the first appeared with obstructive jaundice due to papillary compression (it had associated choledocholithiasis); the second case presented as chronic anemia due to G.I. bleeding; the third case became evident through a syndrome of malabsortion; and the fourth case started with upper bowell obstruction.

The most important factor that determines prognosis and treatment for these neoplasias is the risk of malignánt degeneration (higher than 60%). In three of our cases carcinomatous degeneration has been produced: one case was irresectable and two were treated with the WHIPPLE S procedure, with pyloric preservation in one. One of these latter patients remains asymptomatic three years after CDP, and the other has a relapse one year after operation.

We considerer that resection must be attempted in all VDA in order to prevent malignant risk on the one hand and, on the other hand when carcinoma has already appeared, surgery must be performed with oncologic criteria, since this resection allows survivals superior to other duodenal and periampullary neoplasms.

### PO198 HEPATIC TRAUMA - ANALYSIS OF 368 CASES

E. Ramos, J. Andrade, A. Pereira,
G. Costa, J. Pena. Hospital S José,
Lisboa, Portugal

From November 1981 until December 1988, 368 patients with Hepatic Trauma were operated on in the Emergency Surgical Unit of Hospital S José in Lisbon. In this retrospective study the authors analyse the results and prognosis based upon the etiology, type and severity of the hepatic wounds, the presence of associated lesions and the different surgical approaches.

They conclude that the mortality is higher in blunt injuries, hepatic wounds involving main vessels, existence of more than 3 associated lesions and the performance of hepatic resections.

P0199

### EFFECT OF INJECTION OF PROLAMINE INTO MAIN PANCREATIC DUCT IN EXPERIMENTAL ACUTE PANCREATITIS

Kazuaki Shimada, Tohru Itoh, Makoto Takami,
Yuji Nomura, Kazuyuki Shimomura, Youichi Ishizaki,
Yasuo Idezuki.
Second Department of Surgery, Faculty of Medicine,
University of Tokyo, Tokyo, Japan.

The effect of prolamine injection into the pancretic duct in experimental acute pancretitis has been studied.

Acute hemorrhagic pancreatitis was induced by the administration of 10% deoxycholate (0.4ml/kg) into the main pancreatic duct in mongrel dogs. Animals were divided into two groups. Group I (n=5) was non-treated control. Group II (n=5) was treated with injection of prolamine (0.05ml/kg) into main pancreatic duct 10 minutes after inducing pancreatitis.

In group I, the mean survival term was 2.6±1.0 days and all dogs died within 4 days. Autopsy findings showed acute hemorrhagic pancreatic necrosis.

In group II, all dogs were alive over 5 days. Microscopic findings showed improvement of acute pancretitis and chronic inflammation.

The survival term in group II was prolonged and it suggested that injection of prolamine have possibility of preventing progression of acute severe pancreatitis.

P0200 SURGICAL TREATMENT OF CARCINOMA OF THE BILIARY CONFLUENCE

Nicoli N., Iacono C., Mainente M., Marchiori L.,
Mangiante G., Dagradi A.
Departement of Surgery
University of Verona ,Verona - Italy.

Carcinomas of the confluence of the major hepatic ducts shows surgical problems either concerning radical or palliative treatment.

Althought in rare series resectability is high (Mizumoto 1986), the majority of the Authors refers thats only 15-20%of these patients may undergo radical surgery (Bismut 1988).

Between 1970 and 1989 we have treated 84 patients with carcinoma of the confluence of the major hepatic ducts. Curative resections were carried out in 16 cases (18%); operative procedures were as follow : 14 major hepatic resections of the biliary ducts confluence (5 extended right lobectomies associated caudate lobe lobectomy, in one case with partial portal resection and in another one with partial inferior vena caval resection, 2 right epatectomies associated caudate lobe lobectomy, in one case with portal replacement, 7 left hepatectomies of which 3 associated caudate lobe resection) and 2 major hepatic resection when the lesion was located in left or right ducts without affecting the confluence. Among 14 patients who underwent liver and biliary resection, 3 died within 30 days from surgery and 2 within 2 and 3 months respectively due to postoperative complications.

Six patients survived 13,20,24,36,36,41 months respectively (mean 28,3 months) and 3 are alive respectively 2,9,18 months after surgery.

The 2 cases treated with hepatic resection alone had recurrence and required PTBD at 13 and 18 months respectively, with the overall survival of 25 and 30 months.

The remaining 68 cases (82%) underwent palliative procedures: 32 PTBD, only 1 laparatomy with biopsy and 35 palliative surgery : 7 biliary T-tube drainage, 2 palliative resections of the confluence, 1 left hepatic-jejunostomy, 25 intrahepatic cholangiojejunostomies , 16 at III° segment, 1 at VI° segment and 8 both.

Operative mortality of intrahepatic surgical by-passes was 4%. Mean survival was 14 months (range 3-58 months) and good decrease of jaundice and itching was noted in 21 patients (84%), in 4 patients (16%) palliation was complicated by cholangitis.

In our experience tumor resection associated with liver resection constantly including caudate lobe resection is the treatment of choice in selected cases.

As to the patients with non resectable confluence tumor, among the palliative procedures, intrahepatic cholangiojejunostomy (if possible with Soupault-Couinoud procedures) can provide temporary retourn to normal life.

Alternatively PTBD relieves the symptoms.

References :

Bismut H., Cartaing D., Trayner O. : World J. Surgery 1988, 12 (1),39.
Mizumoto R., Kawarada Y., Suzuki H.: Surg. Gynecol. Obstet. 1986,162 : 153.
Soupault R., Cuoinaud C: Presse Med. 1957, 65 : 1157.

P0201 **IMPACT OF SURGICAL TREATMENT ON UICC CLASSIFIED PANCREATIC CANCER PATIENTS**

J. W. Heise, H. Becker, F. Borchard, M. Krüger, H.-D. Röher. Department of Surgery, Heinrich-Heine-Universität, Düsseldorf, FRG.

Poor prognosis of well defined adenocarcinoma of the pancreas requires superb selection of candidates who are likely to benefit from radical surgery. The most recent 1987 UICC classification provides a generally accepted tool for stratifying those patients. Aim of the study was to apply the UICC key to a recent group of patients treated operatively for pancreatic cancer and to evaluate the impact of surgical treatment on survival.

From 4/86 to 11/89, 82 patients underwent laparotomy for adenocarcinoma of the exocrine pancreas, either for resection, bypass procedures, or exploration only. Staging was prospectively performed according to the 1987 UICC classification. Survival was calculated as both actual and actuarial 1 year rates.

Thirty-four resections, 42 bypass procedures, and 6 explorative laparotomies were carried out. Overall actual and actuarial survival at 1 year was 26.9% and 24.2%, respectively, with 43.3% and 42.1% after radical surgery, 16.1% and 13.0% after palliative surgery, and none patient alive at 1 year after exploration only. Eight out of 16 (50%) node positive Stage III patients actually lived at 1 year following resection but only one out of seven (14%) post palliative surgery.

We conclude that in spite of selection by other factors like age and T status, a distinct group of patients with pancreatic cancer benefits from radical surgery, even in the presence of lymph node involvement. Thus under reasonable circumstances the resectable tumor should be removed without regard to lymphatic spread.

**P0202** AN 8 YEAR STUDY OF CARCINOMA OF THE GALLBLADDER

CD Johnson, NJ Carty. Southampton General Hospital, Southampton. UK.

Carcinoma of the gallbladder is an uncommon condition ($1.3/10^5$ in our district). It causes few symptoms, and if it is not detected incidentally at an operation for gallstones, it usually presents with advanced disease. This study is a retrospective review designed to determine the clinical pattern of presentation and to identify ways in which management might be improved.

From pathology records we identified all patients in our district diagnosed in 1982-85. The Regional Cancer registry identified all known cases for 1983-86. 134 cases were identified. Case notes and follow up data were available for 95.

Median duration of symptoms was two months (right upper quadrant pain 68%, weight loss >3kg 56%). There was a palpable mass in 66%. Six of 41 jaundiced patients had localised tumour with stones in the CBD. Anaemia was present in 18%.

Preoperative investigations were unhelpful. The diagnosis was made at operation in 69 of 81 patients operated on. Nevin stage (1) was I:1, II:1 III:19, IV:15, V:59. Of 10 patients with stage III tumours in whom the cause of death was known, 8 died from tumour recurrence. Median survival for stage III was eight months stage IV seven months, stage V 1.5 months.

This study confirms the very poor prognosis of carcinoma of the gallbladder. Many patients have lymph node disease (stage IV). Nevin stage V includes some patients with direct invasion of the liver, as well as those with distant hepatic metastases. Aggressive local surgery might improve the outlook for patients in Stage III and IV (2). Patients with local liver invasion only should be reclassified as group III B, to emphasise the possible application of extended resection in their management.

1. Nevin JE, Maran TJ, Kay S, King R. Cancer 1976, 37: 141-8
2. Nakamura S, Sakagachi S, Suzuki S, Muro H. Surgery 1989; 106: 467-73

**PO203** SURGERY FOR COMPLICATIONS OF INJECTION SCLEROTHERAPY FOR GASTRO-OESOPHAGEAL VARICEAL BLEEDING

N.D. Heaton, D. Westaby and E.R. Howard.
King's College Hospital, London. UK.

Injection sclerotherapy is of proven value for the acute and long-term management of variceal bleeding. Surgical treatment in our unit is now largely restricted to patients bleeding from sclerotherapy induced oesophageal ulceration or ectopic varices.

Between 1985 and 1989 34 patients (age range 1.5 - 83 years) have received surgery following injection sclerotherapy. During this time 268 new patients were admitted with variceal bleeding. A total of 324 adult and 252 children with portal hypertension were under review during this period.

Nine children (3.6%), 5 with intra-hepatic disease (IHD) and 4 with portal vein obstruction (PVO) have been treated for bleeding and hypersplenism. 2 children had bled from oesophageal ulcers, and 5 from varices at ectopic sites:- stomach (2), duodenum (1), jejunum (1), unknown (1). All 9 were treated by portal-systemic shunts with no mortality or significant morbidity.

Twenty-five adults (7.7%) (IHD-20, PVO-5) were treated for uncontrolled bleeding. Portal-systemic shunts were constructed in 12 for oesophageal ulceration and in 2 for ectopic bleeding (stomach and common bile duct). Devascularisation procedures were used in 13 patients for oesophageal ulceration (4), ectopic varices (8) and aorto-gastric fistula (1).

| Results of surgery | | Patients | Alive |
|---|---|---|---|
| Devascularisation procedures | | 13 | 8 |
| Portal-systemic shunts | (a) Children | 9 | 9 |
| | (b) Adult | 12 | 9 |

Failures of sclerotherapy are few. Surgery, using devascularisation or portal-systemic shunts proved to be successful as a 'rescue' measure.

### P0204 MANAGEMENT OF HEPATIC METASTASES IN THE UK

CJ Cahill Westminster Hospital, JA Pain King's College Hospital, London UK.

A postal questionnaire was sent to a random sample of 25% of all UK General surgeons (236 surgeons). 218 (92%) replied. 213 managed colo-rectal disease.

81 (38%) had resected, or referred for resection, patients with appropriate colo rectal liver metastases during the previous five years.

If resection is inappropriate 15 (7%) often refer patients for chemotherapy, 95 (47%) sometimes do, but 84 (39%) did not answer the question, and 19 (9%) never treat multiple liver secondaries. Twelve per cent of those using chemotherapy use regional therapy, 76% systemic, and 12% both.

UK surgeons appear to be moving towards surgery for some colo-rectal metastases, but little change has occurred in the acceptance of chemotherapy. Our findings are similar to those of Karanjia et al (1) for surgery, but we obtained lower figures for actual administration of chemotherapy than the previous survey which asked if chemotherapy would be considered.

(1) Karanjia ND, Rees M, Schache D, Heald RJ. Hepatic resection for colorectal secondaries Br J Surg 1990; 77: 27-9

### P0205 Clinicopathological study of three cases of solid and cystic tumor of the pancreas, especially about immunohistochemical and electron microscopic findings

N.Ueda, N.Kadoya, T.Nakamura, Y.Nakano, H.Kobayashi, K.Maeda, M.Kayahara, T.Ohta, K.Ueno, T.Nagakawa, I.Miyazaki[1], M.Kurachi[2], I.Konishi, T.Hirono[3], T.Akiyama, H.Sodani[4], H.Yamazaki, K.Hirano[5]

[1]Dep. of Surgery (II), Kanazawa University, School of Medicine [2]Dep. of Surgery, Houju Hospital [3]Dep. of Surgery, Toyama Civic Hospital [4]Dep. of Surgery, keiju Hospital [5]Dep. of Surgery, Kaga Central Hospital

Case1:A 37-year-old woman was pointed out to have an abdominal fist like sized calcification at group examination. The diameter of the tumor at the tail of the pancreas was 7.5×7.5cm. Splenectomy and distal pancreatectomy were performed.
Case2:A 39-year-old man was pointed out to have a pancreatic tumor with calcification on ultrasonography at a clinical survay. The diameter of the tumor at the body of the pancreas was 7.5×4.5 cm. A total pancreatectomy was performed.
Case3:A 16-year-old girl was admitted with the chief complaint of upper abdominal pain. Computerized tomography demonstrated a cystic mass at the tail of the pancreas. The diameter of the tumor at the tail of the pancreas was 6.0×5.5cm. Splenectomy and distal pancreatectomy were performed.
Three patients have now been followed for from 5 to 4 years with no evidence of recurrence.
Macroscopically, three tumors appeared encapsulated and the cut surface displayed a hemorrhagic and necrotic appearance. Microscopically, all demonstrated two distinct types of cellular arrangement:a solid and a papillary pattern. Immunohistochemically, alpha-1-antitrypsin was detected in three cases, but tumor makers, such as CEA, CA19-9, DupanII, pancreatic hormonal makers and neuron specific enolase were not detected in all cases. Electron microscopy revealed tumor cells containing abundant mitochondria in all cases and zymogen like granules in two cases, but not revealed neurosecretory granules in all cases.

### PO206 GASTRODUODENAL ULCER PERFORATION IN CIRRHOTIC PATIENTS

C. Vons, O. Farges, H. Monnier, J. Belghiti. F. Fékété.
Service de Chirurgie Hopital Beaujon 92118 Clichy France

In cirrhotic patients, diagnosis of gastroduodenal ulcer perforation is difficult and prognosis of surgical treatment is poor. The aim of this work was to evaluate retrospectively elements of diagnosis, duration to surgery and results of surgical treatment in 22 patients (Pugh A : 5; Pugh B : 10; Pugh C: 7). Incidence of acute abdominal pain, rebound tenderness and pneumoperitoneum, duration to surgery > 24h and postoperative mortality were recorded. Patients were divided in 2 groups according to the presence or not of ascites. Results were as follows (* $p<0.05$):

| Ascites | Present (n=16) | Absent (n=6) |
|---|---|---|
| Acute abdominal pain | 3 (19%) | 5 (83%)* |
| Rebound tenderness | 3 (19%) | 5 (83%)* |
| Pneumoperitoneum | 15 (94%) | 4 (67%)* |
| Surgery > 24h | 6 (37%) | 0 * |
| Mortality | 10 (62%) | 1 (17%)* |

In patients without ascites symptoms of perforation were unremarkable. On the opposite in patients with ascites clinical presentation was non specific. Ascitic fluid culture demonstrated a bacterial peritonitis in 10 patients with numerous micro-organisms in 3. Pneumoperitoneum was present in all patients except in one who had previous abdominal surgery and in whom contrast medium radiography demonstrated a leak. Gastrectomy was performed in 3 patients without ascites and simple closure was performed in the remainings. Postoperative mortality was higher in patients with ascites. Five autopsied patients demonstrated recurrent ulcer and 3 had suture leakage.

These results suggest that: (a) in patients with ascites plain abdominal radiography should be first performed rather than numerous ascitic fluid analyses when a gastroduodenal ulcer is suspected; (b) a vagotomy should be associated to the suture of the perforation.

P0207 A NEW METHOD OF CHOLEDOCHOJEJUNOSTOMY
- An Animal Experimental Model -

Y.Ishizaki, T.Itoh, M.Takami, K.Shimada, Y.Nomura, K.Shimomura, S.A.Nayeem, Y.Izezuki

Second Department of Surgery,
Faculty of Medicine,
University of Tokyo, Tokyo, Japan

Biliary reconstruction for bile duct stenosis with severe inflammation has much difficulty due to severe adhesion around adjacent tissues and thinning of the bile duct wall. We devised a new method of biliary reconstruction using a stent tube which was positioned between the common bile duct and the jejunum.

Wistar rats with body weight of 250-350g were used. A stent tube which was fixed in the divided common bile duct was inserted into the jejunal lumen and settled by purse string suture. The common bile duct was only adjacent to the jejunum without direct anastomosis. The animals were sacrificed after one month (Group 1, n=10), two months (Group 2, n=10), and three months (Group 3, n=10). In Group 1, the stent tube had already disappeared in five cases and among them the anastomotic orifice could not be identified in three cases. In group 2 and 3, a stent tube was lost in all cases but the patency of the anastomosis was confirmed.

On microscopic examination, in Group 3, complete epithelialization was found in the site of anastomosis and inflammatory change was disappeared. In cases which had the patent anastomosis, intra-hepatic bile stasis was not recognized.

A period of more than one month was necessary for a satisfactory patency of the anastomosis. It took, however at least three months for a complete epithelialization between the common bile duct and the jejunum.

PO208 PYOGENIC LIVER ABSCESSES. THE ROLE OF INTERVENTIONAL RADIOLOGY

JR Polo, JL Garcia-Sabrido, F Camuñez, P Hernandez

J Calleja, M Lasala, A Quintans, L Gonzalez Bayon.

Hospital General "Gregorio Marañon". Madrid, Spain

Forty cases of pyogenic liver abscesses treated in the last ten years have been studied. local abscesses associated with acute colecistitis and infected hydatidic cyst have been excluded. All the patients without peritoneal rupture or associated biliary or portal pathology, have been treated with percutaneous drainage by means of pigtail catheters. The mean age was 55 years ( ranged of 16 to 85 years). There was 55 % male patients. The mean symptoms were fever ( 95 %), abdominal pain (65 %) and jaundice (35%). Fifteen percent of the patients have peritoneal signs due to rupture of the abscess into the peritoneal cavity. The mean with cell count was 17,000 ( range 6,000 to 34,000). The ethiology was : cryptogenetic, 50 % ; associated with cholangitis, 37 % ; associated with portal sepsis 7 % drug abuse, 5 %. Surgical treatment was used in 25 cases ( 62 %), specially in cases with biliary related pathology or intraperitoneal rupture. Fifteen of the cases (37 % ) were treated with interventional radiology. The overall mortality was 6 cases ( 15 %). The mortality in cases treated by surgery was 20 %. There was no mortality in cases treated with interventional radiology. We consider percutaneous drainage guided by computarized tomography the best way to treat pyogenic liver abscesses. Surgical treatment should only be carried out in cases with associated portal or biliary pathology or abscesses with peritoneal rupture

**P0209** "MINI-CHOLECSYTECTOMY": A SAFE SURGICAL PROCEDURE

F. Martínez-Ródenas, J.M. Badia,

J. Escalante, J. M. Vila, J. Pié, M. Solé

Dept of Surgery, Hospital Municipal da

Badalona, Badalona, Spain

Despite the introduction of new nonoperative techniques to treat cholelithiasis, cholecystectomy remains an excellent means to control the disease permanently. However, surgeons must improve their results in order to offer permanent cure with low morbidity and mortality and at a low cost.

"Mini-cholecystectomy" (MC) performed through a transverse right subcostal incision has evolved as a surgical procedure which offers a shorter and more comfortable postoperative period, and probably less wound-related and respiratory complications.

The main steps of the MC technique are shown. A comprehensive preoperative evaluation is mandatory to confirm the diagnosis and rule out any other concomitant abdominal condition. A right upper quadrant abdominal incision of 4 cm (+1cm) is enough to carry out the operation. The muscle rectus abdominis is either transected transversely or split longitudinally. The right upper quadrant of the abdomen is inspected. If technical problems arise or if the surgeon is uncomfortable, the incision can be easily enlarged. The incision offers a good exposure of the porta hepatis, allowing an operative cholangiogram to be performed or exploration of the common bile duct if required. Cholecystectomy can be accomplished either from the porta hepatis to the gallbladder fundus or the other way round. We used MC in 173 cases, with cholangiogram performed in 96% of patients. Morbidity was significantly lower than in cholecystectomies via other incisions (16.2% for MC, 26.1% for median laparotomy and 26.1% for right subcostal, $p < 0.05$).

COMMENTS: We suggest that massive HP during graft reperfusion that requires farmacologic treatment, must call the attention of medical staff of a possible primary graft failure.

**P0211**

### HYDATID DISEASE OF THE LIVER
### LONG TERM RESULTS OF SURGICAL TREATMENT

L. Cangiotti, S.M. Giulini, P. Muiesan, G. Tiberio
Department of Surgery - Brescia - ITALY -

Many surgical techniques have been used in the treatment of hydatid disease of the liver. The objective of the treatment should be aimed to minimize the high risks of complication and recurrence. We reviewed our records of 33 consecutive cases of hydatid disease of the liver treated surgically during a 6 year period (1974-1979) to determine the complications and the long term results ( 10 years) of different surgical procedures applied. Partial pericystectomy and tube drainage was the most common treatment (30/49 cysts) followed by total pericystectomy (15/49) and partial liver resection (4 cases). Complication rates were 10 %, 7 % and 0 % respectively. There has been no perioperative mortality.

In 1989 we asked all patients to investigate cystic recurrence by CT scan or abdominal ultrasound: we were able to study 23 patients. Only three of them showed an asymptomatic recurrent disease. They all had undergone partial pericystectomy in 1974 (2 cases) and 1977 for recurrent hydatid disease of the right hepatic lobe and, in one case, even of the rectus muscle.

The complication and recurrence rates seem to be higher in conservative surgical procedures than in more radical treatment, but indiscriminate application of total pericystectomy or major liver resection would lead to a high incidence of major postoperat ive complications and also mortality.

In fact we performed total pericystectomy or wedge resection only for peripheral cysts and believe that liver resection for hydatid cyst is a very radical approach for this kind of disease.

### P0212 A SCANNING AND TRANSMISSION ELECTRON MICROSCOPIC STUDY OF SMALL BOWEL IN EXPERIMENTAL CHOLESTASIS IN THE RAT

S. Singh, M.E. Bailey

University of Surrey and The Royal Surrey County Hospital, Guildford, Surrey, England

The post-operative complications of obstructive jaundice have been attributed to Endotoxaemia, a probable result of reduced intraluminal bile salt levels causing in an increased absorption of endotoxin with subsequent spillover into the systemic circulation (Bradfield 1974, Bailey 1976, Pain *et al* 1985, Pain *et al* 1987, Cahill and Pain 1988).

12 Wistar rats underwent ligation and division of the bile duct, developing obstructive jaundice. They were sacrificed 3 weeks later and the duodenum, mid small bowel and terminal ileum were studied using both scanning and transmission electron microscopy.

Scanning Electron Microscopy demonstrated the following changes : a) irregularity of microvillus architecture with increased segmentation, b) destruction of the microvillus tip, c) increased adhesion of debris.

Transmission Electron Microscopy showed alteration of the microvillus architecture with remarkable prominance of the rootlet with lateral extensions joining adjacent rootlets. Some cells showed increasing mitochondrial degeneration.

The changes were most pronounced in the Mid-Small bowel with only minimal changes in the terminal ileum, the site of bile salt reabsorption.

These changes may be responsible for the observed increase in endotoxin absorption into the portal circulation, and may contribute to the clinical problems seen in patients with obstructive jaundice.

**References**

Bradfield JWB Lancet 1974; ii: 883-886.

Bailey ME BJS 1976;63:774-778.

Pain JA, Cahill CJ, Bailey ME BJS 1985; 72: 942-945.

Pain JA, Bailey ME Eur. Surg. Res. 1987; 19: 207-216.

Cahill CJ, Pain JA Surg Annu 1988; 20: 17-37.

### P0213 ADENOCARCINOMA IN CAROLI'S DISEASE TREATED BY LIVER TRANSPLANTATION

J.BALSELLS, JL.LAZARO, E.MURIO,R.CHARCO
I.DIAZ, J. BONNIN, C.MARGARIT
HOSPITAL G.VALL D'HEBRON.BARCELONA.SPAIN

Caroli's disease, a congenital dilatation of intrahepatic bile ducts, presents frequent complications as intracystic lithiasis, cholangitis, jaundice, fever, liver abscess and eventually liver cirrhosis. In a 5 to 7% of the cases a malignant tumor, as cholangiocarcinoma or hepatoma develops complicating the course of the disease. Liver transplantation in Caroli's disease can be indicated in uncontrolled cholangitis, end-stage liver cirrhosis or when malignancy appears.
A 25 years old female had been diagnosed of Caroli's disease at the age of 11 years. Since then, several episodes of fever and abdominal pain resolved rapidly with antibiotic therapy. In 1989 she was explored again after presenting another episode of cholangitis. The abdominal ultrasound and CT scan showed enormous dilatation of the intrahepatic bile ducts particularlyin the right lobe with extense intracystic lithiasis and a solid mass occupying part of the cysts. FNA cytology showed a papilar adenocarcinoma. At exploratory a huge tumor was found occupying part of the right and left lobes. Intraoperative US showed another two nodules in the left lobe. The tumor was considered unresectable by partial hepatectomy,examination of hilar lymph nodes was negative. Two weeks later, the patient underwent an orthotopic liver transplantation. A choledocojejunostomy with a Roux-en-Y loop of jejunum was used for biliary reconstruction as the recipient common bile duct was also dilated. The hepathology report of the liver confirmed Caroli's disease with adenocarcinoma arising in the intrahepatic bile ducts. She was discharged three weeks after OLT and she is alive and well with normal liver function and without evidence of disease 5 months after OLT.
To our knowledge this is the first case reported of adenocarcinoma in Caroli's disease treated by liver transplantation.

### PO214 DEVELOPMENT OF COLLATERAL CIRCULATION IN HETEROTOPIC AUXILIARY LIVER TRANSPLANTATION

L.LORENTE, J.ARIAS, J.I.TROBO, M.A. ALLER, I.ISPIZUA, H.DURAN Hospital San Carlos, Madrid, SPAIN.

One of the causes of auxiliary liver transplantation failure is the inter-liver competition between the host liver and the graft for the hepatotrophic factors of the portal blood.

We have developed an experimental model of heterotopic partial (30%) liver isotransplant using Wistar rats so as to study this competition.

Splenoportography and dissection showed the existence of collateral circulation. The collaterals at 90 days post-transplant consisted of veins from the portal vein to the host liver, paraesophageal veins and splenorenal veins. At 60 days post-transplant there were portal vein to the host liver and splenorenal veins, and at 30 days post-transplant there were only veins from the portal vein to the host liver. Graft athropy at 90 days was associated with a severe degree of bile duct proliferation.

The gradual development of portal hypertension causes porto-systemic collateral circulation and the graft losses the portal hepatothrophic factors. The late development of the portal hypertension and the the biliary proliferation could be caused by the hepatic arterial ischemia in this experimental model. In the auxiliary liver transplantation in rats the functional competition is stablished through the development of portal hypertension in the graft liver and this one is intimately relates to the existence of biliary pathology in the grafts portal tracts.

**PO215** EXTRACORPOREAL SHOCK WAVE LITHOTRIPSY OF GALLBLADDER STONES: IS THE MEDICAL ADJUVANT THERAPY MANDATORY ?

M. Kracht, F. Lacaine and the French Associations for Surgical Research and Medical Evaluation (A.R.C. and A.C.A.P.E.M.), Colombes and Paris, France

The aim of this prospective multicenter study was to determine the effectiveness of lithotripsy and the role of medical adjuvant therapy in gallbladder stone elimination. Inclusion criteria were: symptomatic gallstone disease, functionnal gallbladder, 1 to 5 stones with a diametre up to 30 mm be they radiolucent or not. Patients were treated under simple analgesia on a Sonolith 3000. Up to 4 sessions were performed if fragmentation was incomplete. Fragmentation was evaluated by ultrasound at 1,3,6 and 12 months and annually thereafter. One month after the last session patients were entered into a randomized study according to the residual fragment size: for stones with less than 2 mm diametre, adjuvant dissolution therapy versus no therapy. For all patients with stones between 2 and 5 mm, ursodesoxycholic acid was given, whereas all patients with fragments greater than 5 mm, cholecystectomy was performed. 200 patients have so far been treated. 71 % of stones were radiolucent, and there were 61 % of single stones.
Two patients required endoscopic sphincterotomy to clear choledochal fragments. Transitory hematuria (4 %) and biliary colic (2 %) were observed.Fragmentation of stones were observed in 80 % of cases, and was more frequent in radiolucent (82 %) than in calcified (75 %) stones (NS). Fragmentation was more frequent in solitary (87 %) than in multiple (71 %) stones ($p < 0.05$). In 30 % of patients with fragmentation, fragments were less than 2 mm in diametre. Eventually, 30 % of patients underwent cholecystectomy. We conclude that surgery was required in 30 % of patients, that an adjuvant medical therapy is indicated in 45 % of patients and that only 25 % of the patients may not require further therapy.

### PO216 DIAGNOSIS OF PANCREATIC CANCER USING DUPAN-2 MONOCLONAL ANTIBODY.

E.Opocher, G.P.Spina, R.Santambrogio, G.Cucchiaro, L.Vignati, G.L.Tarolo, F.Marelli, F.Borzio, P.Pederzoli*.

San Paolo Insitute of Biomedical Science - University of Milan

*Surgical Clinic - University of Verona, Italy

Cancer of the pancreas is a diagnostic and therapeutic challenge. Diagnostic methods that could identify serum-borne tumor markers would be the most useful for early diagnosis. We have previously reported in a prospective study that CA19-9 levels were elevated in virtually all patients with pancreatic carcinoma, but abnormal levels were also detected in patients with mechanical biliary obstruction. Therefore, we performed a preliminary study to evaluate the potential usefulness of DUPAN2 compared with CA19-9 and CEA in the diagnosis of pancreatic tumor. Eighty-nine patients were included: 30 normal volunteers (NV), 30 patients with pancreatic tumor (PT) and 29 with chronic pancreatitis (CP). Dosage of DUPAN2 antigen levels was performed by a sandwich enzyme immunoassay kit (Kyowa Medex Co.,Ltd) using the DUPAN2 monoclonal antibody. Mean serum levels were: 21.9±41.9 U/ml (95%C.I. from 6,8 to 37,2) in NV group, 1521.3±2212.5 U/ml (95%C.I. from 717.4 to 2325.1) in PT group and 498.3±994.1 U/ml (95%C.I. from 130.9 to 865.6) in CP group. Though the analysis of variance showed a significant difference between the 3 groups (p=0.0003), the wide dispersion of values in PT and CP groups, as shown by the confidence intervals, does not seem to guarantee a clinical value to this statistical difference. Therefore, the DUPAN2 serum antigen levels were not helpful in this work in distinguishing patients with pancreatic tumor from those with chronic pancreatitis. This finding was also confirmed by the mild specificity of test (61%), in spite of a high sensibility (83%). Finally DUPAN2 showed no correlation (Pearson's test) with CA19-9 (r=0.2331) and CEA (r=0.1130) in PT goup.

In conclusion, this preliminary study showed that DUPAN2 serum assay is a highly sensitive test; but it does not improve the results obtained with CA19-9. It is necessary to recruit further patients to confirm these data and to recommend the routinary use of DUPAN2 serum assay in the diagnosis of pancreatic cancer.

This work was supported by grants from C.N.R.-Progetto Finalizzato Oncologia.

### PO217 MANAGEMENT OF PRIMARY HEPATOLITHIASIS

Kunihisa Miyazaki*, Masato Furukawa*, Toshiomi Kusano*, Tsukasa Tsunoda**, Ryoichi Tsuchiya**
* Dept of Surg, Nagasaki Chuo National Hosp
** 2nd Dept of Surg, Nagasaki Univ Sch of Med, Nagasaki, Japan

Primary hepatolithiasis(PHL) is the most refractory and complicated condition among the various benign biliary tract diseases. There are still controversies on therapeutic choice for PHL.

We classified PHL into five types(Type I-V), on the basis of morphological findings obtained by computed tomography, in order to elucidate how to follow and treat patients with PHL. The classification depends mainly on the presence of atrophy of hepatic parenchyma (AHP) and dilatation of intrahehatic bile ducts(DIHBD)(Table 1).

Table 1. Classification of PHL

| | AHP | DIHBD | Total |
|---|---|---|---|
| I | (-) | (-) | 53 |
| II | (-) | (+) | 11 |
| III | (+) | (-) | 8 |
| IV | (+) | (+) | 19 |
| V | (++) | (++) | 21 |
| Total | | | 112 |

Methods of treatment were selected for each patient according to the classification. In Type I, most of these patients were asymptomatic and they were not treated but just followed, since nither AHP nor DIHBD was observed. In Type II, AHP was not found, so that improvement of liver function could be expected by removal of stones and bile stasis. Stones in the intrahepatic bile ducts were completely removed by surgical and/or endoscopic procedures. In Type III, IV and V, hepatic resection was performed to remove all of stones together with atorophied hepatic lobes or segments since hepatic function of the affected area could not be expected to improve.

The patients were followed-up five years or less postoperatively. Recurrence was not observed. All patients had faborable postoperative courses and resumed ordinary lives.

It was suggested, therefore, that the present classification of PHL was helpfull in determining therapeutic strategy for PHL.

### P0218 ESTIMATION OF ORAL GLUCOSE TOLERANCE TEST AS A PROGNOSTIC ASSESSMENT FOR HEPATECTOMY

H. Tsuge, H. Mimura, K. Orita
Okayama University Medical School, Japan

Approximately 80% of patients bearing hepatocellular carcinoma in our clinic have liver cirrhosis, which limits the resectability of their hepatic malignancies. An accurate preoperative assessment of the hepatic functional reserve has been very important to prevent posthepatectomy liver failure. In patients with liver cirrhosis, a high frequency of abnormal carbohydrate tolerance, "hepatogeneous diabetes", has been demonstrated, and oral glucose tolerance test (OGTT) is considered to be useful as an index of the preoperative assessment of the hepatic functional reserve.
To estimate quantitatively OGTTs with 50-gm. and 75-gm. loads in 158 patients, T-max: time with the highest value of blood glucose, and C-max: the highest value of blood glucose were decided and used. Divergent doses of oral glucose load did not influence the mean values of T-max and C-max in patients with normal liver. On the contrary, in patients with liver cirrhosis, a significant increase of T-max value was present only after 75-gm. of glucose. There were many significant correlations between the results of liver function tests and T-max and C-max of 50-gm. OGTT, respectively, but not of 75-gm. OGTT. However, the relationships between occurrences of posthepatectomy liver failure and values of T-max and C-max of 50-gm OGTT were not found. We concluded that the degree of the glucose intolerance paralleled that of the hepatic functional impairment, however, OGTT was not useful as an indicator for prediction of posthepatectomy liver failure.

**P0219** STUDIES ON EPIDERMAL GROWTH FACTOR RECEPTOR IN CARCINOGENESIS IN RAT LIVER.

H.Minote, K.Inoue, C.Mori, S.Higashide, K.Hoshino, T.Tobe.
First Department of Surgery and Third Department of Anatomy, Faculty of Medicine, Kyoto University, Sakyo-ku, Kyoto, JAPAN.

Immunohistochemical study was undertaken using anti-epidermal growth factor (EGF) receptor antibody in normal liver and 3'-methyl-4-dimethylamino-azobenzen (3'-Me-DAB) induced cholangiocarcinoma in rats. Eleven experimental 5-week-old male Wistar rats were provided with a diet containing 0.06% 3'-Me-DAB, and 5 controls with oriental yeast chow ad libitum. Liver tissues obtained from experimental and control rats at the intervals of 8, 10, 12, 14 and 16 weeks after the commencement of treatments were fixed in 4% paraformaldehyde, dehydrated, and paraffin embedded. Paraffin sections were examined histologically after hematoxylin and eosin staining or reacted immunohistochemically with the anti-EGF receptor rabbit antibody produced against synthetic peptides corresponding to the amino acid residues 1173-1186 in human EGF receptor. This anti-EGF receptor antibody has already been purified with affinity chromatography (Akiyama 1986). Multiple nodules of cholangiocarcinoma were observed on the liver tissues by hematoxylin and eosin staining from 8 to 16 weeks after administration of 3'-Me-DAB. The immunoreactive products indicating the site of the EGF receptor were stained both on the cytoplasm of hepatocytes in control rats and on the cytoplasm of cholangiocarcinoma cells in rats treated with 3'-Me-DAB, showing a similar extent of staining. On the other hand, EGF receptor immunoreactivity was observed on the cytoplasm of regenerating hepatocytes in non-cancerous tissues adjacent to nodules of cholangiocarcinoma much more clearly and strongly than that was observed in control liver tissues and in cholangiocarcinoma cells. These interesting phenomena may indicate that the appearance of EGF receptor is associated with the regenerating process and carcinogenesis in rat liver.

Reference:Akiyama T. Arch.Biochem.Biophys. 1986;245:531.

### PO220 RESULTS OF HEPATIC TRANSPLANTATION IN PATIENTS OVER 50 YEARS OF AGE

RM Merion, DA Campbell, JM Ham, MR Lucey, KS Henley, JG Turcotte.
University of Michigan Medical School, Ann Arbor, Michigan, USA.

Liver transplantation has become standard treatment for a variety of hepatic disorders, but this procedure has not been regularly offered to older patients. An age limit of 50 years is still observed in many centers. We reviewed our experience with 33 liver transplant recipients over the age of 50 years (range 51-71; mean 58±1) and compared the results to 92 younger adult recipients (range 19-49; mean 39±1) transplanted during the same interval (8/85-9/89; mean follow-up 1.3 yr). There were no significant differences between the two groups with regard to donor factors (age, sex, length of hospital stay, cause of brain death, or vasopressor use), preservation solution, ischemic time, preoperative serum creatinine, cytomegalovirus status, or immunosuppressive protocol. Older recipients underwent cardiopulmonary evaluation with echocardiography and/or dipyridamole thallium scan prior to acceptance. There were no significant differences in post-operative recovery of liver function in the two groups (ALT, AST, bilirubin, prothrombin time; bile output). ICU stay was similar in the two groups (10±2 v 15±2 days; P=NS). Older patients required a longer period of mechanical ventilation (6±3 v 4±1 days), but this was not statistically significant. The incidence of treated allograft rejection during the first post-transplant year was 58% in the older group and 51% in the younger group (P=NS). The mean number of rejection episodes per patient was not significantly different (0.7±0.2 v 1.0±0.1 episodes; P=NS). Steroid-resistant rejection episodes requiring OKT-3 tended to occur more often in older than younger recipients (26% vs 18%), but this difference was not statistically significant. Duration of hospitalization was not significantly different (37±5 v 37±3 days; P=NS). There were no significant differences in the doses of corticosteroids during the first month. Doses of azathioprine and cyclosporine, as well as cyclosporine levels, at 30 days, 6 months and 1 year were compared, and no significant differences were found. Serum creatinine and blood pressure were assessed 30 days, 6 months and 1 year post-transplant. There were no significant differences between the two groups with the exception of a higher diastolic blood pressure at 1 year among younger recipients (91±2 v 82±3 mmHg; P=0.009). One year actuarial patient survival was 78% for older recipients and 71% for younger patients (P=NS; log-rank test). The number of readmissions to the hospital in the first year was not significantly different and the overall level of rehabilitation has been excellent in surviving patients. In conclusion, patients over 50 years of age with end stage liver disease should no longer be categorically excluded from liver transplantation programs. Long term survival and excellent rehabilitation can be expected in the majority of carefully selected patients.

### P0221 ANTITUMOUR ACTIVITIES OF SPLEEN-DERIVED LAK FROM PATIENTS WITH HEPATOCELLULAR CARCINOMA

**A.Kawata, Y. Une, J. Uchino**
**Hokkaido Univ., Sapporo, JPN**

Hepatocellular carcinoma (HCC) is a highly malignant tumor which has a high incidence of recurrence after resection. Recent advancement in the study of cytokines has introduced the clinical application of autologous cytotoxic cells from cancer patients. We have studied the antitumor activities of LAK cells from spleens (spleen-LAK) of patients with HCC.

Sixteen cases of HCC were examined in this study. We harvested enough mononuclear cells for the preparation of LAK cells from the spleens of patients with HCC. The total recovery was $1.75 \times 10^8 \pm 0.87$ (mean $\pm$ SD) per gram. The induction of spleen-LAK cells was carried out by culture in fresh medium containg 1,000 JU/ml of IL-2. The cytotoxicity of the spleen-LAK cells maintained high levels during the culture period ranging 3 to 30 days. Subsets of spleen-LAK cells were identified as activated T lymphocytes (DC8+, CD25+, HLA-DR+).

Three cases of unresectable HCC and 8 cases of resected HCC were treated with an intra-arterial injection of $1.0 \times 10^9$ to $1.6 \times 10^{10}$ spleen-LAK cells, which was administered 2 days after the intra-arterial infusion of adriamycin. Two of the three cases with unresectable HCC responded. All of 8 cases had no recurrence for 3 to 8 months after the resection.

In conclusion, spleen-derived LAK cells may be appropriate effector cells and show promise as an effective means of adoptive immunotherapy in hepatocellular carcinoma patients.

### P0222 ACUTE PANCREATITIS AND NORMOAMYLASAEMIA: A COMMON COMBINATION

M.Winslet, C.Hall, NJM.London*, JP.Neoptolemos
Department of Surgery, Dudley Road Hospital and Leicester Royal Infirmary*

Hyperamylasaemia has been reported to be an unnecessary prerequisite for the diagnosis of acute pancreatitis (AP), particularly in patients with an alcoholic aetiology where up to 58% may have normoamylasaemia [1]. To determine whether this finding is real or due to delayed presentation, serial amylase levels in 409 patients with AP (gallstones=296, alcohol=113) presenting within 24 hr of symptom onset, were assessed prospectively. Serum amylase was measured by the Phadebas method (normal range 70-300 IU/L) diagnostic level for AP=>1000 IU/L. AP with non-diagnostic amylase levels was confirmed by urinary amylase, serum lipase and CT scanning.

| | Admission | 24 hr | 48 hr | 72 hr | 1 wk |
|---|---|---|---|---|---|
| Biliary pancreatitis (n=296) | | | | | |
| Amylase >1000 (%) | 97.3 | 80.0* | 44.4 | 7.0 | 7.1 |
| > 300 (%) | 99.3 | 96.7 | 80.1 | 48.8 | 29.7 |
| Alcoholic pancreatitis (n=113) | | | | | |
| Amylase >1000 (%) | 84.0 | 40.6* | 35.0 | 8.1 | 13.0 |
| > 300 (%) | 99.1 | 96.9 | 78.3 | 43.2 | 32.6 |

*p<0.05 (Chi-squared)

Classified by modified Ranson score, there was no difference in the incidence of hyperamylasaemia or diagnostic amylase levels, apart from alcoholic pancreatitis at 24 (mild=28%, severe=85.7%, p<0.05) and 48 (mild=35.7%, severe 85.7%, p<0.05) hours.

In the majority of confirmed cases of AP the initial amylase level will be diagnostic. Normoamylasaemia merely reflects delayed presentation rather than failure of pancreatic production. In AP, particularly of alcoholic origin, the serum amylase should always be interpreted in relation to symptom duration and equivocal cases confirmed by a combination of urinary amylase, serum lipase and CT scanning.

[1] Clavien P-A, Robert J, Meyer P, et al. Acute pancreatitis and normoamylasemia. Ann Surg 1989; 210:614-20.

### P0223 EFFECT OF BILE ON PERITONEAL PHAGOCYTES IN E.COLI PERITONITIS

R. Andersson, C. Schalén, U. Srinivas, K.-G. Tranberg. Departments of Surgery, Medical Microbiology, Clinical Chemistry, Lund University, Sweden.

Experimental work from our group has previously demonstrated that the addition of intraperitoneal (i.p.) bile in E.coli peritonitis in the rat increases mortality, lowers the number of bacteria associated with each peritoneal phagocyte (as determined after staining with non-specific esterase and Giemsa) and increases the number of E.coli within the peritoneal cavity and, subsequently, in blood. Thus, it appears that local host defense is affected in some way by the i.p. presence of bile in E.coli peritonitis. The aim of the present study was to determine the effect of bile on the peritoneal cellular effluent and on superoxide release, bacterial uptake and bacterial killing by peritoneal phagocytes in experimental E.coli peritonitis in the rat.

The following characteristics of peritoneal phagocytes were determined at 1, 4 and 10 h after i.p. administration of E.coli (E.coli peritonitis) or E.coli + bile (E.coli + bile peritonitis): number, cellular composition (monocytic cells, polymorphonuclear leukocytes), superoxide release (at 10 h only), bacterial uptake and killing of bacteria as determined by acridine orange technique.

After induction of peritonitis, the number of peritoneal leukocytes increased with time whereas the number of monocytic cells in the peritoneal fluid showed a tendency to decrease. The extent and pattern of change of the peritoneal cellular composition was similar in the two peritonitis groups. Monocytic cells and polymorphonuclear leukocytes behave similarly with respect to the functional parameters studied. Phagocytic activity, as measured by the numbers of bacteria associated with each cell and by superoxide release, was decreased in E.coli + bile peritonitis as compared to E.coli peritonitis. In contrast, the capacity of phagocytes to kill bacteria did not differ between the two types of peritonitis.

In conclusion, i.p. bile in experimental E.coli peritonitis impaired uptake of bacteria and superoxide release by peritoneal phagocytes, but did not affect the number, composition or killing capacity of these cells.

### P0224 BILIARY TRACT TUMORS-ANALYSIS OF 62 CASES.

F. Castro Sousa, P.Simões Carvalho, C.Mesquita, L.Silveira, C.Loureiro.
Coimbra University Hospital, 3th Surgical Department, PORTUGAL.

Biliary tract tumors (BTT) are diagnosed with increasing frequency; therapeutic options continue to be the theme of controversy.

Between 1980-1989, 62 patients (29 men, 33 women) with hystologic or cytologic comproved BTT were surgically treated in our department: 36 common duct tumors, 12 ampulomas and 14 gallbladder tumors. Obstructive jaundice was the revealing symptom in the great majority of the patients and ERCP and CPT were of particular value in preoperative diagnosis. Three patients received emergency operations and seven had been previously operated unsuccessfully. Total resecability was 34% and was maximus (73%) for ampulomas. Several paliative procedures were used and namely 12 intrahepatic cholangiojejunostomies (segment III) and seven entubations. Operative mortality was 8% (2 month) and complications (namely infections) appeared in five patients. No preoperative decompression of the biliary tract was undertaken. Actuarial survival was particulary poor in gallbladder tumors.

The results of this study seem to show that a curative resection is rarely possible for treatment of biliary tract tumors. Nevertheless a criterious selection of paliative techniques is able to improve the quality of life and, sometimes, even the extension of survival of these patients.

PO225

### A NEW ULTRASONOGRAPHIC FEATURE OF ACUTE CHOLANGITIS

N.I. Markham[1], P. Gaines[2], A.K.C. Li[1] and C. Metreweli[2]
Departments of Surgery[1] and Radiology[2], Prince of Wales Hospital, Chinese Unviersity of Hong Kong, Hong Kong

We describe thickening of the wall of the common bile duct (CBD) on ultrasonography in patients with cholangitis, a feature only previously described in patients with AIDS[1]

6 patients, all with clinically severe acute cholangitis and underlying recurrent pyogenic cholangitis (RPC) were investigated with ultrasound and found to have a thickened dilated CBD with a "double-line" appearance of the wall. All had lower CBD stones and both extra-hepatic and intra-hepatic duct dilatation. The "double-line" is seen as a bright mucosal and muscular layer, separated from another bright serosal line by a thick, dark zone of anechogenicity. This echo-poor area probably represents perimuscular oedema and cellular infiltrate, similar to the changes described in the gall bladder wall in acute cholecystitis[2].

We feel this new "double-line" sign appears to be a reliable indicator of acute cholangitis in patients with RPC and therefore is of value in the assessment of this difficult group of patients.

References:

1. Defalque D.; Menu Y. et al. Sonographic Diagnosis of Cholangitis in AIDS Patients. Gastrointest. Radiol. 1989 14 143-147.

2. Raghavendra B.N.; Feiner H.D. et al. Acute Cholecystitis: Sonographic-Pathologic Analysis AJR 1981. 137, 327-32.

### P0226 OUR EXPERIENCE IN GALLSTONE DISSOLUTION

P.Miševska-Mukaetova, P.Davčev

Clinic of gastroenterohepatology,
Skopje, Yugoslavia

For the last few years the medical dissolution of the cholesterol gallstones appears as an alternative of the surgery treatment. Chenodeoxycholic acid (CDCA) is a well-known agent for the dissolution; also ursodeoxycholic acid (UDCA), its $\beta$-hydroxy epimer, showed similar properties.

Our clinical experience is based on 72 treated patients. From 72, only 26 (36,11%) tolerated the therapy completely, from which we got the following: completely dissolution gallstones 10 (38,46%), partial dissoluted gallstones 2 (7,69%) or total 12 (46,15%).

Interruption of the therapy was due to: elevated transaminases 1 (3,84%), elevated amylasis 1 (3,84%) and surgery treatment 6 (23,71%). The unsuccessfull therapy by 6 patients is due to more gallstones (2), middle size (3), inorganic shell (1). The treatment was with CDCA by 4 and CDCA+UDCA by 2.

The successfull therapy with 10 patients is a consequence of more small gallstones (6), one middle size gallstone (1), two middle size gallstone (1) and three middle size gallstones (1). The treatment was with CDCA by 7, with UDCA by 1 and CDCA+UDCA by 2. The duration of the therapy is 4-12 months (average 8 months). The control was clinically, sonografically and radiologycally.

The medical dissolution of cholesterol gallstones is an alternative of the surgery treatment in case of good indication. The combined therapy CDCA+UDCA is with the smaller number of side effects and more successfull. Our modest experience is a contribution to this conception.

**PO227** LOCAL LAVAGE IN NECROTIZING PANCREATITIS AND BRADYKININ RECEPTORS

T.Mavromatis,T.Pavlis,G.Baltopoulos,
K.Daskalakis,D.H.Kintzonidis.

"Evangelismos" Hospital, Athens
Greece

In many patients with severe acute pancreatitis a rapid and dramatic clinical improvement accompanied by relief of pain follows the peritoneal dialysis. The mechanism of action seems to be a washing out of the peritoneal cavity with concomitant removal various toxic and vasoactive substances such kinins. The same result is succeeded with the postoperative local lavage in necrotizing pancreatitis (NP) plus continuous evacuation of devitalized tissue and bacteria.
It is known that the administration of Fresh frozen plasma (F.F.P.) in a patient activates the kinin system through the containing Factor XII with production of bradykinin.
In 5 patients age 52-68 years old who had biliary NP 440ml of F.F.P. were administered i.v. before and during local lavage in the peripancreatic area. In all these patients the usual invasive haemodynamic monitoring including use of Swan-Ganz catheter were used.
Significant differences in haemodynamic changes during the administration of F.F.P. were found.
It is suggested that local lavage in NP acts by removing bradykinin from the peripancreatic area and this results in raising the blockade of bradykinin receptors on peripheral blood vessels possibly due to reduced amounts of circulating bradykinin.

### PO228 OBVIOUS ENDOSCOPIC ABDOMINAL PERFORATIONS SUCCESSFULLY TREATED BY CONSERVATIVE WAY

M.Gergely[+], L.Sulyok[++], Z.Seress[++], Cs. Csonka[+], 1st Dept.Surg.[+] & 2nd Medical Dept.[++] County Hosp. Szolnok, Hungary.

The open perforations of the gastrointestinal tract in the course of an endoscopic manipulation usually results a clear and urgent surgical intervention.
We present here three cases - two endoscopic papillotomy, and one large bowel polypectomy - where the obvious and transitional free perforation of the GI tract was clearly proved by the X-ray examination,and the meticulous clinical - surgical observation didn't lead to a surgical intervention, which was finally perfectly unnecessary in all of these three cases.
At one of our patients after endoscopic polypectomy /on the large bowel/ developed an abdominal pain, and the native X-ray examination showed air under the diaphragma. In one of two papillotomies the situation was similar, and in the case of third patient after papillotomy in the course of stone removal, the Dormia - basket was seen free in the abdominal cavity.
None of them showed any signs of acute abdomen at the time of surgical consultation, which was carried out shortly after the detection of the perforation. All of these patients were obese, high risk and aged ones, and healed spontaneously,without operation.
We do not state ,of course, that in the cases of so called acute abdomen the surgery should be abandoned, or even delayed, but in cases of the lack of typical signs of acute abdomen, the presence of air under the diaphragma itself cannot be the basic and only indication of a surgical exploration.
In similar perforations , with a good circulation, perfect general state and without any signs of acute abdomen, the surgical intervention can be delayed and in some cases once for all abandoned.

Reference:

L.Sulyok, E.Szücs; /Lecture, Hung./ 26th Congress of Hung.Gastroenterol.Soc., Balatonaliga,16-19 May 1984.

**PO229** *THE MIRIZZI SYNDROME*

*A New Classification and Surgical Alternative*

*A.Menteş,H.Çavuşoğlu,A.Çoker,O.Özbal*

*Dept. of Surg.,Agean University Hospital,*

*İzmir,Turkey*

*The Mirizzi Syndrome is described as external obstruction of the common hepatic duct due to an impacted stone within the cystic duct or the gallbladder itself.This syndrome is an uncommon cause of extrahepatic obstructive jaundice and the body of the published literature is built on case reports.Here,we report a series of 16 patients with this syndrome and propose a new classification based on surgical findings.*

*Diagnostic workup included ultrasonography,percutane transhepatic cholangiography and ERCP however,no one method proved to be useful alone.Surgery was performed in 14 patients and confirmed diagnosis in 6 patients and established correct diagnosis in 8 patients. Surgery is also the most available method in determining the severity of the condition.As a result,patients with Mirizzi's Syndrome fall into one of three types:*

*Type I: Impacted gallstone in the cystic duct or neck of the gallbladder.Contigous inflammation of extrahepatic biliary structures present.There may be external compression to the common hepatic duct however,all anatomic structures are intact.*

*Type II: Fistulisation between the gallbladder and common hepatic duct.Gallstone is in the gallbladder.Jaundice is mainly due to external compression.*

*Type III: Complete integration between gallbladder and common bile duct.Gallstone is in the extrahepatic bile duct.*

*Simple cholecystectomy with or without T tube drainage of the common bile duct is indicated in patients with Type I disease while, cholecystectomy,choledochotomy and T tube drainage is the procedure of choice in Type II patients.However,most patients in our series (62.5 %) had Type III disease,with significant loss of bile duct wall.Partial cholecystectomy and repair over a T tube stent may be apreferable method in the surgical treatment of patients with Type III Mirizzi Syndrome.*

### P0230 THE EVALUATION OF THORACIC DUCT LYMPH DECOMPRESSION IN EXPERIMENTALLY PRODUCED ASCITES IN DOGS

F. Lukič, V. Simčič, M. Snoj

The Institute of Oncology, and Biotechnical Faculty,University of Ljubljana, Yugoslavia

The diameter of the supradiaphragmal part of the vena cava was reduced by polyethylene wrapping for about 75% through right thoracotomy in endotracheal anesthesia. The animals developed ascites 3-4 weeks following operation. The ascites persisted for 4-6 months, and disappeared spontaneously later on. The animals were normal, except for their swollen abdomens. The protein level of such ascitic fluid varied from 0.3 to 4.7 %. At the time of operative treatment the pressure in the place of vena cava stenosis was 15.5 - 20 cm of water (average 17.8 cm of water) and above the stenosis 4 - 5.5 cm of water (average 4.5 cm of water). The pressure of the thoracic duct lymph three weeks after operation was 20 - 40 cm of water (average 31 cm of water), and the pressure in the vena cava was 17 - 20 cm of water (average 17.8 cm of water). After 8 weeks the pressure in the thoracic duct was 2 - 10 cm of water (average 5.9 cm of water) and in the vena cava 21 - 25 cm of water (average 22 cm of water).

Ascites depletion by punction normalized the pressure in the vena cava and thoracic duct in a few hours. Three weeks after the appearance of ascites, thoracic duct to jugular vein anastomosis was performed for decompression of the thoracic duct lymph flow. Ascites disappeared 3-4 weeks later. The thoracic duct lymph flow represents a by-pass for portal hypertension in the first 8-10 weeks; later on the pressure in the thoracic duct becomes normal, which renders the by-pass questionable.

P0231 SURVIVAL AFTER HEPATIC RESECTION FOR HEPATOCELLULAR CARCINOMA

T. Tsuzuki, M. Ueda, A. Sugioka,
T. Kanai
Keio University, Tokyo, JAPAN

Between July 1973 to November 1989, 352 patients were admitted and 145 underwent resection. One hundred patients (68.9%) had cirrhosis and 4 had obstructive jaundice. Resection of 2 or more segments were carried out in 30 of 100 patients with cirrhosis and 30 of 45 patients without cirrhosis. Nineteen patients died mostly of liver failure.

One hundred and twenty six patients were discharged from the hospital. The 5-year actuarial survival rate calculated by the Kaplan-Meier method was 40% and 17 patients lived more than 5 years. Six of the 17 patients underwent resection of 2 or more segments and 5 of the 6 were cirrhotic patients. Patients with tumors less than 5 cm in diameter lived longer than those with larger tumors. Patients with tumor thrombi in the portal vein had shorter survival than those without tumor thrombi. No significant differences in survival were observed between patients with cirrhosis and chronic hepatitis. Edmondson's grading was not correlated with survival. $HB_sAg$-positive patients proved to be 5-year survivor but tended to succumb to late recurrence or new growth within 10 years.

Four patients with obstructive jaundice tolerated extensive resection and 1 patient is alive 6 years and 9 months.

Seven patients with tumor thrombi in the portal trunk or inferior vena cava and right atrium underwent hepatic resection with removal of tumor thrombi. The longest survivor lived 2 years and 2 months after extended right lobectomy with removal of tumor thrombus in portal trunk.

The prognosis for patients with hepatocellular carcinoma is not necessarily unfavorable as formerly thought.

### PO232 HYDATID CYST LIVER- COMPLICATIONS AND MANAGEMENT

JD Wig, NM Gupta, SM Bose,

RN Katariya, SK Khanna

Department of Surgery,

P.G.I.M.E.R., Chandigarh, India.

Hydatid disease is a major World health problem and symptoms arise from local pressure, leakage, infection or rupture into the biliary tree. The present study reports our experience with the complications of hepatic hydatid cyst and their management.

27 patients with complicated hydatid cysts were managed at the Institute hospital over the last 15 years. Various complications encountered were: intrabiliary rupture (n=8), Extraperitoneal (n=2), intraperitoneal (n=3), hepatobronchial fistula (n=2), small bowel (n=1), suppuration (n=9), pressure effects - inferior venacava obstruction and hepatic outflow tract obstruction (n=2). Infected cysts were treated by external tube drainage. In cases with intrabiliary rupture exploration of the common bile duct with removal of parasitic debris and/or daughter cysts and drainage of hepatic hydatid cyst was undertaken. The mortality of the complicated hydatid cysts was 18.5 per cent.

It is concluded that once complications develop the mortality and morbidity increases. One should be aware of the possibility of these complications in hepatic hydatid disease.

### P0233 PHARMACOKINETIC ASSESSMENT OF ETHYLCELLULOSE MICROCAPSULES OF MITOMYCIN (MMC) IN PATIENTS

J.A. Goldberg, D.J. Kerr, A. Whateley,
T. Kato, C.S. McArdle.
Royal Infirmary & University of Strathclyde, Glasgow, U.K., and University School of Medicine, Akita, Japan

The potential value of conventional hepatic chemotherapy is limited by systemic toxicity. It is known that the loading of cytotoxic agents into 'slow release' particles, which become trapped in small vessels after arterial administration, increases drug exposure in the target organ and reduces systemic levels in experimental models (1).

The aim of this study was to compare systemic drug exposure following regional administration of either microencapsulated MMC (20 mg) in solution, or MMC (20 mg) by bolus injection.

| | **MMC microcapsules** (mean ± SD) | **MMC in solution** (mean ± SD) |
|---|---|---|
| Clearance ($l\ hr^{-1}$) | 140 ± 31 | 46 ± 8 * |
| Half-life (hrs) | 0.39 ± 0.03 | 0.11 ± 0.02 * |
| Volume distribution (l) | 246 ± 23 | 33 ± 4 * |
| Peak drug concentration ($ng\ ml^{-1}$) | 80 ± 75 | 812 ± 423 * |

(* $p < 0.05$, Student's t-testing)

These results demonstrate that there is very little systemic exposure associated with microencapsulated mitomycin C. Dose escalation should be feasible without increasing toxicity side-effects.

1. Kerr, DJ, Willmot N, Lewi H, and McArdle CS. The pharmacokinetics and distribution of adriamycin-loaded albumin microspheres following intra-arterial administration. Cancer 1988; 62: 878-882.

### P0234 POSSIBILITIES OF US-GUIDED PROCEDURES IN TREATMENT OF SURGICAL DISEASES OF HEPATO-PANCREATO-BILIARY AREA

E. Galperin, F. Nasirov, G. Akhaladze
1st Moscow Medical Institute, USSR

US-guided percutaneous procedures were performed in 278 patients: for acute cholecystitis and obstructive jaundice (126), for cysts and abscesses of liver and the surrounding area (111) and for cysts and abscesses of pancreas (41). Age of more than 80% of the patients with acute cholecystitis and obstructive jaundice was over 65 years, 75 patients had serious cardio-respiratory and vascular disorders. In all cases we used puncture and line probes of echochamber ALOCA-280 (Japan). 37 patients were treated by one-step percutaneous puncture of gallbladder, 64 by one-step draining with the aid of a stylette-catheter, 25 by percutaneous draining according to Seldinger technique. Selection of the method depended on gravity of the patient's general state, degree of biliary hypertension and topographic peculiarities of gallbladder situation. All the complications (3.9%) were observed in our early series. There were two deaths (1.6%) as a result of the very grave initial state of the patients. Percutaneous treatment of abscesses of the liver and the surrounding area was effective in 83 patients (97.6%), only in two of them surgery was needed. No complications were observed. Complete obliteration of non-parasitic cysts of the liver was achieved in 21 patients after percutaneous drainage and sclerotization with alcohol, and in five others dimensions of cyst reduced to 2-3 cm (remote results up to 2.5 years). Percutaneous puncture and drainage of pancreatic cysts proved to be less traumatizing to the patient than surgery, relatively simple and highly effective. It achieves complete obliteration of the cyst, separated from the pancreatic duct.

